# Emerging progress in ruthenium-catalyzed transfer and asymmetric transfer hydrogenation

**DOI:** 10.1039/d6ra00671j

**Published:** 2026-07-20

**Authors:** S. M. Wahidur Rahaman

**Affiliations:** a Assistant Professor, Dept. of Chemistry, Panchakot Mahavidyalaya P.O. - Neturia, Dist. - Purulia Sarbari 723121 West Bengal India wrahaman@gmail.com

## Abstract

Ruthenium-catalysed transfer hydrogenation (TH) and asymmetric transfer hydrogenation (ATH) are well recognised as efficient and environmentally benign methods for synthesising chiral alcohols, amines, and key intermediates used in the pharmaceutical and fine chemical industries. In particular, Ru-catalysed ATH combined with dynamic kinetic resolution (DKR) allows for the stereoconvergent synthesis of complex chiral compounds from racemic carbonyl substrates with high efficiency and excellent enantioselectivity. Despite these significant advances, important challenges remain in achieving precise control over both regioselectivity and stereoselectivity of multifunctional chemical systems. Overall, this review summarises advances in TH and ATH from 2020 to 2025, focusing on catalyst design, substrate scope, mechanistic insights, and methodology development to guide the rational design of advanced ruthenium-based catalysts for the efficient synthesis of enantioenriched alcohols and complex molecules.

## Abstract 2

Ruthenium-catalysed transfer hydrogenation and asymmetric transfer hydrogenation provide efficient and sustainable routes to chiral alcohols, amines, and pharmaceutical intermediates with high activity and enantioselectivity.

## Graphical abstract

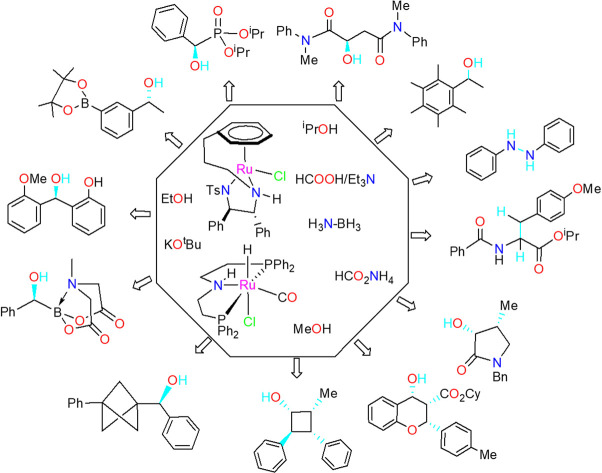

## Introduction

**Fig. 1:**
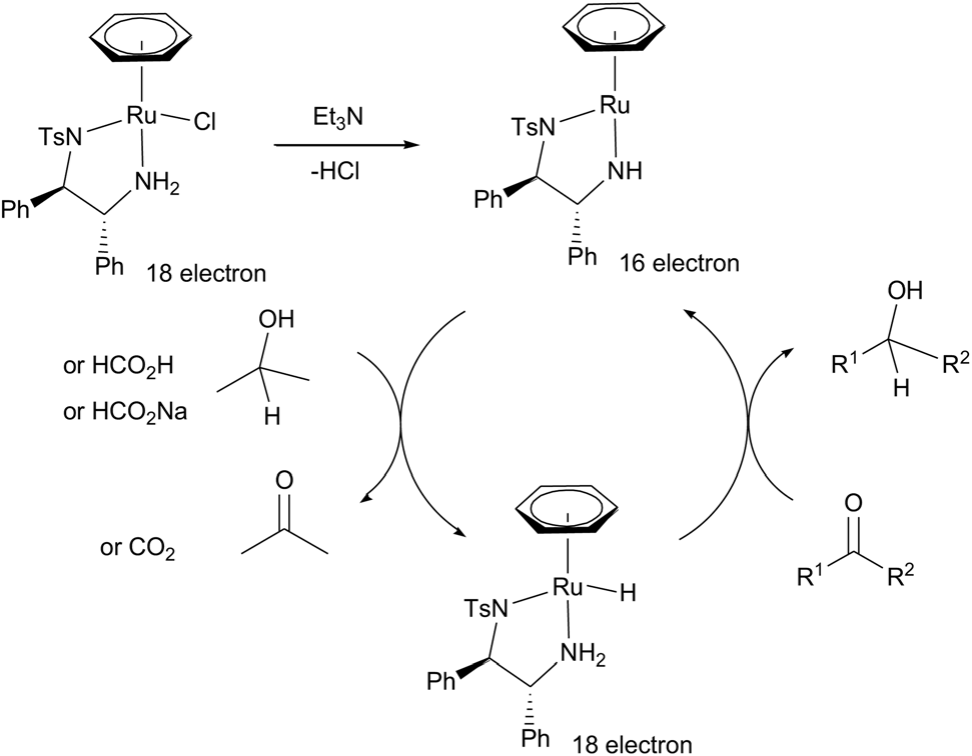
Mechanism of ATH of the Noyori–Ikariya catalyst.

Transfer hydrogenation (TH) and asymmetric transfer hydrogenation (ATH) are vital processes for synthesising organic alcohols, amines, and other intermediates used in drug development, natural product synthesis, fine chemicals, agrochemicals, fragrances, and materials.^1–21^ This method is user-friendly as it occurs under mild and safe conditions, utilising easily accessible hydrogen sources such as isopropanol, sodium formate/water, or azeotropic mixtures of formic acid/triethylamine.^15,22,23^ Its high chemoselectivity makes ATH particularly important in the industrial production of various chiral products. The pioneering work was established in this field by Noyori and his colleagues when they synthesised Ru(ii) complexes with η^6^-arene and a chiral diamine ligand (TsDPEN), which acted as a bifunctional catalyst.^24–28^ This catalyst effectively reduced ketones and imines in the presence of formic acid/triethylamine in an isopropanol solution. The mechanism involved metal–ligand cooperativity, where the Ru–H and N–H bonds transferred hydride and protons to the substrate (Fig. 1). To further enhance the catalytic activity and efficiency, the tethered Ru(ii) catalysts and a diamine ligand was linked to the arene ring. This linkage restricts conformational flexibility, which improves enantioselectivity, stability, and yield. These advancements were applied to a broader range of substrates, including more complex and functionalized molecules.^29^

Subsequently, Ru, Rh, and Ir complexes with tethered ligands and different arene derivatives were extensively employed.^30–56^ Another important advancement is the when ATH coupled with dynamic kinetic resolution (ATH–DKR), which has been widely used to produce chiral molecules with multiple stereocenters from racemic substrates. Racemic substrates, such as alpha-ketoesters, beta-ketoaldehydes, and beta-substituted ketones, can be effectively reduced to yield alcohol products with high efficiency and excellent enantioselectivity through the ATH–DKR process.^57–76^ In the ATH–DKR process, it is essential to maintain a balance between the recemization and reduction rates of the substrate. This balance is controlled by the ruthenium catalyst and additives, either directly or cooperatively. Bifunctional Noyori–Ikariya-type catalysts are particularly effective in this regard due to their ability to mediate both hydride and proton transfer with high precision. Additives such as bases or Lewis acids can further promote racemization and enhance overall reaction efficiency.^77,78^ As a result, ATH–DKR methodologies offer high atom economy and excellent step economy, making them highly attractive for the synthesis of structurally complex molecules.

This review highlights recent advances in TH and ATH chemistry, with particular emphasis on substrate scope, mechanistic understanding, and the development of highly efficient, regioselective, and stereoselective catalytic systems. In addition, growing attention is being directed toward improving the sustainability and industrial applicability of these transformations. While significant progress has been made in the selective reduction of single carbonyl systems, a major ongoing challenge remains the controlled and highly selective transformation of substrates containing multiple carbonyl groups, which is also addressed in this review.

## Catalyst design in ruthenium-catalyzed TH and ATH

The design of ruthenium complexes for catalysis plays a crucial role in controlling selectivity and catalytic activity, with significant applications demonstrated over the years. Over time, catalyst development has evolved from simple metal salts to highly sophisticated molecular architectures, resulting in significant improvements in efficiency, stability, and stereocontrol. A major milestone in this field was the development of Noyori–Ikariya-type Ru(ii) complexes, such as [RuCl(η^6^-arene) (TsDPEN)], which exhibit excellent catalytic performance in both TH and ATH reactions.^30,31,33^ Mechanistic investigations have established that these systems operate through a bifunctional pathway, where hydride transfer from the metal and proton transfer from the ligand occur in a concerted and cooperative manner. In this context, the η^6^-arene ligand plays an essential role by stabilizing the transition state through CH–π interactions, thereby enhancing catalytic efficiency and enantioselectivity. Subsequent developments introduced substituted arene-based ruthenium complexes, and a particularly important advancement was the design of tethered Ru(ii) catalysts, in which the arene ligand is covalently linked to the ligand backbone *via* an alkyl spacer.^30–32^ This structural modification significantly enhances catalyst stability and has proven especially effective in DKR processes within ATH reactions. Further progress in catalyst design includes the incorporation of N-heterocyclic carbene (NHC) ligands,^79,80^ which increase electron density at the ruthenium center. This electronic enrichment facilitates hydride transfer, thereby improving reaction rates and enabling the reduction of less reactive substrates. In addition, pincer-type ligands such as PNN, PNP, and CNN systems provide a rigid tridentate coordination environment that enhances catalyst robustness.^81,82^ These systems operate *via* cooperative metal–ligand mechanisms, allowing reversible hydride and proton transfer processes that are highly effective in hydrogenation chemistry. More recently, nitrogen-rich ligands such as triazoles have been explored due to their tunable electronic and steric properties. These ligands enable efficient catalysis under mild and environmentally benign conditions, further expanding the scope of ruthenium-catalyzed transformations.^83,84^ An important aspect influencing catalytic performance is the presence of non-covalent interactions, including CH–π interactions, hydrogen bonding, and lone pair–π interactions. These weak interactions play a decisive role in stabilizing transition states and controlling stereochemical outcomes, thereby providing valuable insights for rational catalyst design.^85–87^ In addition, the use of additives such as LiCl or Cu(OAc)_2_ has been shown to modulate intermediate coordination environments and influence product selectivity.^77,78^ These additives can fine-tune catalytic pathways and improve overall reaction efficiency. Finally, modern developments in this field strongly emphasize the principles of green chemistry. The use of base-free conditions, solvent-free or aqueous systems, moisture- and air-stable catalysts, and low catalyst loadings significantly enhances the sustainability and practicality of these transformations. Therefore, these TH and ATH processes are increasingly suitable for large-scale and industrial applications, combining high yield with environmental suitability.^48^

## Achiral transfer hydrogenation

**Scheme 1:**
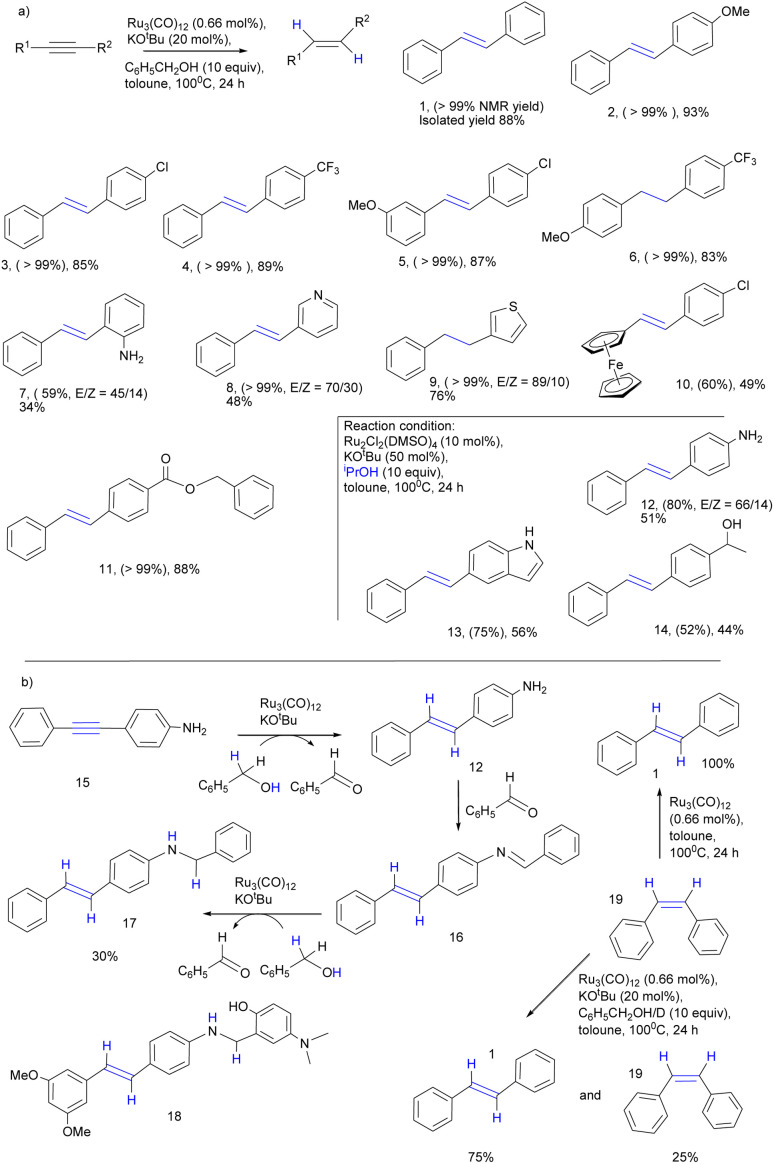
(a) Substrate scope of the semi hydrogenation of alkynes. (b) Mechanistic detail of this reaction. Reproduced from ref. 88 with permission from the American Chemical Society. Copyright 2020.

Nina Kann *et al.*^88^ (2020) reported an efficient method for the synthesis of *E*-alkenes from alkynes *via* a semi-hydrogenation process using the commercially available Ru_3_(CO)_12_ catalyst with benzyl alcohol or isopropanol as hydrogen sources. The reaction proceeds under mild conditions without the need for an external base or ligand, making it an operationally simple and sustainable protocol. This transformation afforded excellent conversions (>99% by NMR) and high isolated yields (up to 93%). A broad range of diaryl alkynes bearing both electron-donating and electron-withdrawing substituents was well tolerated (Scheme 1). Notably, in the case of *para*-amino-substituted alkynes, the reaction furnished the expected *Z*-alkene along with a side product. This was attributed to the *in situ* formation of an aldehyde from benzyl alcohol, which reacted with the amine to generate an imine intermediate. Subsequent reduction of this imine through hydrogen transfer from benzyl alcohol produced the corresponding amine, indicating a hydrogen-borrowing pathway. This strategy was further applied to the synthesis of resveratrol derivatives, which are of interest due to their potential therapeutic relevance in Alzheimer's disease. When RuCl_2_(DMSO)_4_ was used with isopropanol as the hydrogen source, the hydrogen-borrowing pathway was not observed, and only the alkene product was obtained. Steric effects also influenced the reaction outcome. Substrates bearing *ortho*-amino substituents showed reduced reactivity, likely due to steric hindrance around the reactive site. Similarly, 3-(phenylethynyl)pyridine required longer reaction times and higher catalyst loading, which was attributed to coordination of the pyridine nitrogen to the ruthenium centre, thereby slowing the catalytic cycle. Heteroaryl alkynes such as thiophene derivatives performed very well, affording the corresponding *E*-alkenes in excellent yields (>99%). Substrates containing ferrocene or ester functionalities were also successfully reduced, giving moderate to good yields. Interestingly, under RuCl_2_(DMSO)_4_/isopropanol conditions, simultaneous reduction of both alkynes and ketones was observed, leading to more complex hydrogenated products, including reduced indole derivatives. Mechanistic investigations based on NMR studies of diphenylacetylene in the presence of benzyl alcohol and the ruthenium catalyst showed that *Z*-alkene forms initially and subsequently isomerize to the more stable *E*-alkene. Additional experiments using *Z*-stilbene and deuterated benzyl alcohol (Bn-OD) revealed no deuterium incorporation, but instead a mixture of *E*- and *Z*-stilbene was obtained. In contrast, using only the catalyst led exclusively to the *E*-isomer. These results suggest that the isomerization does not proceed *via* simple hydrogenation–dehydrogenation, rotation, or β-hydride elimination pathways, indicating a more complex catalytic process.

**Scheme 2:**
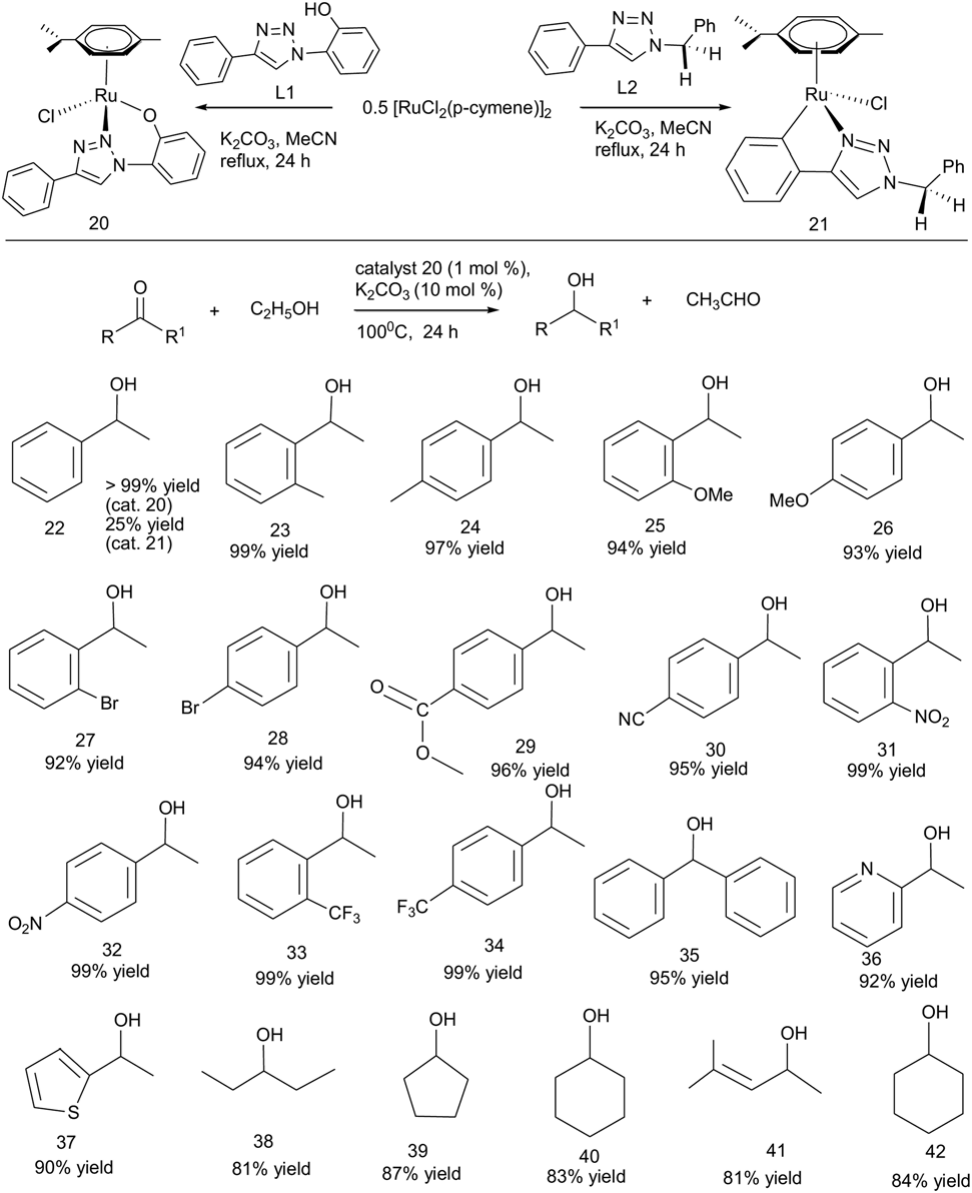
Synthesis of ruthenium-triazole complexes 20 and 21 and transfer hydrogenation of ketones using ethanol catalyzed by complex 20. Reproduced from ref. 89 with permission from the American Chemical Society. Copyright 2021.

**Scheme 3:**
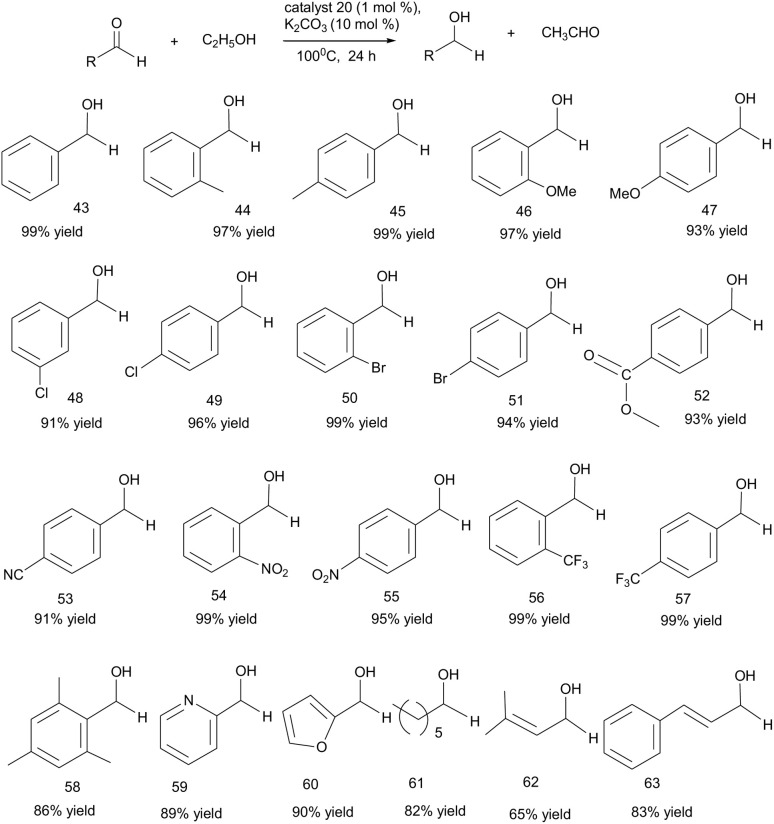
Transfer hydrogenation of aldehydes using ethanol catalyzed by 20 (adapted from ref. 89).

Bidraha Bagh^89^ (2021) reported the synthesis and catalytic application of air-stable ruthenium complexes bearing 1,2,3- triazole-based ligands for efficient TH of aldehydes and ketones using methanol and ethanol as hydrogen sources. The complex [(η^6^-*p*-cymene) RuCl_2_] _2_ was reacted with a 1,4-disubstituted 1,2,3-triazole ligand (L1) in the presence of base to afford a red-coloured complex 20 in good yield (Scheme 2). Similarly, ligand L2 under identical conditions furnished a yellow-coloured complex 21. Both complexes were characterized by ^1^H and ^13^C NMR spectroscopy, and single-crystal X-ray diffraction confirmed that complex 20 adopts a pseudo-octahedral “three-legged piano-stool” geometry. Catalytic evaluation revealed that complex 20 exhibited excellent activity in TH reactions. Acetophenone was fully converted to 1-phenylethanol using 1 mol% catalyst and 10 mol% K_2_CO_3_ in ethanol at 100 °C for 24 hours. Comparable conversion was also achieved using 2 mol% catalyst at 70 °C in 20 hours, demonstrating efficient catalytic performance under relatively mild conditions. In contrast, catalyst 21 showed significantly lower activity, affording only 25% conversion under similar conditions. A wide range of substituted acetophenones was then investigated using catalyst 20. Both electron-donating (Me, OMe) and electron-withdrawing substituents (NO_2_, CF_3_, ester, CN, halogens) at *ortho* and *para* positions were well tolerated, giving excellent to near-quantitative yields of the corresponding alcohols (23–34, 92–99%). These results indicate that electronic effects of substituents have minimal influence on catalytic efficiency. Heteroaryl ketones such as 2- acetylpyridine and 2-benzoylthiophene were also efficiently reduced to the corresponding alcohols in good yields (36 : 92%, 37 : 90%). Benzophenone showed excellent reactivity with 95% yield (35). Aliphatic ketones, including 3-pentanone, cyclopentanone, and cyclohexanone, were successfully reduced to the corresponding alcohols (38–40, 81–87%). α, β-Unsaturated ketones were also selectively hydrogenated, giving 4-methyl-3-penten-2-ol (41, 80%) and cyclohexanol from 2-cyclohexenone (42, 84%). Aldehyde substrates also performed very well in this catalytic system (Scheme 3). Benzaldehyde was quantitatively converted to benzyl alcohol (43, 99%), while both electron-rich and electron-deficient aromatic aldehydes delivered excellent yields (44–57, 91–99%). Even sterically hindered 2,4,6-trimethylbenzaldehyde was efficiently reduced to the corresponding alcohol (58, 96%). Aliphatic and heteroaryl aldehydes such as hexanal, picolinaldehyde, and furfural were also successfully converted to alcohols in good yields (59–61, 82–90%). α, β-Unsaturated aldehydes were selectively reduced, giving moderate to good yields (62: 65%, 63: 83%). Solvent effects were further investigated using methanol, ethanol, and isopropanol. In methanol, a wide range of substrates displayed excellent conversions and yields similar to those obtained in ethanol. However, evaluation using the CHEM21 green metrics toolkit indicated that although methanol gives excellent atom economy, its higher reaction temperature (100 °C) makes it less energy-efficient. Among the tested conditions, isopropanol at 70 °C and ethanol at 70 °C were identified as the most sustainable options. Between these, ethanol was considered the most favourable due to its better atom economy, reaction mass efficiency, and higher environmental adaptability compared to isopropanol. Overall, this study highlights a robust and multifunctional triazole-based Ru catalytic system capable of effectively reducing a wide variety of carbonyl compounds under mild and environmentally benign conditions.

**Scheme 4:**
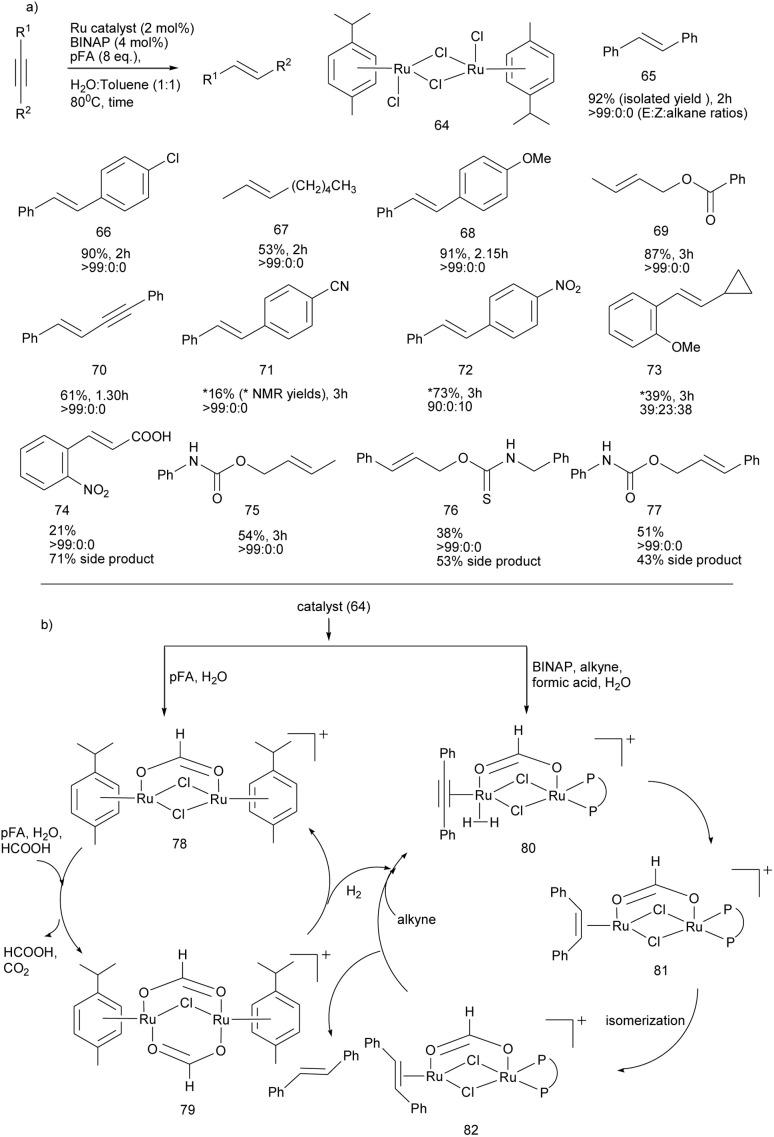
(a) Partial transfer hydrogenation of aliphatic and aromatic alkynes. (b) Proposed pathway for the transfer semi hydrogenation of alkynes. Reproduced from ref. 90 with permission from Wiley-VCH. Copyright 2021.

H. G. Prechtl^90^ (year 2021) was the first to utilise paraformaldehyde as a hydrogen source, which is inexpensive, safe, and readily available. This reagent was employed to partially reduce aliphatic and aromatic alkynes in the presence of the commercially available [Ru(*p*-cymene)Cl_2_]_2_ catalyst, along with the externally added ligand 2,2-bis(diphenylphosphino)-1,1-binaphthyl (BINAP). This reduction process is relatively uncommon in the literature. The *E*-selective product was obtained under mild conditions, in a base-free environment, and through an environmentally benign process. Various types of aliphatic and aromatic alkynes were utilised in the partial hydrogenation; both symmetric aromatic and unsymmetric aromatic-alkyl-containing alkyne compounds were readily reduced in a short reaction time, achieving good to very good yields (Scheme 4). The alkyne with an electron-donating aryl group was reduced more readily than the alkyne with an electron-withdrawing aryl substituent (65–73). Notably, the nitro and ester functional groups in the alkyne were also reduced without being affected. The reduction of the –CN-bearing compound was notably slow, yielding only 16%. This sluggishness is attributed to the coordination of the CN group to the metal, which inhibits catalytic activity (71). The synthesis of nitro cinnamic acid (74), carbamates (75, 77), and thiocarbamates (76) was accomplished in this process. ESI-MS analysis, along with deuterated and controlled experiments, indicated that during the first catalytic cycle, the catalyst (64), in the presence of pFA and water, generated formiato-bridged dimeric compounds 78 and 79. These compounds subsequently produced CO_2_, H_2_, and formic acid. In the second catalytic cycle, the catalyst *in situ* generated formic acid, alkyne, BINAP, and water, leading to the formation of intermediate 80. During this step, the alkyne was hydrogenated *via* intermediate 81, followed by isomerization from the *Z*-alkene to the *E*-alkene, resulting in intermediate 82. The product alkene was then eliminated, and the addition of alkyne and hydrogen produced the active species 80.

**Scheme 5:**
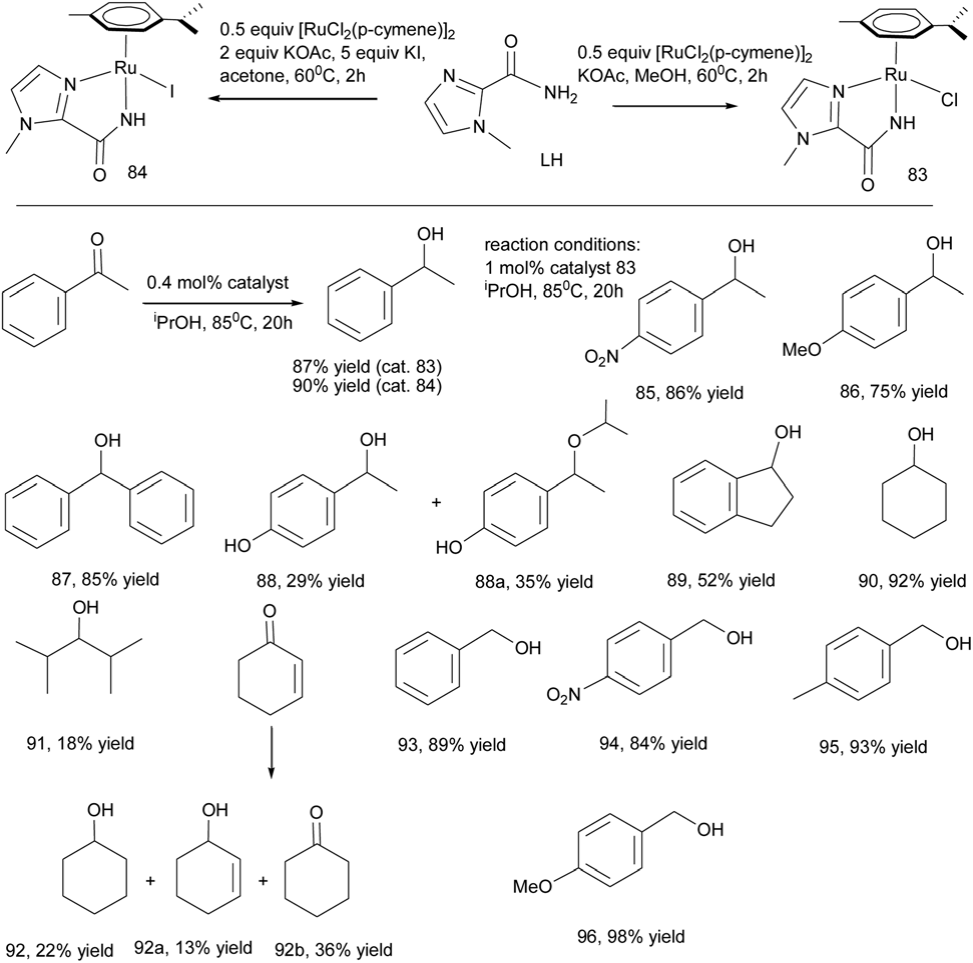
Synthesis of Ru complexes and substrate scope for base-free TH in air catalyzed by 83. Reproduced from ref. 91 with permission from the American Chemical Society. Copyright 2022.

S. E. Kalman *et. al*.^91^ (2022) reported the synthesis of half-sandwich Ru(ii) complexes using *N*-methylimidazole-2-carboxamide (HL) as ligand. The complexes were prepared by reacting [RuCl_2_(η^6^-*p*-cymene)]_2_ with HL in the presence of KOAc in methanol at 60 °C (Scheme 5). The resulting chloride complex, [(η^6^-*p*-cymene)Ru(l)Cl] (83), was obtained in 68% yield. Single-crystal X-ray diffraction confirmed a typical piano-stool geometry, with a Ru-centroid distance of 1.671 Å. In the ^1^H NMR spectrum, the deprotonated amide NH signal appeared at 5.90 ppm, while the imidazolyl protons were observed at 7.26 and 6.89 ppm. Substitution of chloride with iodide under similar conditions afforded [(η^6^-*p*-cymene)Ru(l)I] (84) in 80% yield. The corresponding NMR spectra showed imidazolyl resonances at 7.22 and 6.89 ppm, and the amido NH signal at 5.76 ppm. Both complexes were evaluated in TH reactions using isopropanol as the hydrogen source. Acetophenone was efficiently reduced to 1-phenylethanol using catalyst 83, affording 87% yield after 20 hours at 85 °C without any external base. Catalyst 84 provided a slightly higher yield of 90% under identical conditions; however, due to its lower stability, further studies were focused on catalyst 83. A variety of substituted ketones and aldehydes were then investigated (Scheme 5). Electron-deficient substrates such as 4-nitroacetophenone were reduced smoothly to the corresponding alcohol in 86% yield (85), while electron-rich substrates like 4-methoxyacetophenone afforded a lower yield of 75% (86). Interestingly, 4-hydroxyacetophenone led to a mixture of products, giving 29% of the alcohol (88) and 35% of the corresponding ether (88a), indicating competing side reactions under the reaction conditions. Sterically hindered ketones were also compatible with the catalytic system. Benzophenone and 1-indanone were reduced to the corresponding alcohols in 85% (87) and 52% (89) yields, respectively. Aliphatic ketones showed more variable reactivity, with cyclohexanone giving cyclohexanol in 92% yield (90), whereas diisopropyl ketone afforded only 18% yield (91). Reduction of 2-cyclohexenone produced a mixture of products, including cyclohexanol (22%), 2-cyclohexenol (13%), and cyclohexanone (36%), indicating partial and non-selective hydrogenation (92–92b). Aldehyde substrates were also efficiently reduced. Benzaldehyde afforded benzyl alcohol in 89% yield (93), while electron-withdrawing *para*-nitrobenzaldehyde gave 84% yield (94). Electron-rich substrates such as *p*-methyl- and *p*-methoxybenzaldehyde delivered excellent yields of 93% (95) and 98% (96), respectively. Mechanistically, the reaction is proposed to proceed *via* initial halide dissociation from the ruthenium center, followed by activation of isopropanol to generate a Ru-hydride intermediate. Subsequently, hydride transfers of the active species to the carbonyl group of aldehydes or ketones, producing the corresponding alcohols and completing the catalytic cycle.

**Scheme 6:**
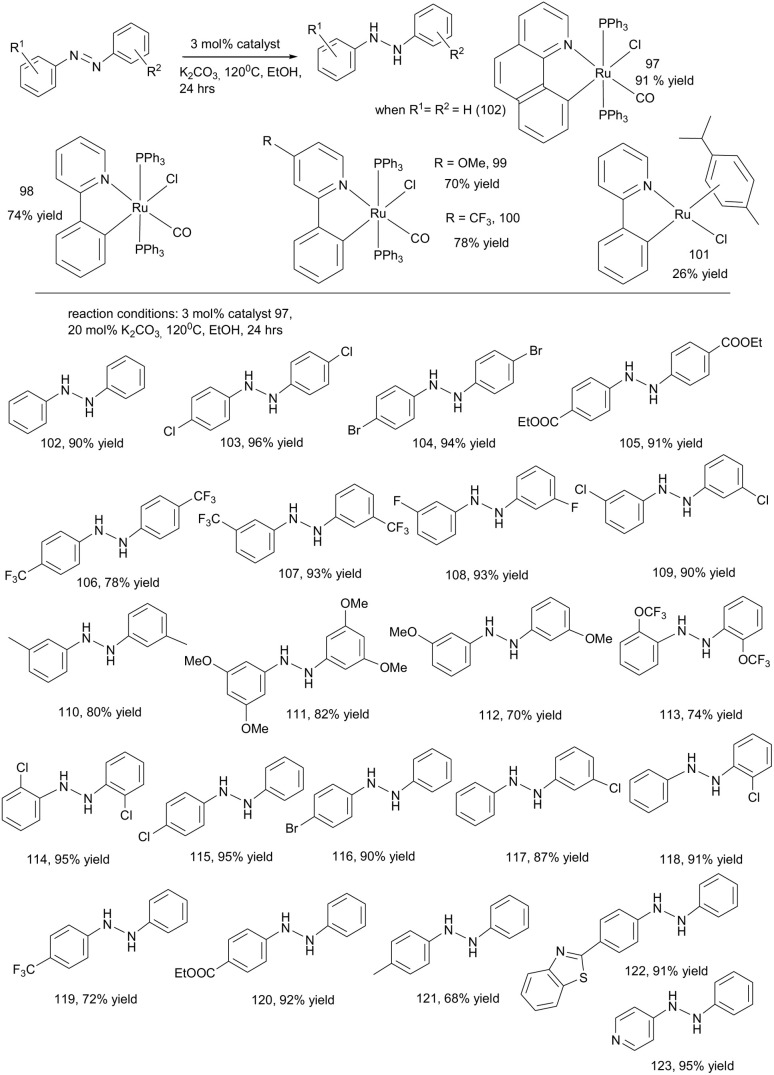
Optimization of the reaction conditions and substrate scope for semihydrogenation of azoarenes. Reproduced from ref. 92 with permission from the American Chemical Society. Copyright 2022.

**Scheme 7:**
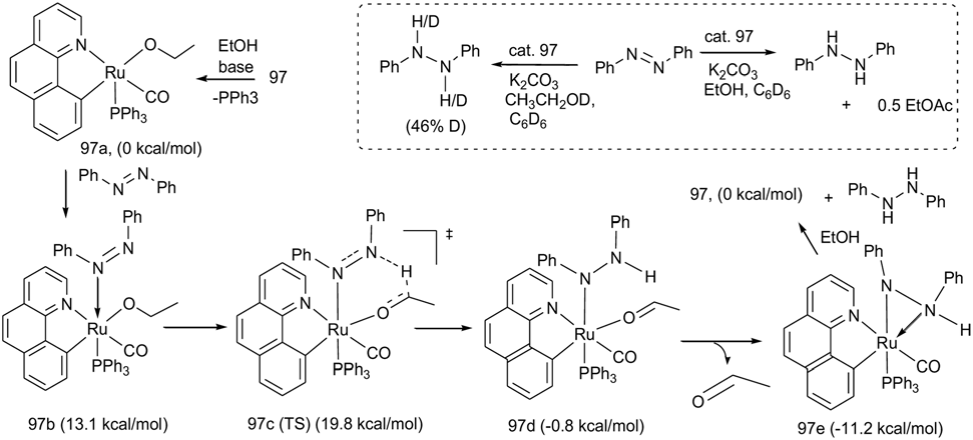
Proposed reaction mechanism with free energy.

D. Gong and Z. Xu^92^ (2022) showed the semihydrogenation of azoarenes to hydrazoarenes using a ruthenium-catalyzed TH system. In this reaction, ethanol was used as the hydrogen source, and potassium carbonate (K_2_CO_3_) was utilized as a weak base. The reaction was conducted at 120 °C for 24 hours using a series of five Ru catalysts (97–101) (Scheme 6). Among the tested catalysts, catalyst 97 (3 mol%) showed the highest catalytic activity, producing the hydrazoarene product in 91% yield. In comparison, other catalysts showed comparatively lower efficiencies (98–101). Sodium carbonate (Na_2_CO_3_) also functioned as a suitable base, delivering up to 85% yield, whereas sodium acetate (NaOAc) failed to promote any conversion. Lowering the reaction temperature to 110 °C reduced the yield to 68%, and decreasing the catalyst loading to 2 mol% led to a yield of 72%. No reaction was observed in the absence of either catalyst or base, confirming the necessity of both components for efficient transformation. A wide range of symmetrical azobenzene derivatives bearing both electron-donating and electron-withdrawing substituents, including halogens, CF_3_, ester, OCF_3_, alkoxy, and alkyl groups, were evaluated under the optimized conditions. *Para*-substituted azobenzenes containing halogen and ester groups afforded hydrazoarenes in high yields (90–96%) (103–105). *Meta*-substituted electron-withdrawing groups also performed well, giving products in 90–93% yield (108–110). The *para*-CF_3_-substituted substrate afforded a slightly reduced yield of 78% (106). Substrates bearing alkoxy and methyl substituents gave moderate yields ranging from 70–82% (110–112). *Ortho*-substituted OCF_3_ and chloro groups produced the corresponding products in 74% and 95% yield, respectively (113–114). Unsymmetrical azobenzenes bearing halogen substituents at different positions (*ortho*, *meta*, and *para*) also showed good reactivity, affording products in 87–91% yield (115–117). Similarly, substrates containing ester, CF_3_, and methyl groups provided moderate to good yields (68–92%) (119–121). Heteroaromatic azobenzenes, including pyridine- and thiazole-containing systems, were efficiently reduced to the corresponding hydrazo compounds in 91–95% yield (122–123). Mechanistic investigations were carried out to understand the reaction pathway (Scheme 7). Deuterium-labelling experiments revealed that ethanol is the hydrogen source. In a reaction conducted in C_6_D_6_, formation of ethyl acetate was observed by ^1^H NMR spectroscopy, while using CH_3_CH_2_OD resulted in 46% deuterium incorporation into the hydrazoarene product, confirming direct hydrogen transfer from ethanol. Further mechanistic insight was obtained through DFT studies. The results suggested that hydrogen transfer proceeds predominantly *via* a Meerwein–Ponndorf–Verley (MPV)-type pathway rather than through a Ru–H intermediate, due to a lower activation barrier. The catalytic cycle begins with the formation of a Ru–alkoxide intermediate (97a), followed by coordination of azobenzene to form intermediate 97b (Δ*G* = 13.1 kcal mol^−1^). Hydrogen transfer to the nitrogen atom of azobenzene *via* a six-membered transition state (97c), with an activation barrier of 19.8 kcal mol^−1^, which is the rate-determining step, leading to intermediate 97d. Next steps involve the elimination of an aldehyde species to form 97e, followed by the regeneration of the active catalyst upon the coordination of ethanol, finally producing the hydrazoarene product.

**Scheme 8:**
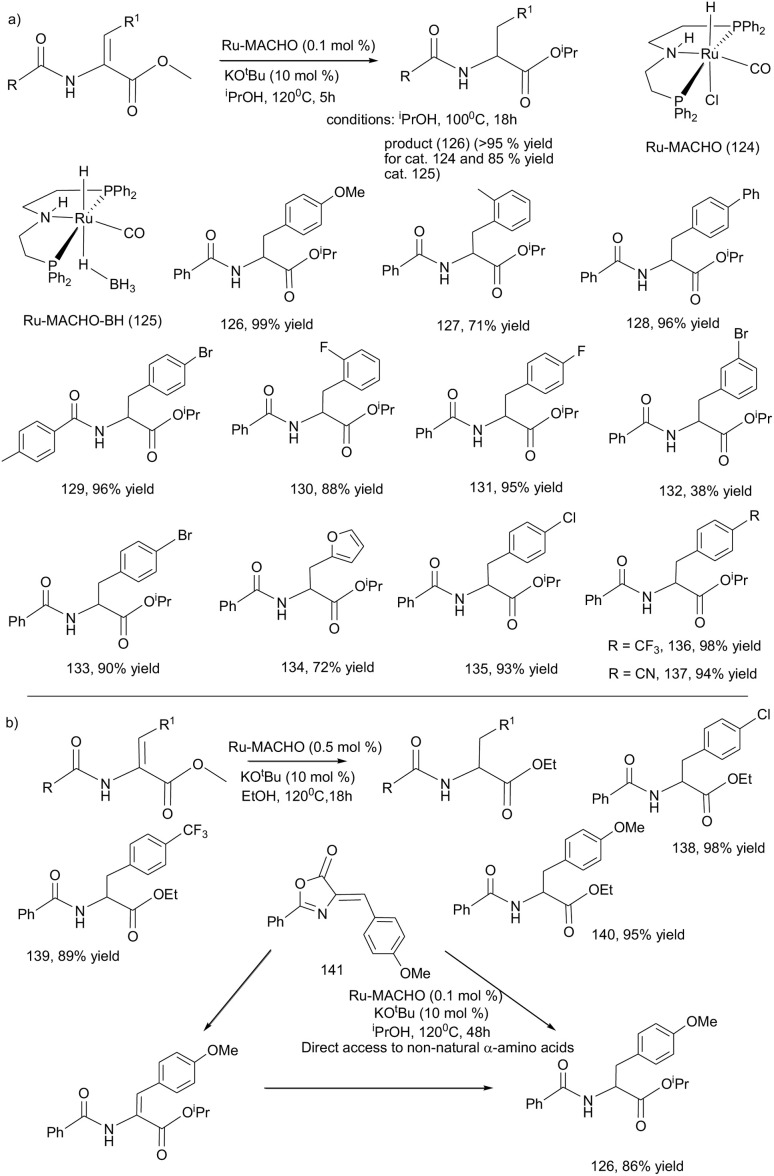
Substrate scope of the transfer hydrogenation reaction (a) using ^*i*^PrOH as H-donor. (b) Using EtOH as H-donor and one-pot synthesis of α-amino acids. Reproduced from ref. 93 with permission from the American Chemical Society. Copyright 2022.

M. Nielsen and co-workers^93^ (2022) showed the use of ruthenium pincer catalysts for the chemo-selective TH of enamides to access α-amino acid derivatives. The study used PNP-type Ru complexes, Ru–MACHO (124) and Ru–MACHO-BH (125), in the presence of isopropanol (^*i*^PrOH) as the hydrogen donor (Scheme 8). The enamide reduction was conducted using 1 mol% of catalyst 124 in the presence of 10 mol% KO^*t*^Bu at 100 °C for 18 hours, producing >95% conversion. In contrast, catalyst 125 showed a lower conversion of 85% under similar conditions, indicating the excellent efficiency of Ru–MACHO (124). Among various bases screened (LiO^*t*^Bu, NaOEt, NaOMe, and KO^*t*^Bu), potassium *tert*-butoxide (KO^*t*^Bu) proved to be the most effective. The reaction remained highly efficient even when the catalyst loading was reduced to 0.5 mol%, and full conversion could still be achieved under slightly modified conditions. Increasing the temperature to 120 °C significantly accelerated the reaction, reducing the reaction time to approximately 3 hours. Even at a very low catalyst loading (0.1 mol%), complete conversion was achieved within 5 hours at elevated temperature, demonstrating the high robustness and efficiency of the catalytic system. A broad range of enamide substrates was then explored. *Para*-substituted aryl enamides bearing electron-donating groups such as methoxy and phenyl afforded excellent yields of α-amino acid esters (126, 99%; 128, 96%). *Ortho*-methyl substitution on the aromatic ring slightly reduced the efficiency, giving product 127 in 71% yield. Electron-withdrawing substituents such as Br, F, and Cl at various positions on the phenyl ring were well tolerated, delivering the corresponding products in good to excellent yields (129–135, 88–96%). However, a *meta*-bromo substituent resulted in a significantly reduced yield (132, 38%), while a *meta*-methyl-substituted substrate showed no reactivity. Heteroaryl substrates, such as furan-containing enamides, also underwent successful reduction, affording product 134 in 72% yield. Strong electron-withdrawing groups like CF_3_ and CN at the *para* position performed exceptionally well, giving high yields (136, 98%; 137, 94%). Solvent effects were also investigated. A similar reaction was carried out with ethanol solvent, but it required longer reaction times and higher catalyst loading to get comparable conversion. There was no reaction when methanol or formic acid was used as a solvent. Nevertheless, in ethanol, *para*-substituted substrates containing Cl, CF_3_, and OMe groups still gave good to excellent yields (138–140, 89–98%). Finally, the methodology was successfully applied on a gram scale, affording 93% yield without loss of efficiency. A one-pot conversion of Erlenmeyer–Plöchl azlactone (141) further explained the synthetic utility of the method, resulting in a protected α-amino acid product (126) in 86% yield.

**Scheme 9:**
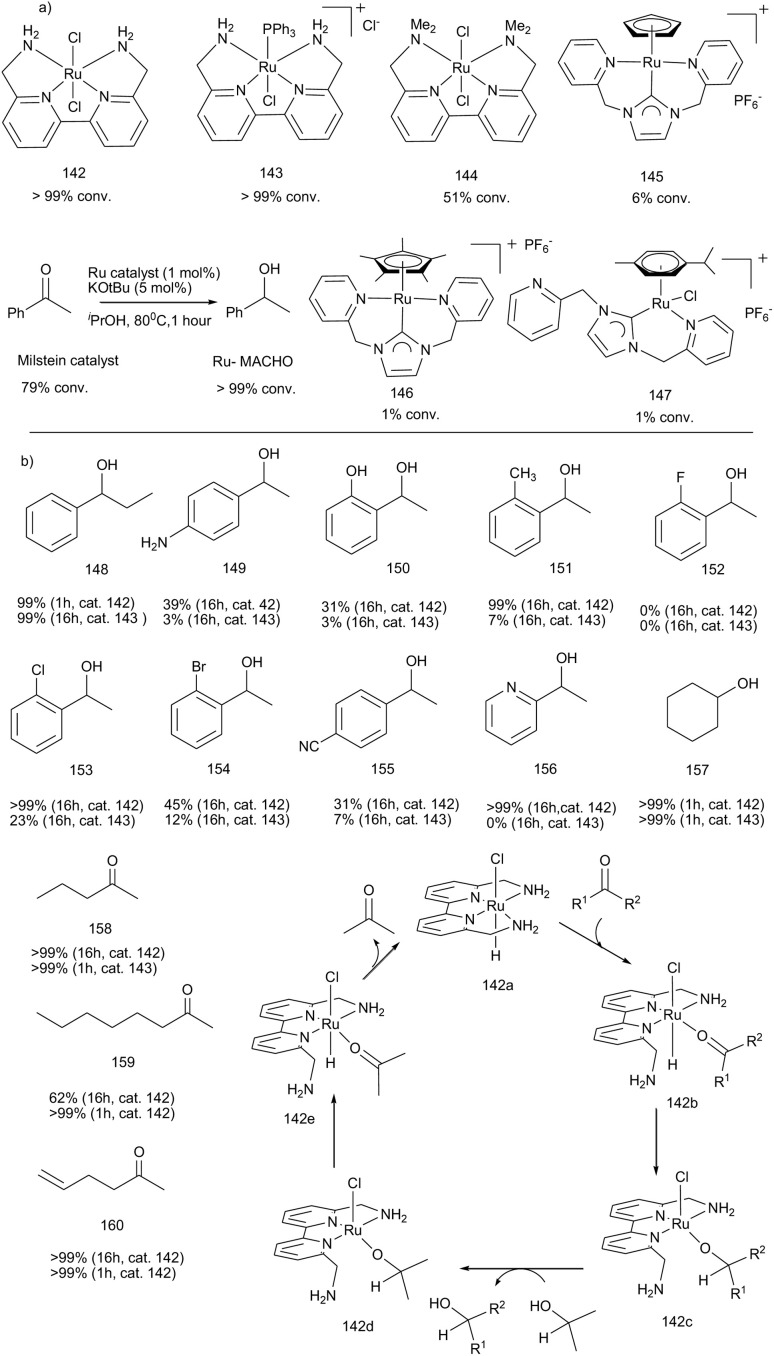
(a) Optimized catalyst in TH reaction. (b) Substrate scope of TH and reaction mechanism. Reproduced from ref. 94 with permission from the Royal Society of Chemistry. Copyright 2022.

J. W. Walton and co-workers^94^ (2022) reported a series of bipyridine- and N-heterocyclic carbene-based ruthenium complexes and evaluated their catalytic performance in TH reactions. The complexes (142–147), were thoroughly characterized using NMR spectroscopy, ESI-MS, and single-crystal X-ray crystallography. The catalytic efficiency of these systems (1 mol%) was compared for the TH of acetophenone in 2-propanol using KO^*t*^Bu (5 mol%) at 80 °C for 1 hour. Among all catalysts tested, complexes 142, 143, and the Milstein catalyst exhibited excellent activity, achieving >99% conversion. In contrast, the remaining catalysts showed only moderate to low activity, with conversions ranging from 1% to 79% (Scheme 9). Further studies explored the substrate scope using catalysts 142 and 143. Propiophenone was readily reduced by both catalysts, giving the corresponding alcohol in 99% yield with catalyst 142 in 1 hour, whereas catalyst 143 required 16 hours to reach full conversion. Substituent effects were also significant: 4-amino acetophenone showed only 39% conversion with catalyst 142 and 3% with catalyst 143, while *ortho*-hydroxy acetophenone gave similarly poor conversion (31%) due to unfavorable coordination effects. In contrast, *ortho*-methyl substitution proved beneficial, affording quantitative conversion (99% yield). Interestingly, 2-fluoro acetophenone was unreactive under the conditions, whereas 2-chloro- and 2-bromo acetophenone gave 99% and 45% yields, respectively. *Para*-cyano acetophenone also showed very low reactivity. Heteroaryl substrates such as pyridyl ketones were efficiently reduced with catalyst 142. Cyclic ketones performed well with both catalysts, while aliphatic ketones were converted quantitatively with catalyst 143 within 1 hour, compared to 16 hours required by catalyst 142. In summary, aromatic and heteroaromatic ketones reacted more efficiently with catalyst 142, while catalyst 143 showed better performance with aliphatic ketones, indicating that subtle changes in the ruthenium structure led to significant alterations in catalytic activity. To elucidate the mechanism of the reaction, kinetic analysis of acetophenone was conducted using ^1^H NMR spectroscopy, which revealed a zero-order dependence on acetophenone, suggesting substrate coordination is not a rate-determining step. Further Eyring analysis, plotting the rate of reaction against temperature, suggested that the reaction occurred *via* an inner-sphere mechanism. In the first step, in the presence of KO^*t*^Bu, 2-propanol formed a ruthenium alkoxide intermediate (142a). This intermediate underwent β-hydride elimination to produce a hydride intermediate (142b). Subsequently, the –NH_2_ ligand detached from the metal, allowing the substrate ketone to coordinate with the metal, which formed intermediate 142c. In the next step, hydride insertion occurred into the ketone, resulting in intermediate 142d. The presence of isopropanol, led to the final alcohol product. Another β-hydride elimination generated intermediate 142e, ultimately resulted in the elimination of acetone regenerating the active alkoxide catalyst, 142a.

**Scheme 10:**
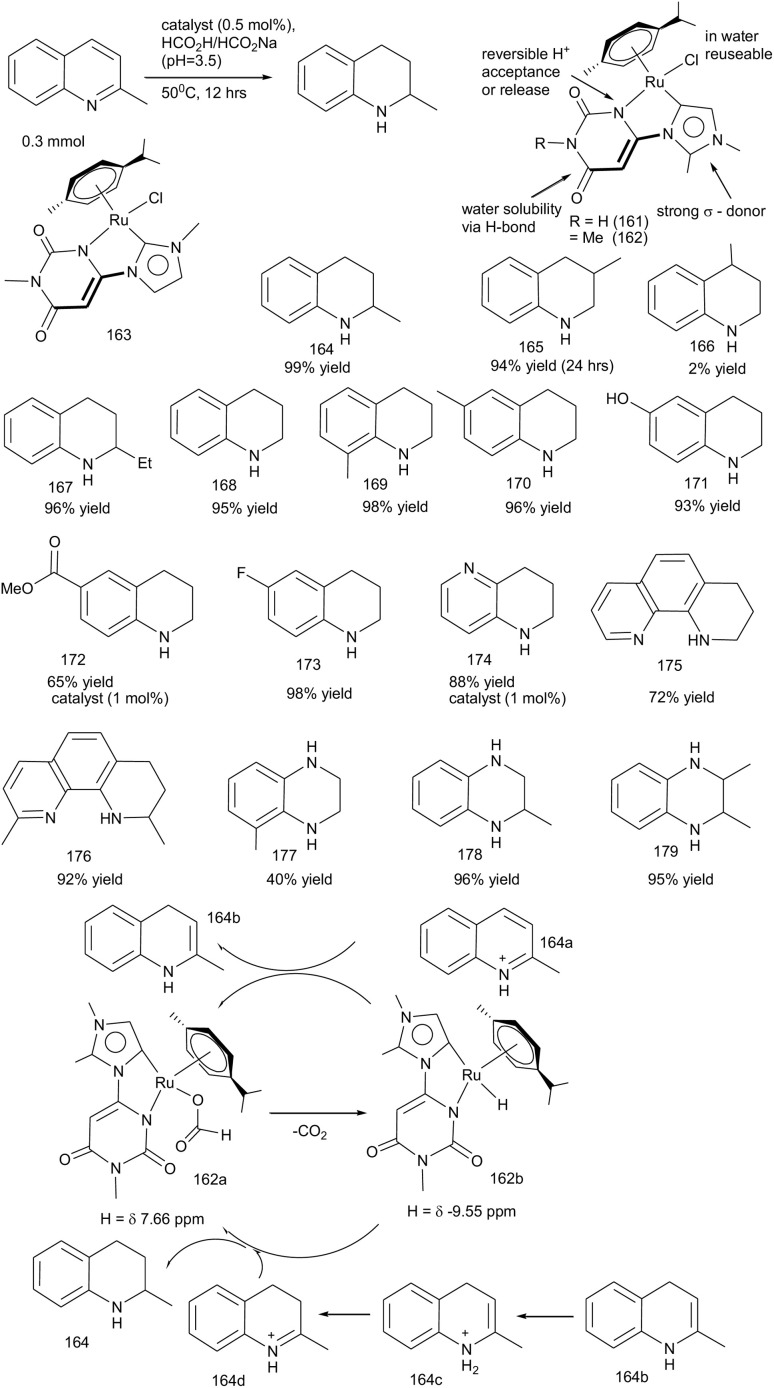
Examples of TH products and plausible catalytic steps for the TH. Reproduced from ref. 95 with permission from the Royal Society of Chemistry. Copyright 2022.

Joyanta Choudhury^95^ (year 2022) *et al.* reported a water-soluble Ru–NHC-based catalyst employed in the TH reaction of various quinoline-type N-heteroarenes in a formic acid aqueous medium. This multifunctional ligand backbone provided excellent durability, reusability, and compatibility in catalytic reactions. The 161, 162, and 163 complexes were synthesized using NHC and aNHC ligands, which were characterized by various spectroscopic methods (Scheme 10). Using these catalysts, the TH reaction was carried out with 2-methylquinoline, yielding derivatives of 1,2,3,4-tetrahydroquinolines. The best results were achieved with catalyst 162, which contains an aNHC ligand that created more electron density at the metal centre, which helps to promote more catalytic activity. With the 162 catalyst, 2-methylquinoline and 3-methylquinoline substrates were smoothly reduced with excellent yields of 99% and 94%, respectively (products 164 and 165); however, 4-methylquinoline showed extremely low catalytic activity, yielding only 2% (product 166). This poor result is attributed to the steric hindrance between the methyl group of the substrate and the *para*-cymene methyl group of the catalyst, as supported by DFT calculations. Methyl groups on the fused phenyl ring of quinolines were tolerated in this reaction, yielding good results (169–170). Other functional groups, such as –OH, –COOMe, and –F on the phenyl ring, also tolerated reduction in the TH reaction with very good yields (171, 172, 173). Additionally, extended heteroarenes such as 1,5-naphthyridine, 1,10-phenanthroline, demonstrated very good activity in the TH reaction (174–176), while quinoxaline derivatives tolerated this reaction well, resulting in high yields (177–179). This catalyst could be used repetitively for up to four cycles while maintaining its effectiveness, indicating its overall robustness and recyclability. Furthermore, a 10 days-old used catalyst showed the same level of activity in the TH reaction, reflecting its durability. Mechanistic studies supported by DFT calculations revealed that the reaction proceeds through formation of a Ru-hydride intermediate (162b) from a Ru–OOCH species (162a), with an activation barrier of 12.7 kcal mol^−1^. The hydride is then transferred to protonated quinoline, with preference for the 4-position (activation barrier 7.9 kcal mol^−1^) over the 2-position (9.4 kcal mol^−1^). This leads to the formation of a 1,4-dihydroquinoline intermediate, which subsequently undergoes protonation and isomerization to a 1,2-iminium species. A second hydride transfer then furnishes the final 1,2,3,4-tetrahydroquinoline product. Overall, this study demonstrates an efficient, water-compatible, and recyclable Ru–NHC catalytic system for the selective reduction of quinoline derivatives under mild and sustainable conditions.

**Scheme 11:**
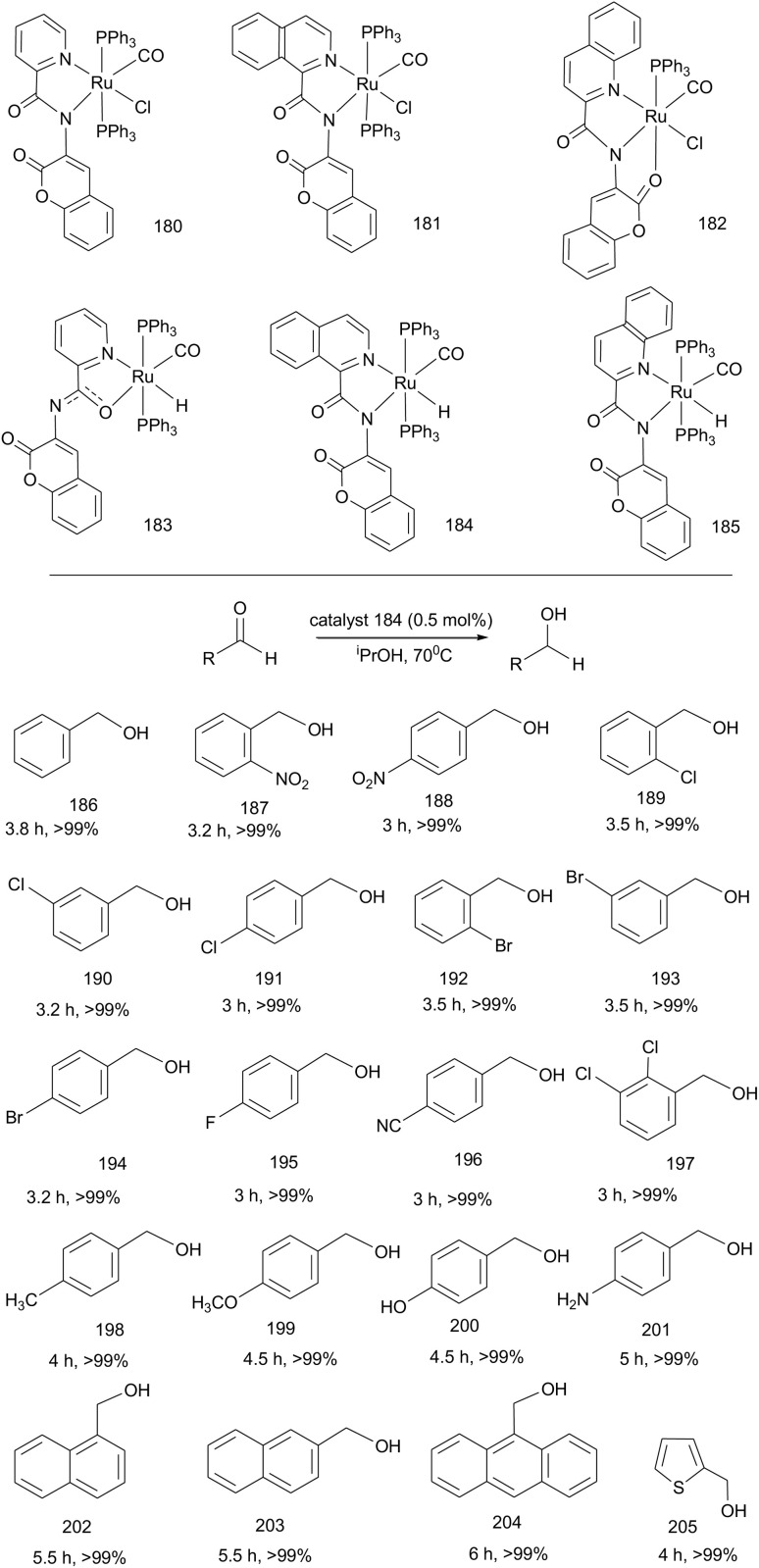
Ru(ii) complexes (180–185) synthesized and substrate scope for the base-free TH of assorted aldehydes catalyzed by complex 184. Reproduced from ref. 96 with permission from the American Chemical Society. Copyright 2023.

**Scheme 12:**
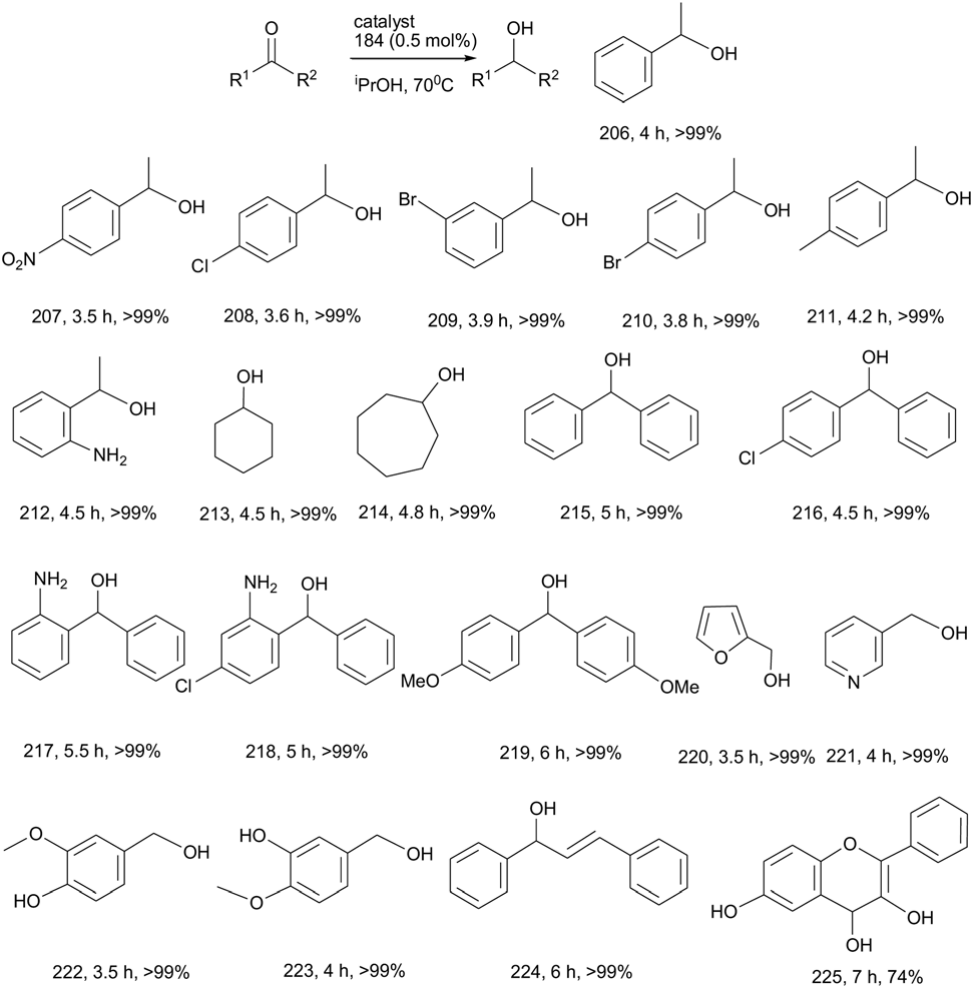
Substrate scope for the base-free TH of assorted ketones catalyzed by complex 184. Reproduced from ref. 96 with permission from the American Chemical Society. Copyright 2023.

R. Gupta's group^96^ (year 2023) synthesized a variety of coumarin-amide ligands and their corresponding complexes, including [Ru–Cl] and [Ru–H] complexes (180–185) (Scheme 11). All the complexes were characterized by NMR, IR, ESI-MS, and UV-vis spectroscopy. In the ^1^H NMR spectra, the hydride peak appeared at −10.09 ppm, −11.93 ppm, and −11.41 ppm of complexes 183, 184, and 185, respectively. In the ^31^P NMR spectra all complexes showed phosphorus NMR signals in the range of 44.34–46.68 ppm; complex 182 showed slightly shifted signal at 46.33 ppm due to the presence of only one PPh_3_. In the ^13^C NMR spectra, the carbonyl (CO) peak appeared around 200 ppm, and the stretching frequencies for CO are in the range of 1914–1936 cm^−1^ in the IR spectra. The molecular structures of complexes exhibit a distorted octahedral geometry, which was confirmed by single X-ray crystallography. Catalytic activity showed that the [Ru–Cl] complexes (180–182) exhibit low activity in the absence of base; however, in the presence of a base like NaOH, KOH, KO^*t*^Bu, and Et_3_N, the yields increased significantly, reaching between 80% and 90%, with KOH providing the highest yield. In contrast, the [Ru–H] complexes (183–185) showed excellent activity even without any base, achieving yields of 95% to 99%. A variety of solvents were also tested, including methanol, ethanol, 1-propanol, 2-propanol, and 1-butanol; the best yield was achieved using isopropanol as the solvent. Among all catalysts tested, complex 184 showed the highest activity and was selected for further investigations. Isopropanol was identified as the optimal solvent, outperforming methanol, ethanol, 1-propanol, 2-propanol, and 1-butanol. Using catalyst 184, a broad range of benzaldehyde derivatives underwent efficient TH. Electron-deficient substrates, including nitro-, halo-, and cyano-substituted benzaldehydes (4-nitro, chloro-, bromo-, fluoro-, and dichloro derivatives), were reduced more rapidly (187–188) than electron-rich substrates such as methyl-, methoxy-, hydroxy-, and amino-substituted analogues (199–201). This enhanced reactivity of electron-poor aldehydes was attributed to more favorable hydride insertion into the polarized carbonyl group. Sterically demanding substrates, including 1-naphthaldehyde, 2-naphthaldehyde, and 9-anthraldehyde, were also smoothly reduced to the corresponding alcohols (202–204), as was the heteroaryl substrate thiophene-2-carbaldehyde, which underwent complete conversion (205). Following aldehyde reduction, aromatic ketones were also investigated under the same TH conditions (Scheme 12). Acetophenone and its derivatives bearing electron-withdrawing (nitro, chloro, bromo) and electron-donating groups (methyl, amino) were efficiently reduced to the corresponding alcohols (206–211) in excellent yields. Alicyclic ketones such as cyclohexanone and cycloheptanone were also smoothly converted to alcohols (213–214). In addition, benzophenone and substituted benzophenones were reduced with high efficiency (215–219). Biologically relevant substrates, including furfural, nicotinaldehyde, vanillin, and isovanillin, were successfully transformed into their corresponding alcohols (220–223). Furthermore, chalcone and 3,6-dihydroxyflavone were effectively reduced under the same conditions, affording the desired products in excellent yields (224–225). Overall, catalyst 184 demonstrated superior catalytic performance across multiple parameters, including yield, selectivity, energy efficiency, atom economy, and reaction mass efficiency, as evaluated using the CHEM21 green metrics toolkit, outperforming other catalysts in this study.

**Scheme 13:**
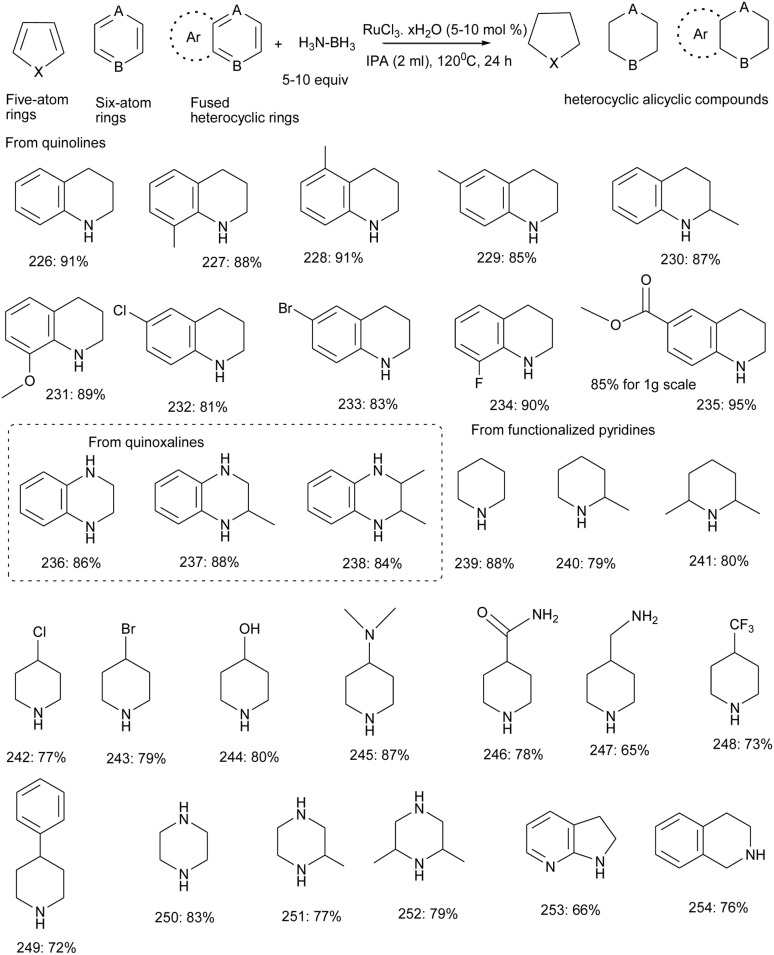
Substrate scope for Ru-catalyzed transfer hydrogenation of heteroarene with H_3_N–BH_3_. Reproduced from ref. 97 with permission from the American Chemical Society. Copyright 2024.

**Scheme 14:**
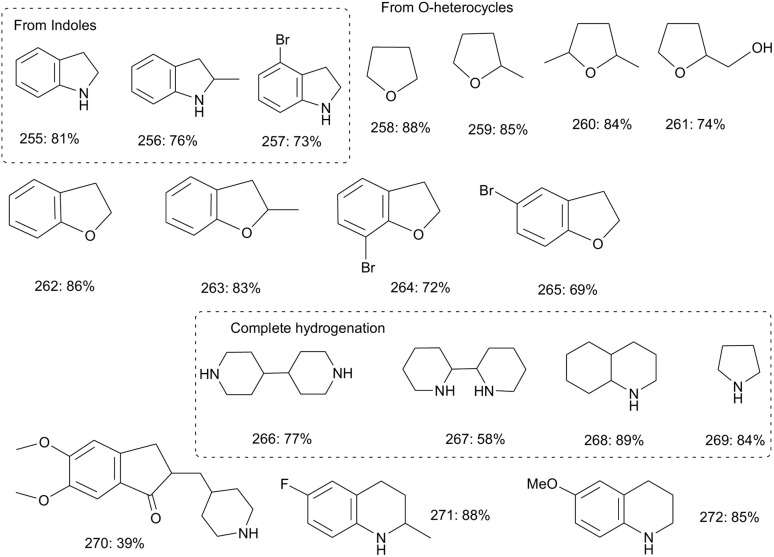
Substrate scope for Ru-catalyzed transfer hydrogenation of heteroarene with H_3_N–BH_3_.

K. Natte and co-workers^97^ (2024) reported a ligand-free TH protocol for N- and O-heteroarenes using a commercially available catalyst, RuCl_3_·*x*H_2_O, with ammonia borane (H_3_N–BH_3_) as the hydrogen source (Scheme 13). The reaction was initially optimized using quinoline as the model substrate (0.5 mmol scale), 5 mol% RuCl_3_·*x*H_2_O, 5 equivalents of H_3_N–BH_3_, and isopropanol (2 mL) at 120 °C for 24 hours. Under these conditions, 1,2,3,4-tetrahydroquinoline (226) was obtained in 91% yield. Reducing the amount of the hydrogen source significantly lowered the efficiency, with yields dropping to 79% (4 equivalents) and 38% (2 equivalents), highlighting the crucial role of H_3_N–BH_3_ in the reduction process. Other ruthenium catalysts, such as [Ru(COD)Cl_2_]_*n*_, [RuCl_2_(*p*-cymene)]_2_, and RuCl_2_(PPh_3_)_4_, yielded moderate results, with yields of 57% to 72%. A variety of solvents, including THF, 1,4-dioxane, and *tert*-butyl alcohol, were tested, achieving yields of 83% to 87%. However, reducing the reaction temperature to 100 °C resulted in decreased conversions. The optimized system exhibited a broad substrate scope. Various N-heterocycles, including quinolines, quinoxalines, pyridines, pyrazines, indoles, benzofurans, and substituted tetrahydroquinolines (226–230), as well as 8-methoxy-1,2,3,4-tetrahydroquinoline (231), were efficiently reduced to the corresponding saturated products in excellent yields (85–91%). Functionalized quinolines bearing electron-withdrawing substituents such as chloro, bromo, fluoro, and ester groups at the 6-position were smoothly hydrogenated, affording products in 81–95% yield (232–235). Quinoxaline derivatives were also efficiently reduced to the corresponding dihydro/fully hydrogenated products (236–238) in 84–88% yield. Similarly, pyridine and methyl-substituted pyridines underwent successful hydrogenation, giving piperidine derivatives in 79–88% yield (239–241). Importantly, a wide range of functional groups on the pyridine ring, including chloro, bromo, hydroxyl, dimethylamino, and carboxamide substituents, were well tolerated, furnishing the corresponding piperidines (242–246) in 77–87% yield. More challenging substrates such as 4-(aminomethyl) pyridine and 4-(trifluoromethyl) pyridine were reduced to the corresponding products in 65% and 73% yield, respectively (247, 248). Notably, 4-phenylpyridine was selectively hydrogenated at the pyridine ring while leaving the phenyl group intact, giving 249 in 72% yield. Oxygen-containing heteroarenes were also efficiently reduced. Pyrazine derivatives afforded piperazine products in 77–83% yield (250–252), while 7-azaindole and isoquinoline gave hydrogenated products in 66% (253) and 76% (254), respectively. Indole derivatives were reduced smoothly to the corresponding saturated products in 73–81% yield (Scheme 14, 255–257). Oxygen heterocycles such as 2-methylfuran, 2,5-dimethylfuran, and furfuryl alcohol were converted into the corresponding tetrahydrofuran derivatives in 74–88% yield (258–261). Benzofuran also underwent efficient hydrogenation to give 262 in 86% yield. Substituted furans bearing methyl and bromo groups were similarly well tolerated (263–265), providing high yields. Additionally, bipyridine systems (4,4′- and 2,2′-bipyridine) and quinoline derivatives (266–268) underwent complete hydrogenation to their saturated products. Pyrrole was reduced to the corresponding product in 84% yield (269). The methodology was further applied to pharmaceutically relevant molecules, including the synthesis of 5,6-dimethoxy-2-(piperidin-4-ylmethyl)-2,3-dihydro-1*H*-inden-1-one (270), a key intermediate for donepezil synthesis. Furthermore, flumequine was accessed from 6-fluoro-2-methylquinoline *via* selective hydrogenation, and 6-methoxyquinoline was converted to the corresponding tetrahydroquinoline derivative (271, 272), which served as an intermediate in tubulin inhibitor synthesis. In summary, this ligand-free ruthenium-catalyzed system highlighted broad utility, high functional group tolerance, and practical application for the TH of diverse heteroaromatic substrates.

**Scheme 15:**
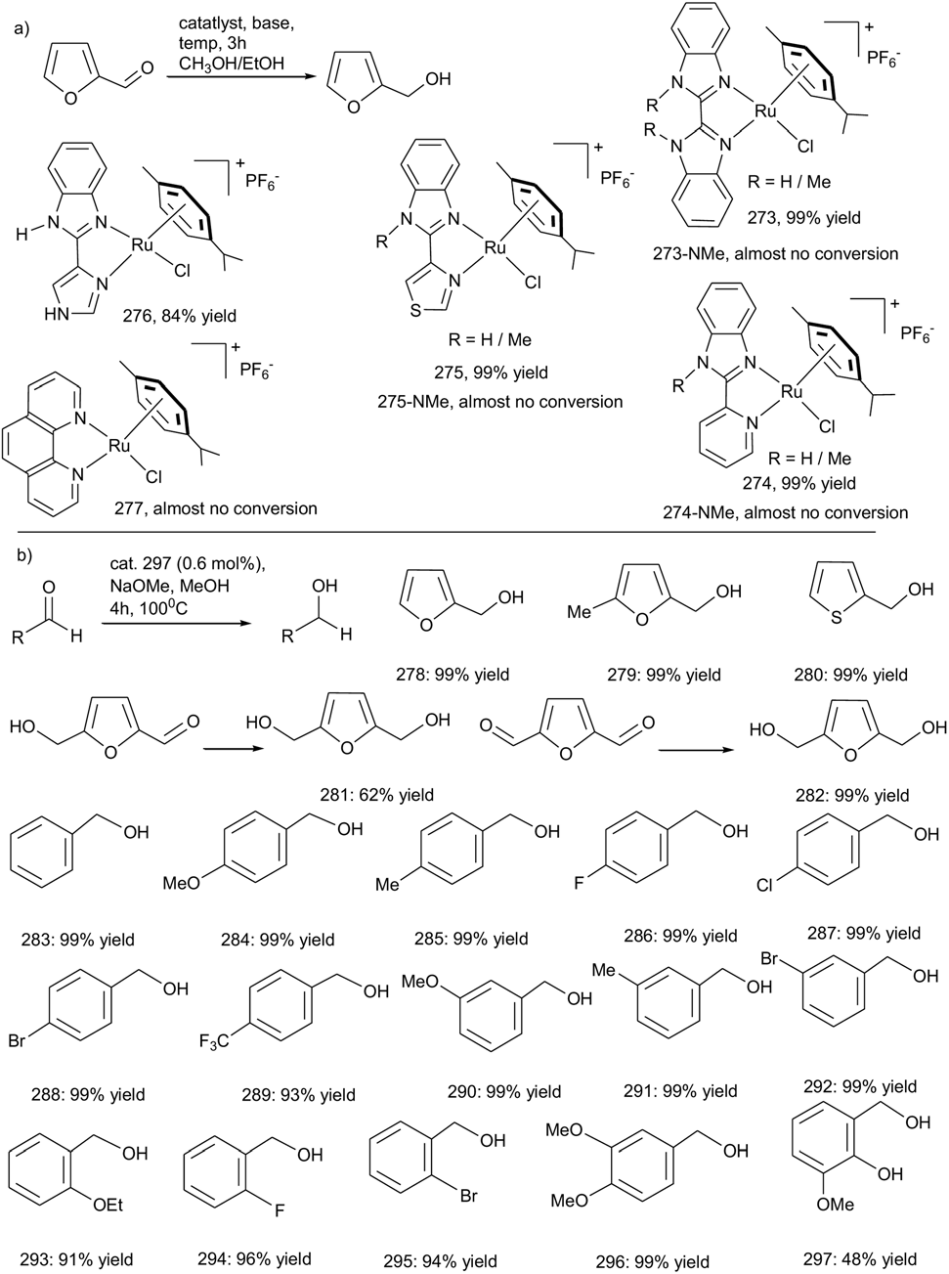
(a) Catalyst used in TH reaction. (b) TH of furanic aldehydyes and benzaldehyde derivatives. Reproduced from ref. 98 with permission from the American Chemical Society. Copyright 2024.

**Scheme 16:**
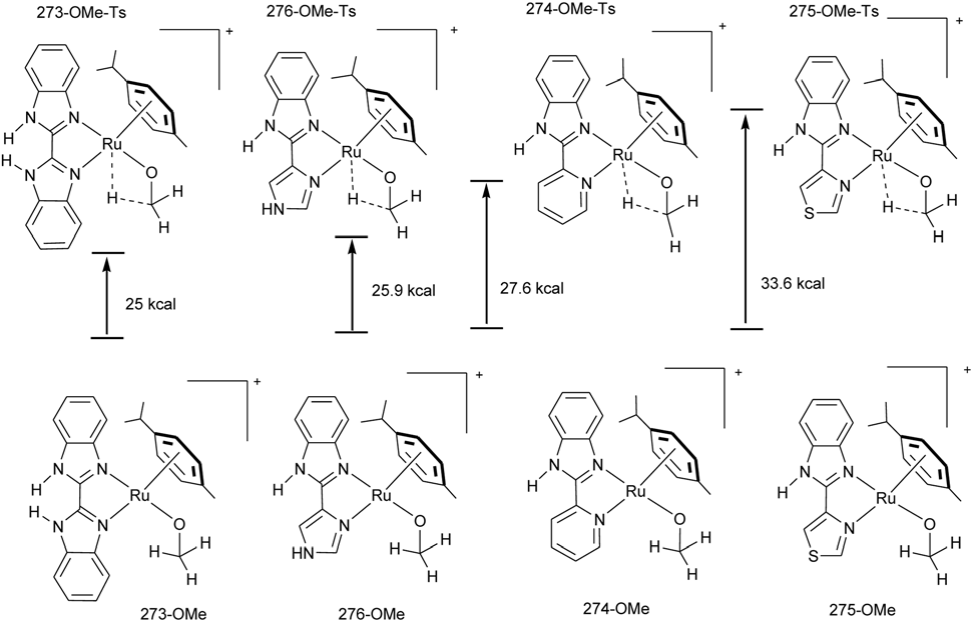
Calculated activation barrier between methoxide intermediate and their corresponding transition states involved for dehydrogenation.

Sanjay Pratihar and co-workers^98^ (2024) investigated the TH of furanic aldehydes and benzaldehyde derivatives using air-stable imidazole-based ruthenium catalysts that operate *via* metal–ligand cooperativity. The study focused on catalyst 273, a Ru(ii) complex based on 2,2′-bibenzo[*d*]imidazole, along with related ligand frameworks. Under optimized conditions, furfural was efficiently reduced to furfuryl alcohol using catalyst 273 in the presence of sodium methoxide (NaOMe) in methanol, affording the product within 3 hours. Solvent screening showed that methanol and ethanol were superior to isopropanol in terms of reactivity and efficiency. Additional catalysts, including 2-(pyridin-2-yl)-1*H*-benzo[*d*]imidazole (274), 2-(4-thiazolyl) benzimidazole (275), 2-(1H-imidazole-5-yl)-1*H*-benzo[*d*]imidazole (276), and 1,10-phenanthroline (277), were also evaluated (Scheme 15). While catalysts 274 and 275 delivered yields comparable to 273, catalyst 276 afforded an isolated yield of 82%. In contrast, catalyst 277 showed comparatively lower activity. To probe the role of the N–H functionality, *N*-methylated derivatives of catalysts 273–275 were tested, but all were completely inactive, clearly indicating that the N–H proton is essential for activating the catalysis process and metal–ligand cooperative proton transfer. Under optimized conditions, the 273 catalyst was used to reduce substrates such as furfural, hydroxymethyl furfural, 5-methyl furfural, and furan-2,5-dicarbaldehyde, thiophene-2-carbaldehyde (Scheme 15). These substrates were smoothly hydrogenated, resulting in the corresponding alcohol with good to excellent yields (278–282). Furthermore, this methodology was utilized for benzaldehyde derivatives. *Para*-substituted benzaldehyde bearing both electron-donating substituents (OMe, Me) and electron-withdrawing groups (F, Cl, Br, and CF_3_) were well tolerated, providing corresponding alcohol products with excellent yields (286–289). Moreover, the effects of substituents at the *ortho* and *meta* positions of benzaldehyde derivatives were examined; both electron-donating (OMe, Me, OEt) and electron-withdrawing groups (F, Br), all yielding corresponding alcohols with satisfactory yields. The mechanistic details were investigated through spectroscopic methods, *in situ* monitoring, labelling, and DFT studies. Kinetic analysis provided rate constants (*k*) for 273, 274, 275, and 276, which were 1.4 ± 0.1, 1.3 ± 0.2, 0.8 ± 0.4, and 1.3 ± 0.1, respectively, indicating that 273 exhibits significantly higher reactivity. The NMR experiment suggests that the N–H protons of 273 and 276 were more acidic and facilitated the formation of the metal hydride intermediate due to the electron-deficient Ru centre. The calculated activation energies of the Ru-alkoxide and the transition state leading to the formation of ruthenium hydride intermediate varied, with the order of activation energy being 273 < 276 < 274 < 275 (Scheme 16). Styrene, diphenylacetylene, *cis*- and *trans*-stilbene, phenylacetylene, benzonitrile, benzoic acid, methyl benzoate, benzamide, β-nitrostyrene, and imidazole-4-carboxylic aldehydes were tested for the TH reaction but were not reduced by these catalysts. Substrates containing nitro (NO_2_), nitrile (CN), and amino (NH_2_) groups in benzaldehyde were also inactive in this catalytic reaction. At a 2.0 mmol scale of furfural, catalyst 273 afforded a 90% isolated yield, while catalysts 274 and 275 gave 79%. Increasing the substrate amount to 5.0 mmol resulted in yields of 88% for 273, compared to only 42% and 40% for 274 and 275, respectively. Even at a 10.0 mmol scale, catalyst 273 maintained good efficiency, delivering a 71% isolated yield, confirming its superior catalytic performance under both standard and scale-up conditions.

**Scheme 17:**
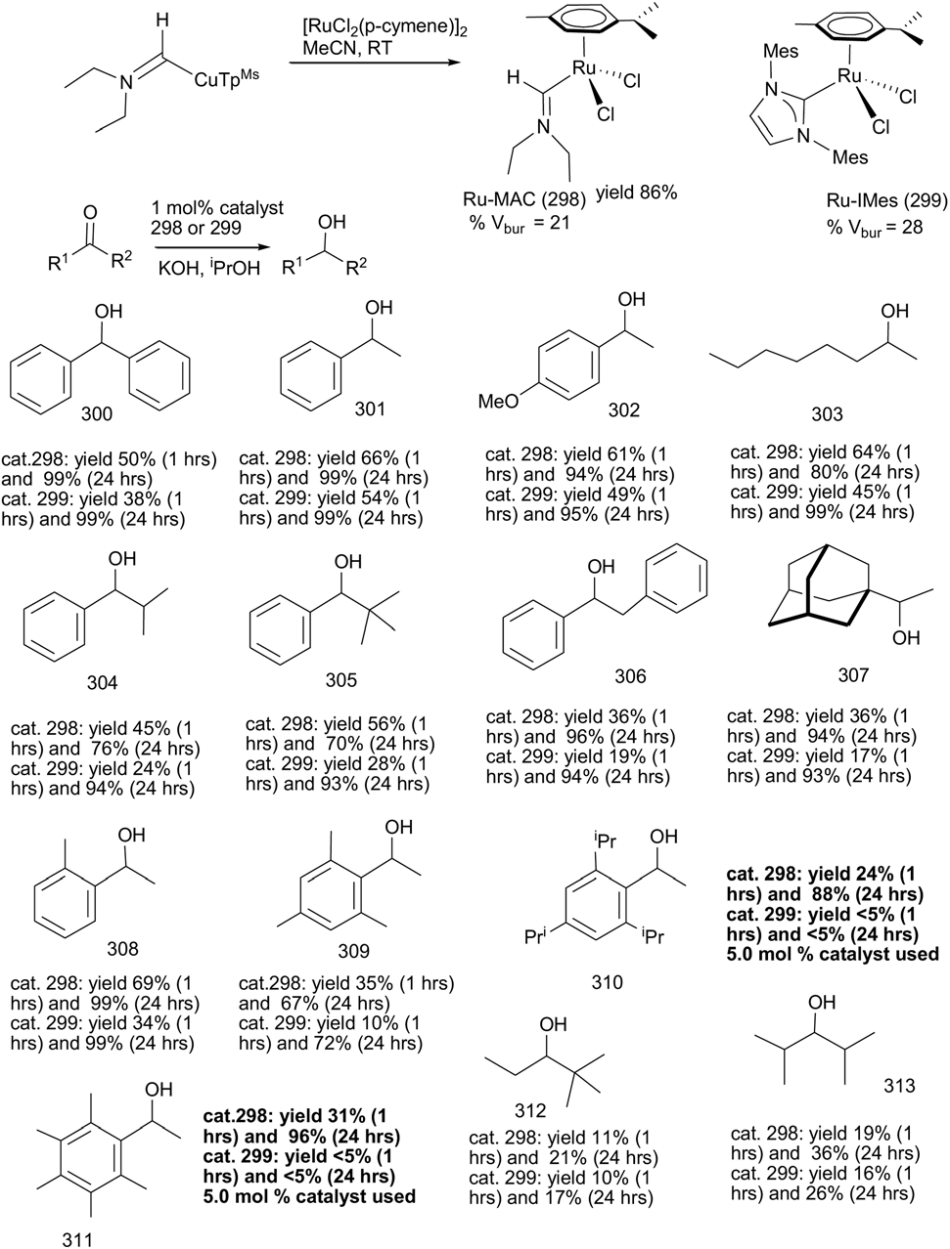
Synthesis of Ru–MAC and scope of the transfer hydrogenation reaction with complex Ru–MAC and Ru–IMes. Reproduced from ref. 99 with permission from the American Chemical Society. Copyright 2024.

Martin Albrecht and co-workers^99^ (2024) reported the successful synthesis of a mono-substituted amino carbene (MAC) ligand and its corresponding ruthenium complex, Ru–MAC (298), which is characterized by a relatively low buried volume. The complex was prepared by reacting TpMsCu–MAC (where TpMs = hydrotris(3-mesitylpyrazolyl) borate and MAC = C(H)NEt_2_) with [RuCl_2_(*p*-cymene)]_2_ in acetonitrile, affording the desired product (Scheme 17). In the ^1^H NMR spectrum, a resonance at 11.27 ppm is attributed to the proton attached to the carbene carbon. In the ^13^C NMR spectrum, a signal at 244.1 ppm confirms coordination of the carbene carbon to the ruthenium center. Single-crystal X-ray diffraction analysis revealed a three-legged piano-stool geometry, with a Ru–C(carbene) bond distance of 2.008(2) Å, consistent with related carbene complexes reported in the literature. Buried volume analysis (% Vbur) suggests that IMes is bulkier (Ru–IMes, 28.0%) than MAC of Ru–MAC (21.0%), indicating more steric encumbrance around the Ru–IMes. The electronic properties of the two compounds were examined using cyclic voltammetry (CV) analysis. The oxidation potential of Ru–MAC (Ru^ii/iii^) was observed at *E*_1/2_ = +0.59 V, while that of Ru–IMes (Ru^ii/iii^) was at *E*_1/2_ = +0.51 V, which suggests that both compounds have comparable donor properties. The catalytic activity was evaluated in the presence of base (KOH) and isopropanol (^*i*^PrOH) as both the solvent and hydrogen source at 90 °C, with bulky ketone benzophenone yielding the corresponding alcohol product. The Ru–IMes catalyst required 24 hours to complete the reaction, while Ru–MAC achieved completion in just 8 hours. After 1 hour, Ru–MAC exhibited a turnover frequency (TOF_1h_) of 50 h^−1^ compared to 39 h^−1^ for Ru–IMes, corresponding to an acceleration factor (AF) of 1.3. This advantage increased slightly at 80 °C (AF ≈ 1.4). With less hindered substrates such as acetophenone, Ru–MAC again showed higher activity (TOF_1h_ = 66 h^−1^) than Ru–IMes (54 h^−1^). The effect became more pronounced with increasing steric bulk. For *tert*-butyl phenyl ketone, Ru–IMes achieved only 28% conversion after 1 hour, whereas Ru–MAC reached 56%, giving an AF of 2.0. Similar acceleration factors (∼2.0) were observed for phenyl benzyl ketone and adamantyl methyl ketone. Mesityl methyl ketone showed an even higher AF of 3.5 (TOF_1h_ = 35 h^−1^ for Ru–MAC *vs.* 10 h^−1^ for Ru–IMes). In cases involving highly sterically hindered ketones, Ru–MAC achieved full conversion, whereas Ru–IMes showed no reactivity (310, 311). In contrast, both catalysts performed poorly with simple aliphatic ketones.
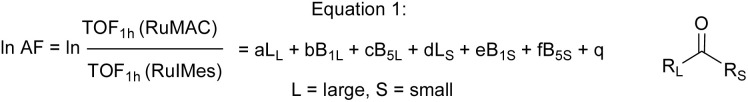


To rationalize these observations, multivariate regression analysis was performed correlating the acceleration factor (AF) with steric parameters of the substrates. A strong agreement was found between experimental and calculated AF values (eqn (1)). For small substituents, steric contributions were negligible, whereas bulky groups showed significant effects, particularly when *ortho* substitution restricted rotation around the carbonyl–R bond. A plot of the distortion angle *θ*, with limited rotation of the carbonyl–R bond of substrates against the experimental rate acceleration, shows a linear correlation. This demonstrates that Ru–MAC possesses an exceptional ability to catalyse reactions in a sterically hindered environment.

**Scheme 18:**
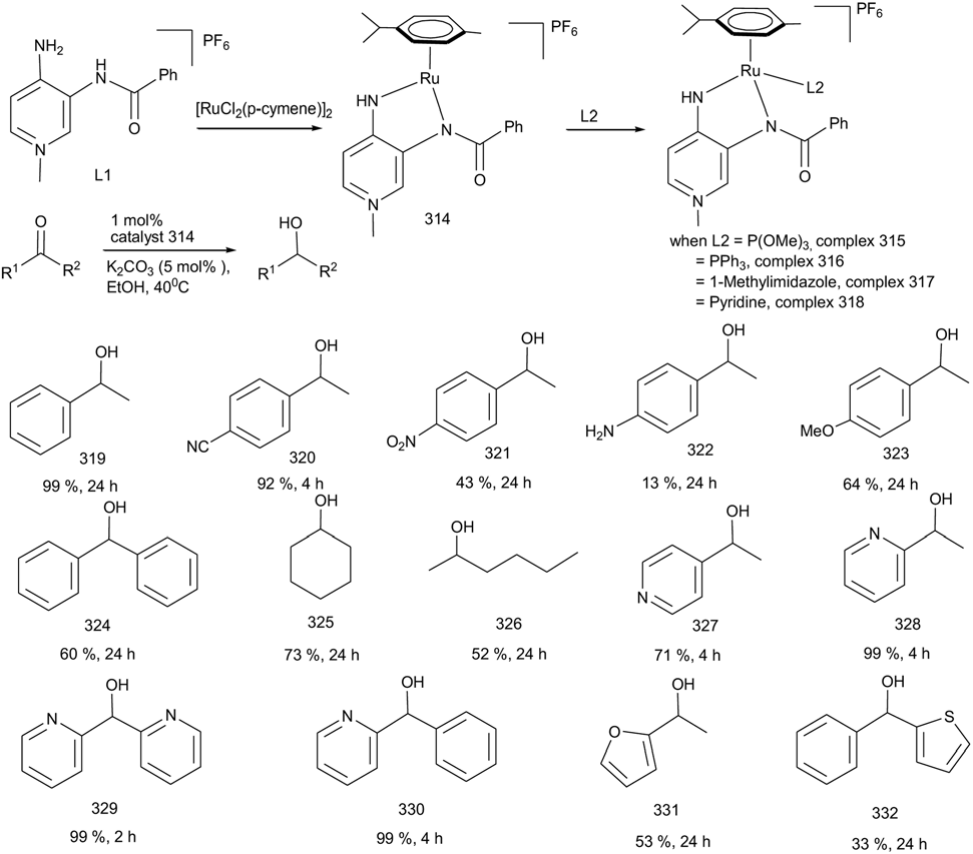
Synthesis of Ru-complexes and scope of the transfer hydrogenation reaction with complex 314 using EtOH. Reproduced from ref. 100 with permission from the American Chemical Society. Copyright 2024.

Martin Albrecht *et al.*^100^ (year 2024) showed the acatalytic activity of a new ruthenium complex, [Ru(cym) (N, N′)] ^+^, (314), containing an amide-functionalized pyridylidene amide (PYA). This complex was synthesized by the reaction of [RuCl_2_(*p*-cymene)]_2_, sodium acetate (NaOAc), and pyridinium hexafluorophosphate ligand in dichloromethane solvent, affording an air stable, red colour compound with a yield of 66% (Scheme 18). In the proton NMR spectra, the ligand –NH_2_ appears at *δ* 6.59 ppm, but upon complexation, it shifts to 9.98 ppm. Single-crystal X-ray diffraction analysis revealed a two-legged piano-stool geometry for complex 314. The reactivity of 314 was explored with various donor ligands, including nitrogen donors such as pyridine and *N*-methylimidazole (NMI), and phosphorus donors such as triphenylphosphine (PPh_3_) and trimethyl phosphite [P(OMe)_3_]. Coordination occurred selectively with N- and P-donor ligands, forming complexes 315–318. This coordination was accompanied by a distinct colour change from deep red to yellow/orange and a UV-visible absorption shift from 409 nm (338) to approximately 350 nm (315–318). In addition, all pyridylidene proton signals exhibited an upfield shift relative to the parent complex. The structure of complex 316 was confirmed by single-crystal X-ray analysis, revealing a three-legged piano-stool geometry. Catalytic activity of complex 314 was evaluated in the TH of acetophenone using isopropanol as solvent, affording phenethyl alcohol in 99% yield within 4 hours. In contrast, reactions conducted in ethanol and methanol gave significantly lower yields (66% and 40%, respectively) even after 24 hours at 80 °C. A minor side product, 1-phenylbutanone, was also observed. Temperature screening showed that higher temperatures decreased the yield, while lowering the temperature to 40 °C in the presence of K_2_CO_3_ improved the reaction efficiency, giving quantitative conversion. Alternative bases such as Cs_2_CO_3_ and Na_2_CO_3_, resulted in lower yields. Under optimized conditions (1 mol% catalyst, 5 mol% K_2_CO_3_, ethanol, 40 °C, 24 h), a series of catalysts 314–318 was tested. Catalyst 314 exhibited the highest activity, delivering 99% yield, with the overall activity trend observed as 314 > 318 > 317 > 316 > 315. A broad substrate scope was investigated. *Para*-cyano-substituted acetophenone was rapidly reduced to the corresponding alcohol in 92% yield within 4 hours (320). In contrast, electron-withdrawing nitro and electron-donating amino substituents resulted in reduced yields of 43% and 13%, respectively (321, 322), while a methoxy-substituted substrate gave a moderate yield of 64% (323). Diphenyl ketone afforded the corresponding alcohol in 60% yield (324). Both cyclic and acyclic dialkyl ketones were also compatible, giving cyclohexanol (73%, 325) and 2-hexanol (52%, 326), respectively. Notably, pyridyl- containing ketones showed excellent reactivity, affording hydrogenated products in high to excellent yields (71–99%) within 2–4 hours. Mono- and dipyridyl ketones were reduced particularly efficiently, giving up to 99% yield in short reaction times (327–330). In contrast, heteroaryl ketones such as furyl and thiophene derivatives gave lower yields of 53% and 33%, respectively, after 24 hours (331, 332).

**Scheme 19:**
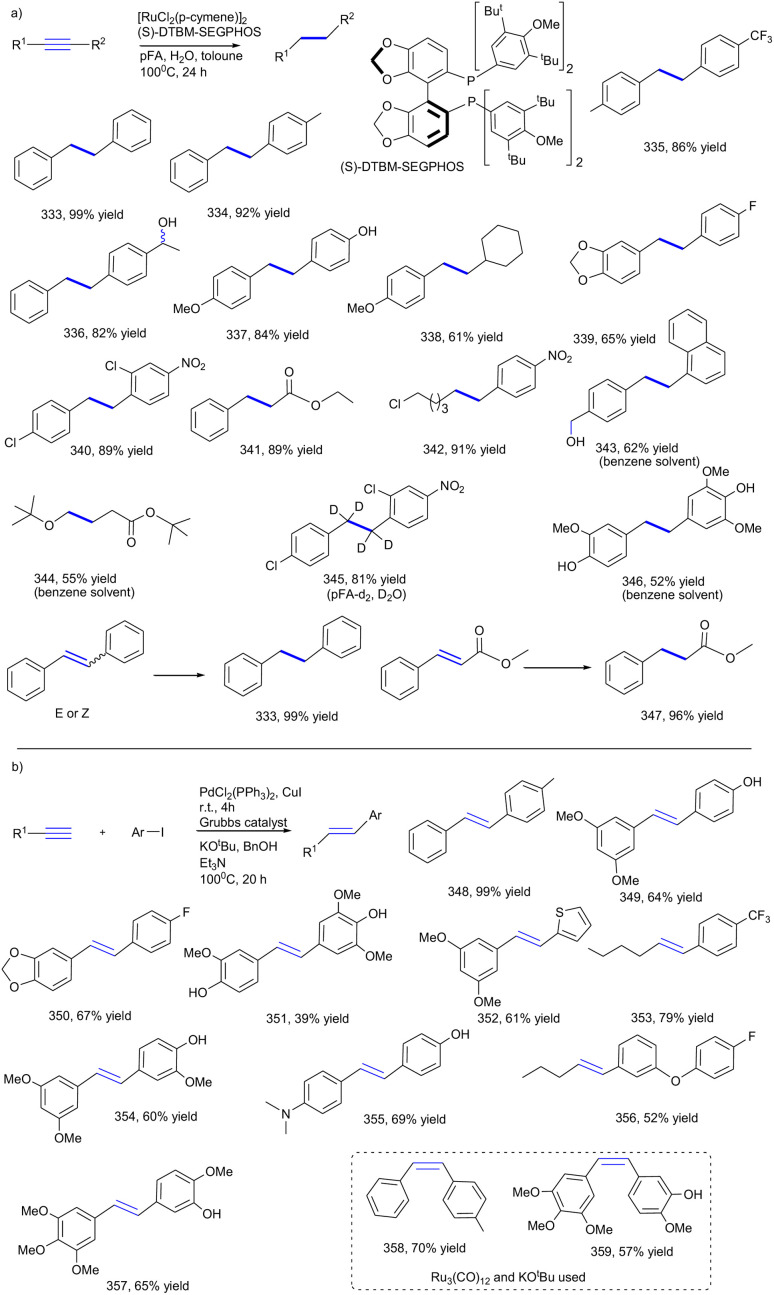
(a) Substrate scope of the developed full hydrogenation of internal alkynes. (b) Substrate scope of the sequential Sonogashira cross-coupling-hydrogenation reaction. Reproduced from ref. 101 with permission from the American Chemical Society. Copyright 2025.

In 2025, Martin Gazvoda^101^ and co-workers demonstrated an efficient ruthenium-catalyzed complete hydrogenation of alkynes using diphosphine ligands, with paraformaldehyde and water serving as the hydrogen source. A range of aryl-substituted diphosphine ligands was screened, including BINAP, (*S*)-SEGPHOS, and (*S*)-DTBM-SEGPHOS. Among these, (*S*)-DTBM-SEGPHOS showed the highest catalytic efficiency, delivering superior yields. Under the optimized conditions, internal alkynes were smoothly converted into the corresponding alkanes in moderate to excellent yields (52–99%) (Scheme 19). Substrates bearing electron-withdrawing substituents generally gave higher yields (340–342) compared to those containing electron-donating groups (338, 346), indicating a clear electronic effect on reactivity. Aldehyde-containing substrates were selectively reduced to the corresponding alcohols (336, 343), whereas nitro and ester functionalities remained intact, demonstrating good chemoselectivity (340–342, 344). This methodology was also successfully applied to the synthesis of the natural product moscatilin (346). In addition, a tandem catalytic sequence combining Pd/Cu-catalyzed Sonogashira coupling with a subsequent ruthenium-catalyzed transformation (using a Grubbs-type catalyst) enabled the formation of *E*-alkene products in yields ranging from 39% to 99%. When Ru_3_(CO)_12_ and a reduced amount of KO^*t*^Bu were employed instead, *Z*-selective products were obtained, allowing access to stilbene derivatives. The protocol was further extended to the synthesis of biologically important molecules, including pterostilbene (349) and isorhapontigenin (354), obtained in 64% and 60% yields, respectively. Importantly, both (*E*)-combretastatin A4 (357) and (*Z*)-combretastatin A4 (359), compounds of significant medicinal relevance, were also successfully synthesized using this strategy.

## Chiral transfer hydrogenation

**Scheme 20:**
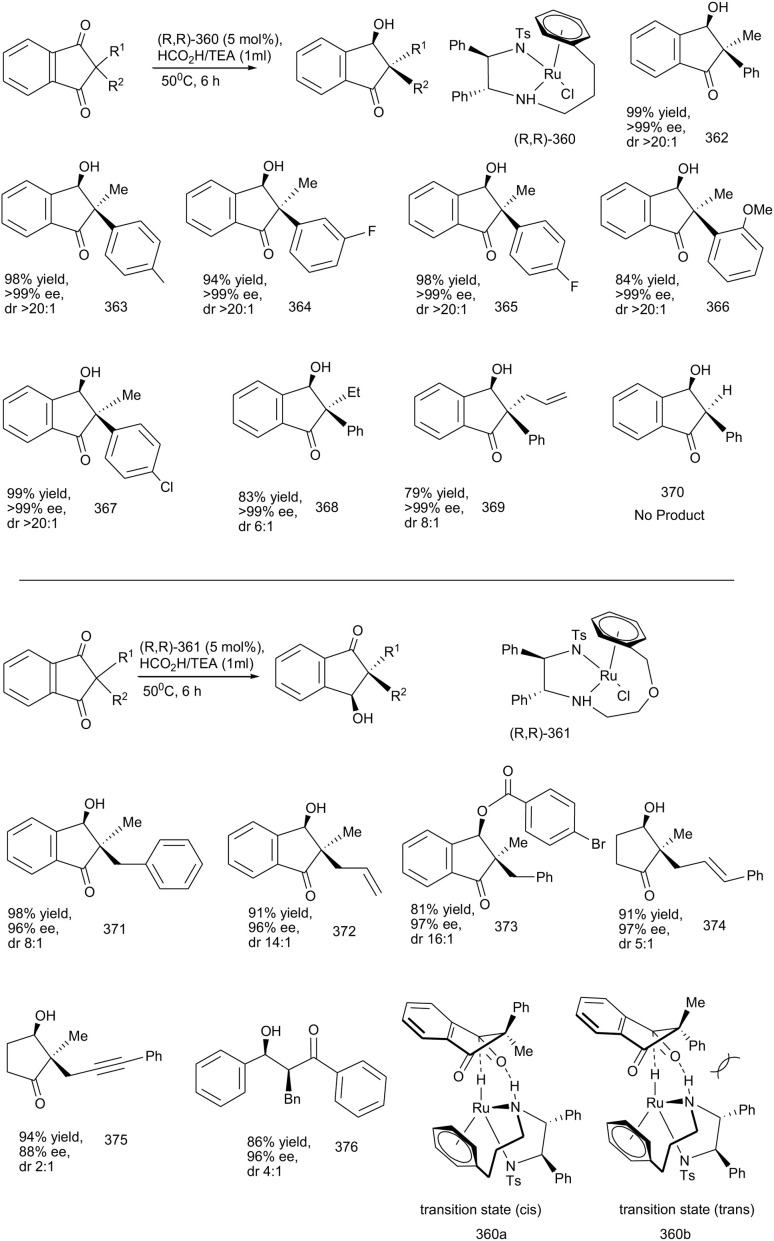
Substrate scope of ruthenium-catalyzed transfer hydrogenative desymmetrization reaction of 1,3-cyclopentanediones. Reproduced from ref. 102 with permission from Wiley-VCH. Copyright 2020.

The Yong-Gui Zhou group^102^ (2020) reported a highly efficient ruthenium-catalyzed transfer hydrogenative desymmetrization of 1,3-cyclopentanediones, providing access to chiral *cis*-β-hydroxy ketones with excellent stereocontrol (up to 99% ee and dr > 20 : 1) (Scheme 20). Using the chiral Ru(ii)–TsDPEN catalyst (*R*,*R*)-360 and a formic acid/triethylamine (FA/TEA) azeotrope as the hydrogen source, a broad range of substrates was investigated, particularly those bearing an aryl group at the *R*^2^ position and a methyl group at *R*^1^. Under these conditions, the corresponding products (362–367) were obtained in excellent yields (84–99%) with uniformly high enantioselectivities (>99% ee) and good diastereoselectivities (dr > 20 : 1). Importantly, both electron-donating and electron-withdrawing substituents on the aromatic ring were well tolerated without significant loss of stereocontrol. When the R^1^ substituent was changed from methyl to ethyl or allyl, the reaction still proceeded with excellent enantiomeric excess (ee) (99%); however, the dr decreased to 6 : 1 and 8 : 1, respectively (368 and 369). In contrast, the desymmetrization reaction was not observed when R_1_ was hydrogen (370), indicating the importance of steric bias at this position for effective stereodiscrimination. The scope was further extended to dialkyl-substituted 1,3-cyclopentanediones substrate yielded a lower dr of 2 : 1 (compound 371). However, changing the catalyst (*R*,*R*)-361 improved the results, provided the product in 98% yield with 96% ee and 8 : 1 dr. Switching *R*^2^ to allyl under these reaction conditions resulted in a yield of 91% with 96% ee, and 14 : 1 dr. The transfer hydrogenative desymmetrization of substrates containing cinnamyl and propargyl groups also demonstrated good ee, although the dr were lower (compounds 374 and 375). Additionally, linear diketones smoothly reduced with very good ee, where one ketone group remained intact without reduction (compound 376). The gram-scale reactions were successful without any loss of reactivity or enantioselectivity. Furthermore, the bioactive molecule (+)-estrone was synthesised using this transfer hydrogenative desymmetrization process. The transition states of asymmetric induction are illustrated in 360a (*cis*) and 360b (*trans*), with the *cis* product being favoured highlighting steric over the *trans* product, due to more effective steric alignment and stabilizing interaction between the ligand and the substrate.

**Scheme 21:**
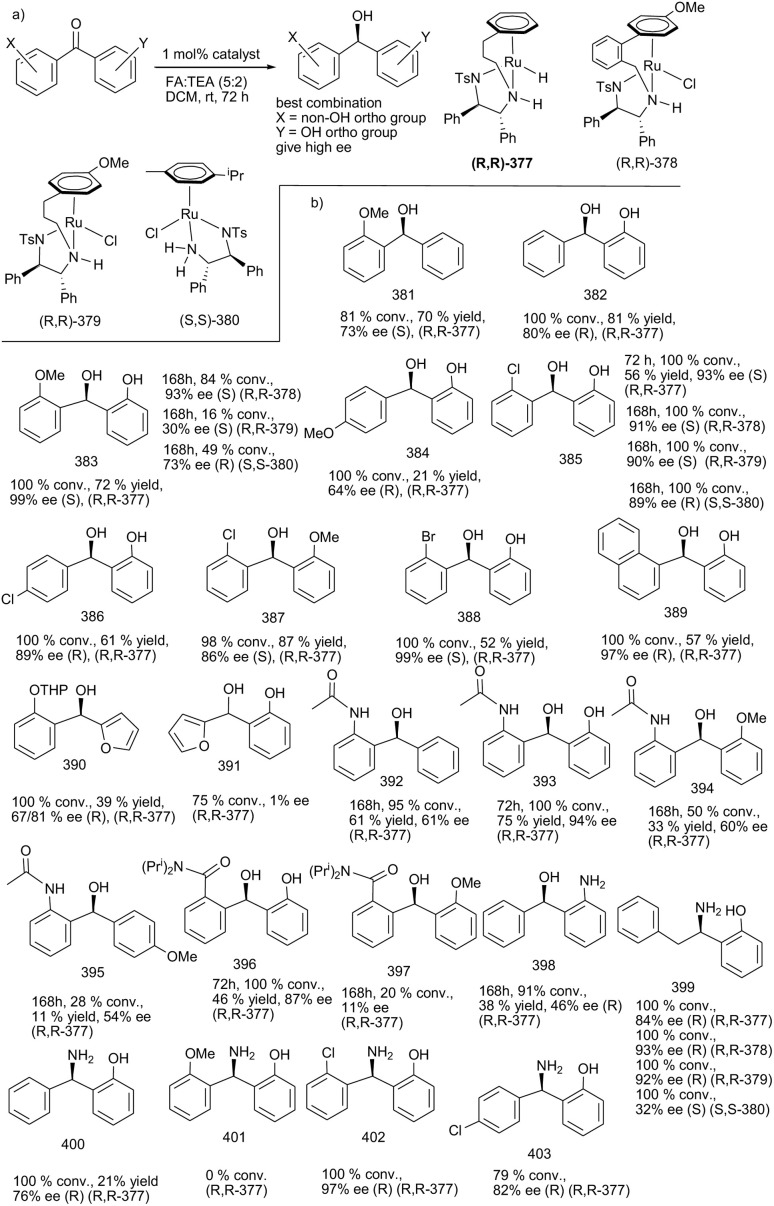
(a) ATH catalysts employed in this study. (b) General procedure for ATH of *o*-hydroxyphenyl ketones and broader range of substrates using (*R*,*R*)-377 and the products obtained. Reproduced from ref. 103 with permission from the American Chemical Society. Copyright 2020.

Martin Wills and co-workers^103^ (2020) reported an efficient ATH of *ortho*-hydroxyphenyl ketones using a Ru(ii) C3-tethered catalyst system. The study demonstrated that both hydrogen-bonding interactions and steric effects at the *ortho* position play a crucial role in controlling enantioselectivity. In the case of *ortho*-hydroxyacetophenone and *ortho*-methoxyacetophenone, the corresponding alcohols were obtained with high ee (>98%) and moderate to good ee (70–89%), respectively. The enhanced stereocontrol observed for the hydroxyl-substituted substrate was attributed to intramolecular hydrogen bonding between the *ortho*–OH group and the carbonyl functionality, which helps to pre-organize the substrate in a well-defined conformation. In contrast, the *ortho*-methoxy group lacks hydrogen-bond donating ability and introduces steric hindrance, resulting in reduced enantioselectivity. Using catalyst (*R*,*R*)-377, *o*-methoxyphenyl and *o*-hydroxyphenyl ketones afforded alcohols with 73.4% ee (*S* configuration, 381) and 80% ee (*R* configuration, 382), respectively (Scheme 21). Interestingly, a substrate bearing both *ortho*-OH and *ortho*-OMe substituents delivered an excellent ee (99%) with *S* configuration (383), which was confirmed by single-crystal X-ray analysis. Other catalysts (378–380) showed inferior performance in comparison.

**Scheme 22:**
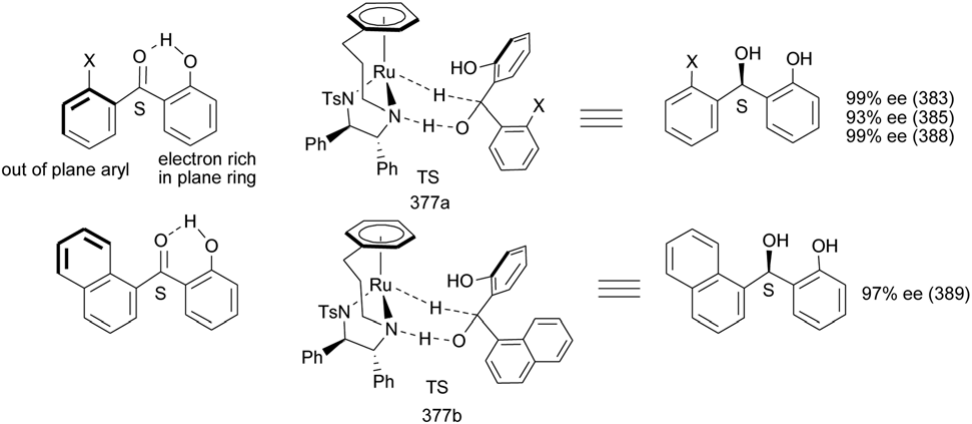
Proposed approach of the substrate to catalyst (*R*,*R*)-377 for the formation of 383, 385, 388, and 389.

Substrate scope studies revealed that electronic effects on the aromatic ring also influence stereoselectivity. For example, electron-donating methoxy substitution led to a moderate ee (64%, 384), whereas chloro-substituted substrates at *ortho* and *para* positions gave improved enantioselectivities of 93% ee (385, *S*) and 89% ee (386, *R*), respectively. Additional catalyst screening for product 385 showed consistently high ee (89–91%), further supporting the robustness of the system. A mixed-substituent substrate containing both OMe and Cl groups afforded product 387 with 86% ee (*S* configuration), as confirmed by X-ray crystallography. Steric effects were also found to be important. Bulky *ortho*-bromo and naphthyl-substituted substrates provided excellent ee, yielding products 388 and 389 with 99% and 97.2% ee, respectively. A furan-containing substrate with a bulky group gave alcohol products with ee values ranging from 67% to 81% (390); notably, for an *ortho*-hydroxyl aryl ring-containing substrate, only a 1% ee product was obtained (391). This indicates that the hydroxyl group donates an electron to the phenyl ring, enhancing the electron density and forming a hydrogen bond between the hydroxyl hydrogen and the ketone (Scheme 22). In cases with bulky substituents at the *ortho* position, the ring's distortion out of the plane provides additional stability to the transition state. An amide-containing phenyl substrate showed good catalytic activity, yielding a product with 61% ee (392). Moreover, the amide-containing *ortho*-hydroxyphenyl substrate furnished a product with 94% ee (393). The combination of amide and methoxy in a ketone showed low catalytic activity, with only 33% yield and 60% ee (394) and 11% yield with 54% ee (395). Additionally, a bulkier amide-containing ketone, a yield of 45% with 87% ee (396), was achieved; however, if the substrate contained both -OMe and amide, it only resulted in 20% conversion and 11% ee (397), suggesting unfavourable steric and electronic interaction. An *ortho*-aminophenyl ketone also underwent reduction, but with lower efficiency (38% yield, 46% ee, 398), showing that –NH_2_ is less effective than –OH in directing stereocontrol. The catalyst system was also applied to imine reduction. Benzyl-substituted imines were reduced smoothly to the corresponding amines with full conversion. Catalyst (*R*,*R*)-377 gave 84% ee (*R* configuration), while catalysts 378 and 379 provided slightly higher ee (93% and 92%), and catalyst 380 performed poorly (32% ee). Notably, only catalyst 377 successfully converted certain imines to amines (400) with complete conversion and 76% ee, whereas others were ineffective. Highly sterically hindered imines did not undergo reduction (401), highlighting the steric limitations of the system. Chloro-substituted imines were efficiently reduced, giving products with excellent conversion and ee (402, 97%; 403, 82%). Overall, the study establishes that *ortho*-hydroxy substituents, through hydrogen bonding and structural control, enhance stereoselectivity in ATH reactions, while steric and electronic effects play vital roles to enhance both reactivity and enantio induction.

**Scheme 23:**
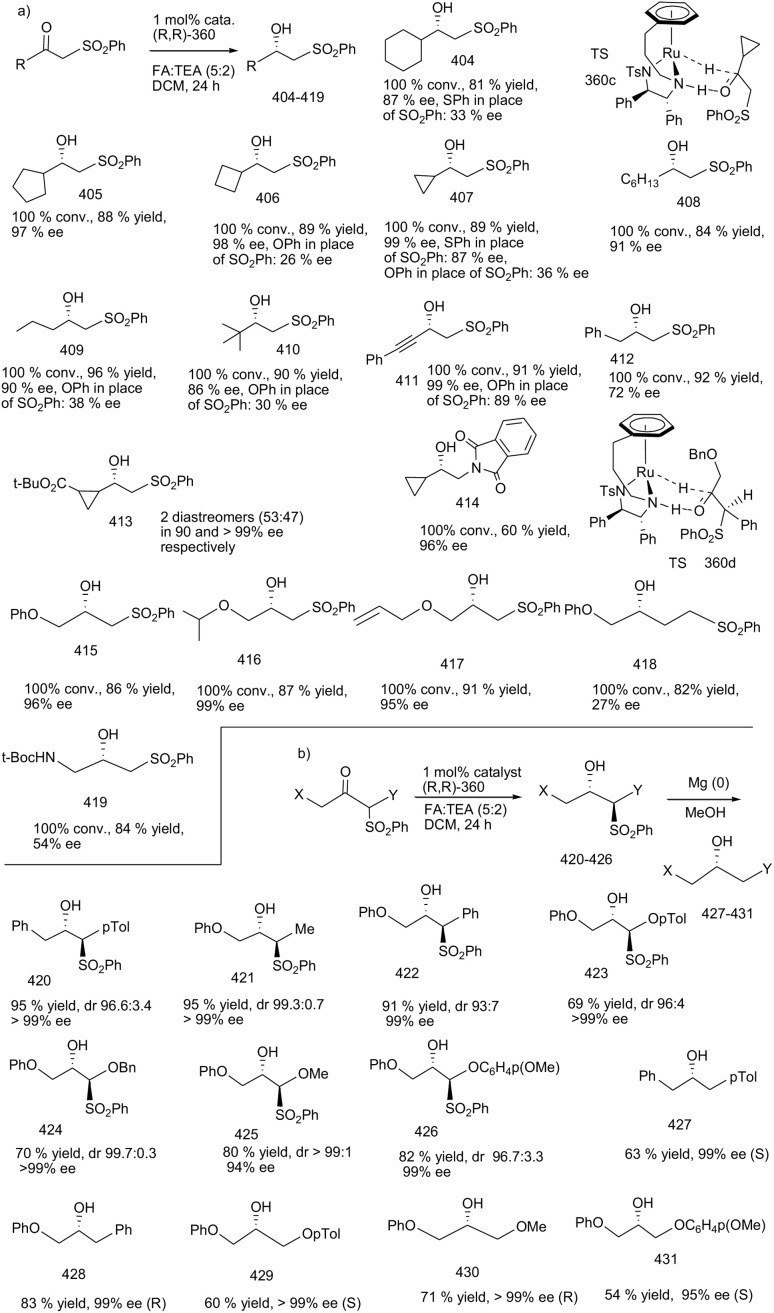
(a) ATH of α-sulfonyl, cyclopropyl-functionalised, alkoxy and amino-substituted ketones with a tethered ATH catalyst. (b) Diastereoselective ATH products with DKR and the products of sulfone reduction. Reproduced from ref. 104 with permission from Wiley-VCH. Copyright 2020.

Martin Wills *et al.*^104^ (year 2020) demonstrated the ATH of a range of α-sulfonyl ketones using a 3C-tethered catalyst, (*R*,*R*)-360 (Scheme 23a). The study provided that both steric and electronic features of the sulfonyl ketone strongly influence catalytic efficiency and enantioselectivity. A cyclohexyl-substituted sulfonyl ketone was reduced to the corresponding alcohol in 81% yield with 87% ee (404). Interestingly, a clear trend was observed with decreasing ring size: as the substituent was reduced from cyclohexyl to cyclopentyl, cyclobutyl, and cyclopropyl, both yield and enantioselectivity improved significantly. The corresponding alcohols (405–407) were obtained in 88–89% yield with excellent enantioselectivities of 97–99% ee. This trend suggests that steric bulk in the cyclic substituent can hinder optimal catalyst-substrate alignment, thereby reducing stereocontrol. A variety of alkyl-substituted sulfonyl ketones were also well tolerated. Hexyl, propyl, and *tert*-butyl derivatives afforded the corresponding alcohols (408–410) in 84–96% yield with good enantioselectivities (86–91% ee). In addition, substrates bearing unsaturated or benzylic substituents such as phenylacetylene and benzyl groups were successfully reduced, giving products (411, 412) in 91–92% yield with 99% ee and 72% ee, respectively. The stereochemical outcome is rationalized by a transition state model (TS-360c), in which the sulfone group is oriented away from the η^6^-arene of the catalyst, minimizing steric repulsion and allowing efficient hydride transfer. The catalyst also showed broad functional group tolerance. Ester-functionalized cyclopropane ketones and phthalimide-containing substrates underwent ATH smoothly. In the former case, product 413 was obtained as a near-racemic diastereomeric mixture (53 : 47 dr) but with high ee (90% and >99% for individual diastereomers), while the latter afforded product 414 in 60% yield with 96% ee. This high enantioselectivity indicates there was interaction between the η^6^-arene and the cyclopropyl group. Oxygen-functionalized sulfone-substituted ketones were also reduced to give alcohol products with good yields and ee, such as 415 (86% yield, 96% ee), 416 (87% yield, 99% ee), and 417 (91% yield, 95% ee). However, when the sulfone group was positioned further away from the ketone, the yield slightly dropped to 82%, and the enantioselectivity was significantly lower (27% ee) (418). Additionally, ^*t*^Boc-protected amine-containing sulfone substrates were tolerated in this reaction, leading to alcohol with an 84% yield and 54% ee (419). The ATH of sulfone-containing substrates was carried out using catalyst (*R*,*R*)-360, yielding alcohol products (420–426) *via* a DKR process. The yields ranged from 69% to 95%, with product 425 achieving 94% ee and the remaining cases obtaining >99% ee. The diastereoselectivity (dr) for all products was outstanding in this reduction process. This high stereocontrol is attributed to steric repulsion between bulky sulfone substituents and the catalyst framework in the key transition state (TS-360d), which directs selective hydride delivery. Finally, desulfonylation of selected products using magnesium provided secondary alcohols (427–431) with excellent ee (>99%). In contrast, direct ketone reduction without the sulfone directing group led to a dramatic loss of stereocontrol, with enantioselectivities ranging from 0% to 64%. This clearly demonstrates that the sulfone moiety plays an important role in enhancing both catalytic activity and enantioinduction, acting as a key directing group in resulting in high asymmetric control in the ATH process.

**Scheme 24:**
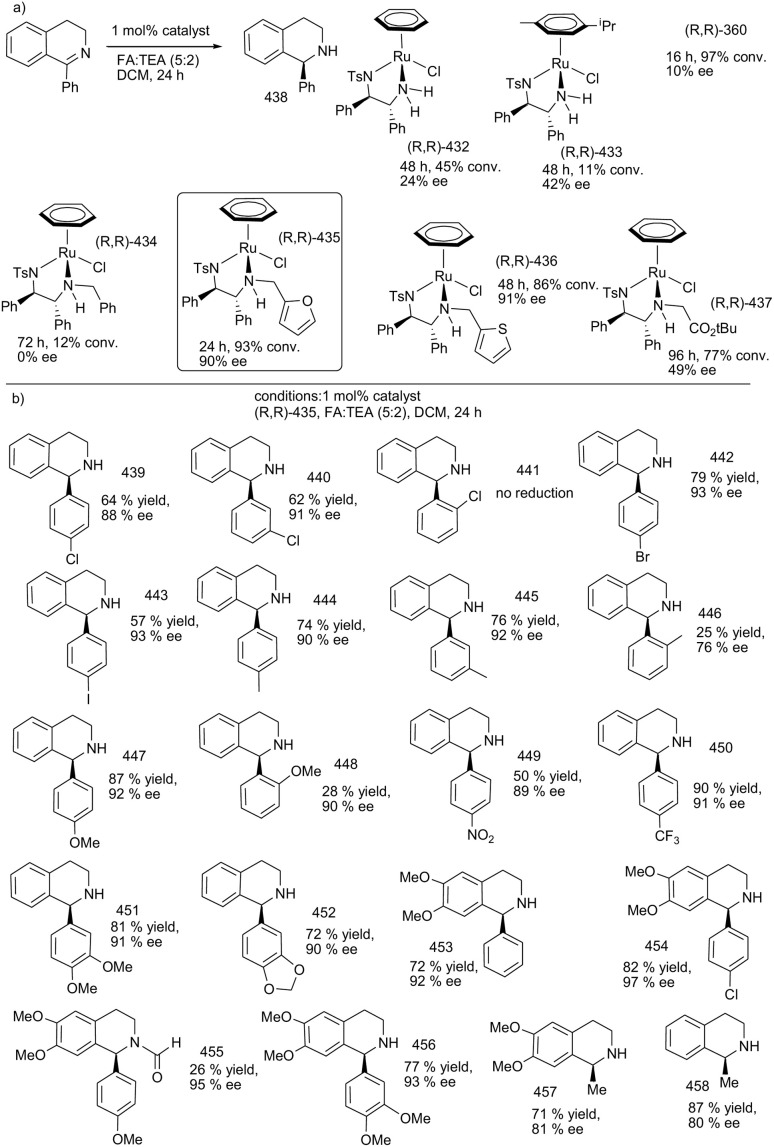
(a) Catalyst screening. (b) Products from non-electron-rich and electron-rich DHIQ reduction obtained. Reproduced from ref. 105 with permission from the American Chemical Society. Copyright 2020.

Martin Wills and co-workers^105^ (2020) reported an efficient ATH of dihydroisoquinolines (DHIQs) using a chiral arene–Ru–TsDPEN catalyst system bearing heterocyclic modifications. A series of seven catalysts (360, 432–437) was screened using the reduction of 1-phenyl-DHIQ as a model substrate in the presence of a formic acid/triethylamine (FA/TEA) azeotrope in dichloromethane (DCM) (Scheme 24a). Among the tested catalysts, significant variations in activity and enantioselectivity were observed. Catalyst (*R*,*R*)-432 showed modest performance with 45% conversion and only 24% ee. The cymene-based catalyst (*R*,*R*)-433 was less effective, giving 11% conversion and 42% ee. Although the tethered catalyst (*R*,*R*)-360 achieved a high conversion of 97%, it delivered very poor ee (10%), indicating that high reactivity did not correlate with stereocontrol in this case. Catalyst (*R*,*R*)-434 was largely inactive, giving only 12% conversion with no enantioselectivity. In contrast, catalyst (*R*,*R*)-435 exhibited excellent performance, affording 93% conversion with 90% ee after 24 hours. The thiophene-containing catalyst (*R*,*R*)-436 also showed strong catalytic efficiency, delivering 86% conversion and 91% ee after 48 hours. Catalyst (*R*,*R*)-437 provided moderate conversion (77%) after 96 hours, but with reduced ee (49%). Based on these results, catalyst (*R*,*R*)-435 was identified as the optimal system for further studies. The substrate scope was then explored using a variety of non-electron-rich DHIQ derivatives. *Meta*- and *para*-substituted aromatic rings bearing chloro, bromo, iodo, methyl, methoxy, and trifluoromethyl groups were successfully reduced to the corresponding amines in moderate to good yields (50–90%) with enantioselectivities ranging from 76% to 93% (Scheme 24b). However, *ortho*-chloro-substituted substrates did not undergo reaction (441), while *ortho*-methyl and *ortho*-methoxy derivatives afforded only low yields (25% and 28%, 446 and 448), albeit with high enantioselectivity. This trend was attributed to steric hindrance arising from *ortho* substitution, which likely disrupts effective catalyst–substrate interaction. Electron-rich dimethoxy-substituted 1-aryl imine substrates were also compatible, affording amine products (453, 454, 456) in 72–82% yield with excellent ee (92–97%). In one case (455), a significantly lower yield (26%) was obtained, although ee remained high (95%). Additionally, 1-methyl imine substrates gave moderate yields (71–87%) with slightly lower enantioselectivities (80–81%) (457, 458). Mechanistically, stereocontrol is rationalized by a well-defined transition state involving hydrogen bonding between the substrate N–H and the sulfonyl oxygen of the TsDPEN ligand, along with stabilizing η^6^-arene/CH–π interactions. In the case of catalyst (*R*,*R*)-435, an additional favorable interaction between the oxygen atom of the heterocyclic (furan-containing) ligand and the π-system of the substrate further enhances enantioselectivity, accounting for its superior performance.

**Scheme 25:**
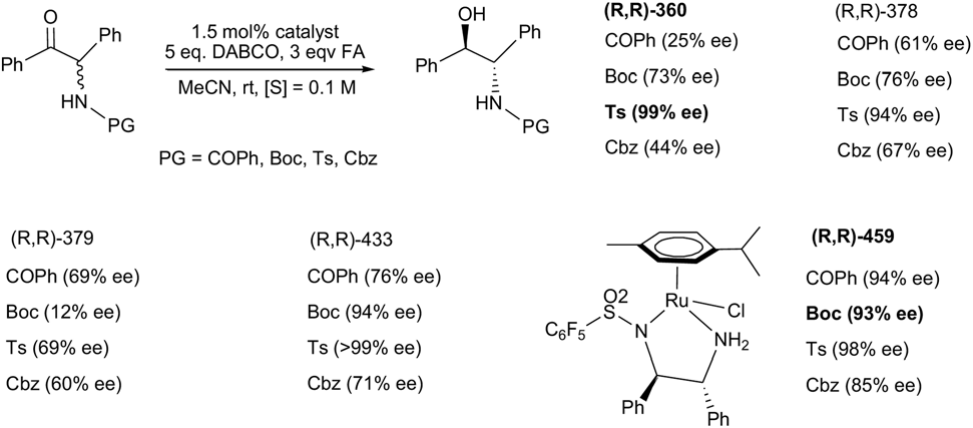
Catalyst screening of ATH reduction. Reproduced from ref. 106 with permission from the American Chemical Society. Copyright 2020.

**Scheme 26:**
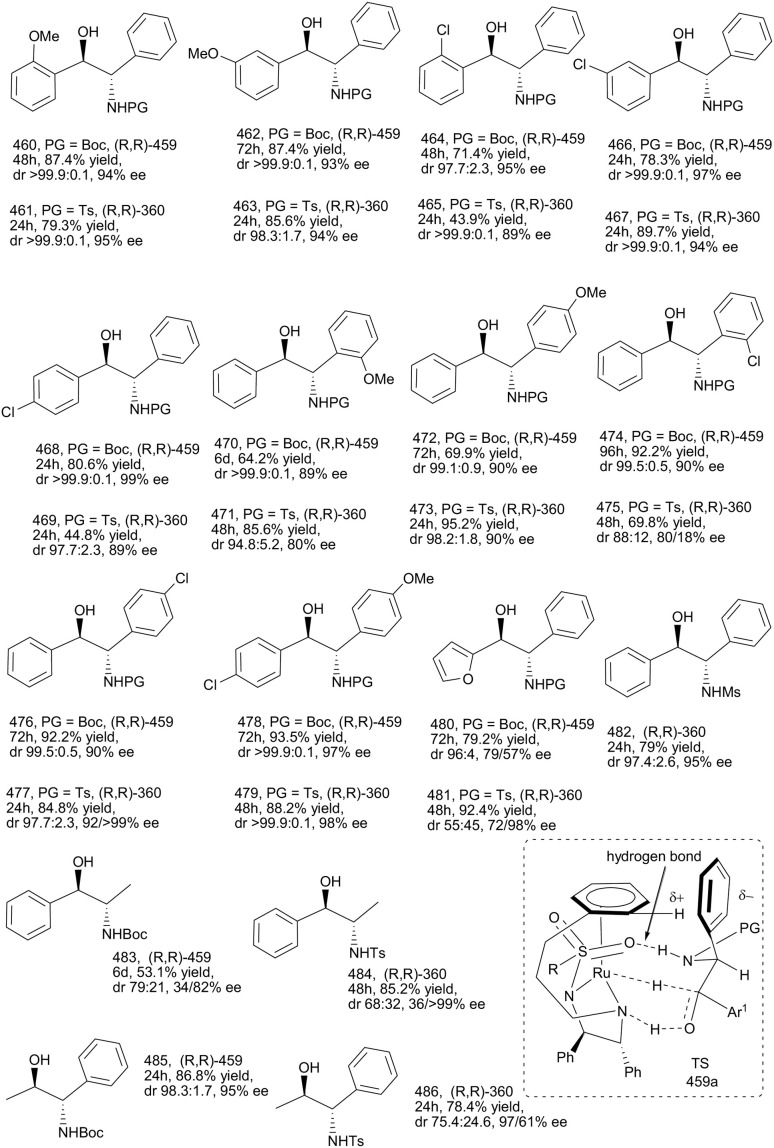
Reduction products of ketones and proposed mode of reduction. Reproduced from ref. 106 with permission from the American Chemical Society. Copyright 2020.

Martin Wills and co-workers^106^ (2020) demonstrated that α-amino ketones bearing a range of N-protecting groups can be efficiently reduced *via* a dynamic kinetic resolution–asymmetric transfer hydrogenation (DKR–ATH) process. The reaction delivered the corresponding amino alcohols with excellent stereocontrol, achieving enantioselectivities greater than 99% and diastereoselectivities >99.9 : 0.1. A series of Ru(ii)–TsDPEN catalysts of the type [(arene)Ru ((*R*,*R*)-TsDPEN) Cl] were evaluated, including catalysts (*R*,*R*)-360 and (*R*,*R*)-433, a pentafluorinated arene complex (*R*,*R*)-459, a CH_2_-linked arene catalyst (*R*,*R*)-360, and catalyst (*R*,*R*)-379 (Scheme 25). The catalytic activity is highly dependent upon the structure of catalyst and the protecting group. –COPh containing an N-protecting group produced the best ee (94%) with catalyst (*R*,*R*)-459, whereas catalyst (*R*,*R*)-360 yielded only 25% ee. The remaining catalysts provided moderate enantioselectivities in the range of 61–76% ee. With Boc-protected substrates, catalysts (*R*,*R*)-433 and (*R*,*R*)-459 showed similarly high ee (93–94%), while (*R*,*R*)-360 and (*R*,*R*)-378 gave moderate results (73–76% ee), and catalyst (*R*,*R*)-379 was significantly less effective (12% ee). For Ts-protected ketones, most catalysts exhibited excellent stereocontrol, delivering products with 94% to >99% ee. Only catalyst (*R*,*R*)-379 showed diminished performance, giving 69% ee. In the case of Cbz-protected substrates, catalyst (*R*,*R*)-459 again proved superior (85% ee), whereas the other catalysts afforded only moderate ee (60–71%), with catalyst (*R*,*R*)-360 giving 44% ee. The scope of the reaction was further explored using N-Boc and N-Ts protected α-amino ketones bearing various aryl substituents. Both electron-donating (OMe) and electron-withdrawing (Cl) groups at *ortho*-, *meta*-, and *para*-positions were well tolerated, affording the corresponding amino alcohols (460–469) with excellent enantioselectivity and dr (Scheme 26). Similarly, a wide range of aryl-substituted substrates (470–479) also underwent smooth reduction with high stereocontrol, although slightly diminished selectivity was observed for certain substrates such as 471 and 475. Heteroaryl substrates showed more variation in outcome. Furyl-containing ketones (480, 481) gave comparatively lower stereoselectivity. In contrast, mesyl (Ms)-protected substrates delivered excellent ee and dr (482). Aliphatic substituents adjacent to the amine generally led to reduced selectivity (483, 484), whereas alkyl groups adjacent to the ketone under Boc protection afforded excellent results (485). However, the corresponding Ts-protected analogue (486) showed poor enantioselectivity, highlighting the strong influence of protecting group effects. Mechanistically, the stereochemical outcome can be rationalized through a well-defined hydride-transfer transition state (TS-459a). Key stabilizing interactions include hydrogen bonding between the substrate N–H and the sulfonamide oxygen of the catalyst, along with favorable CH/π edge-to-face interactions between the arene ligand and the substrate framework. These interactions collectively govern the observed high levels of stereocontrol in the DKR–ATH process.

**Scheme 27:**
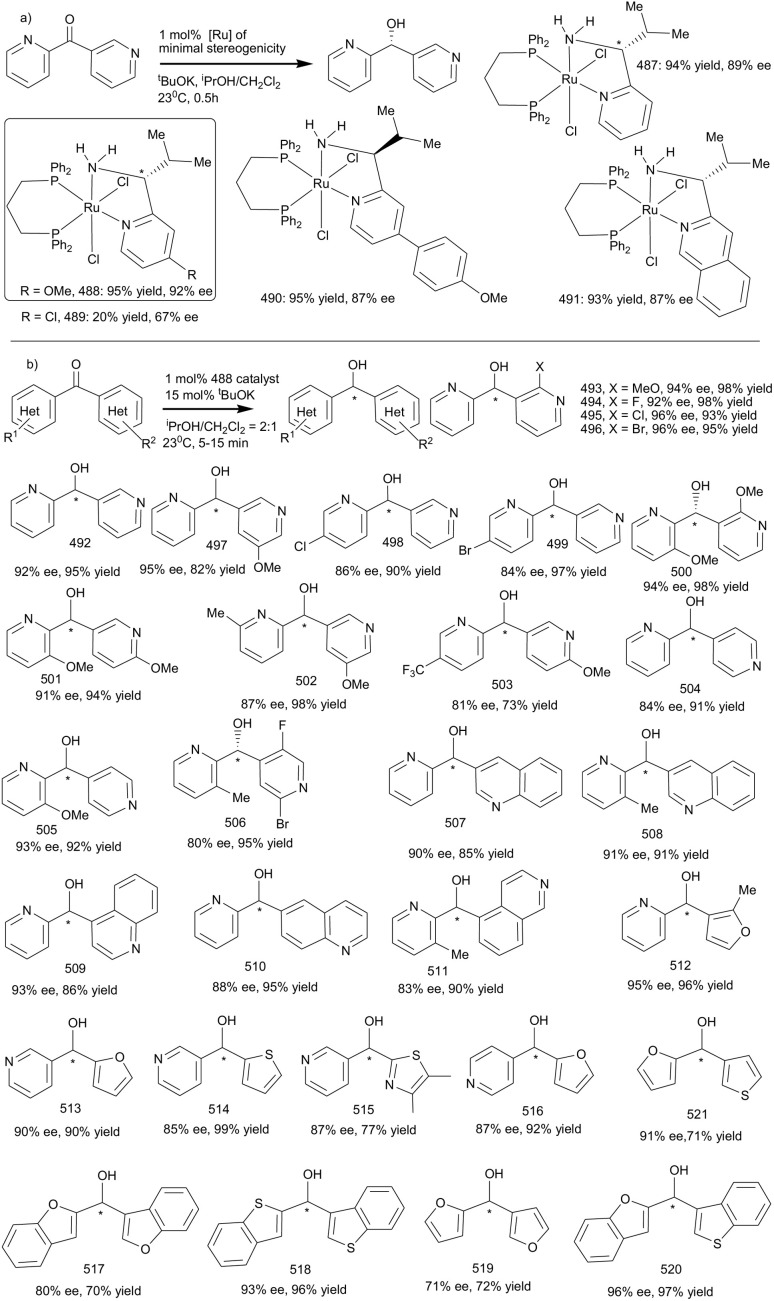
(a) Catalyst screening. (b) Substrate scope for diheteroaryl ketones. Reproduced from ref. 107 with permission from the American Chemical Society. Copyright 2020.

Min-Jie Zhou and Xiangyou Xing's group^107^ (2020) reported an efficient ATH of diheteroaryl ketones and diaryl ketones using a ruthenium catalyst system. This methodology provided access to chiral diheteroarylmethanols and benzhydrol derivatives in generally good to excellent yields and enantioselectivities. In the model reaction, pyridin-2-yl(pyridin-3-yl) methanone was reduced using the Ru–(*S*)-^*i*^Pr-Pyme catalyst (487), affording the corresponding alcohol in 94% yield with 89% ee (Scheme 27). Catalyst modification significantly influenced stereoselectivity. In particular, catalyst 488, bearing an electron-donating methoxy group at the *para* position of the pyridyl ligand, improved enantioselectivity to 92% ee. In contrast, catalyst 489, which contains an electron-withdrawing chloro substituent, led to a reduced enantioselectivity of 67% ee. Catalysts 490 and 491, featuring more electron-rich and extended conjugated diamine ligands, provided results comparable to catalyst 487, with around 87% ee. Among the tested systems, catalyst 488 was identified as the most effective, and its structure was confirmed by single-crystal X-ray analysis. The substrate scope was broad and well tolerated. Substituted diheteroaryl ketones bearing electron-donating and electron-withdrawing groups at the 2- and 5-positions of the pyridyl ring were smoothly reduced, affording the corresponding alcohols in high yields (82–98%) and excellent enantioselectivities (92–96% ee) (493–499). Halogen-substituted derivatives at the 4-position of the 2-pyridyl ring also performed well, giving good ee (84–86%) with excellent yields (90–97%). Disubstituted substrates further demonstrated the robustness of the catalytic system, providing products 500–503 with good to excellent enantioselectivities (81–94% ee). Ketones containing pyridin-2-yl(pyridin-3-yl) and pyridin-2-yl(pyridin-4-yl) frameworks were also efficiently reduced, affording alcohols 504–507 in 91–95% yield and 80–93% ee. The absolute configuration of selected products (500 and 506) was confirmed by single-crystal X-ray diffraction. The methodology was further extended to a wide range of heteroaryl–heteroaryl ketones. Excellent catalytic performance was observed for substrates containing pyridyl–quinolinyl and pyridyl–isoquinolinyl motifs, delivering products 507–511 in 85–95% yield and 83–93% ee. Ketones incorporating furan, thiophene, and thiazole units were also efficiently reduced, giving products 512–516 with 77–99% yield and up to 95% ee. Interestingly, diheteroaryl ketones without 2-pyridyl groups were reduced to produce alcohol (517–521), which had never been reduced using a catalyst before, yielding an intriguing result. The ketone substrate containing bis-benzofuran, bis-benzo[*b*]thiophene, and bis-furan rings smoothly reduced to alcohol products 517 (80% ee, 70% yield), 518 (93% ee, 96% yield), and 519 (71% ee, 72% yield). Benzo[*b*]thiophen-3-yl(benzofuran-2-yl) methanone and furan-2-yl(thiophen-3-yl) methanone were furnished in this procedure, yielding an alcohol product (520: 96% ee, 521: 91% ee). As a result, this study demonstrates the robustness of Ru-catalyzed ATH in the enantioselective reduction of broad ranging diheteroaryl ketones, with catalyst structure playing an important role in controlling stereochemical outcomes.

**Scheme 28:**
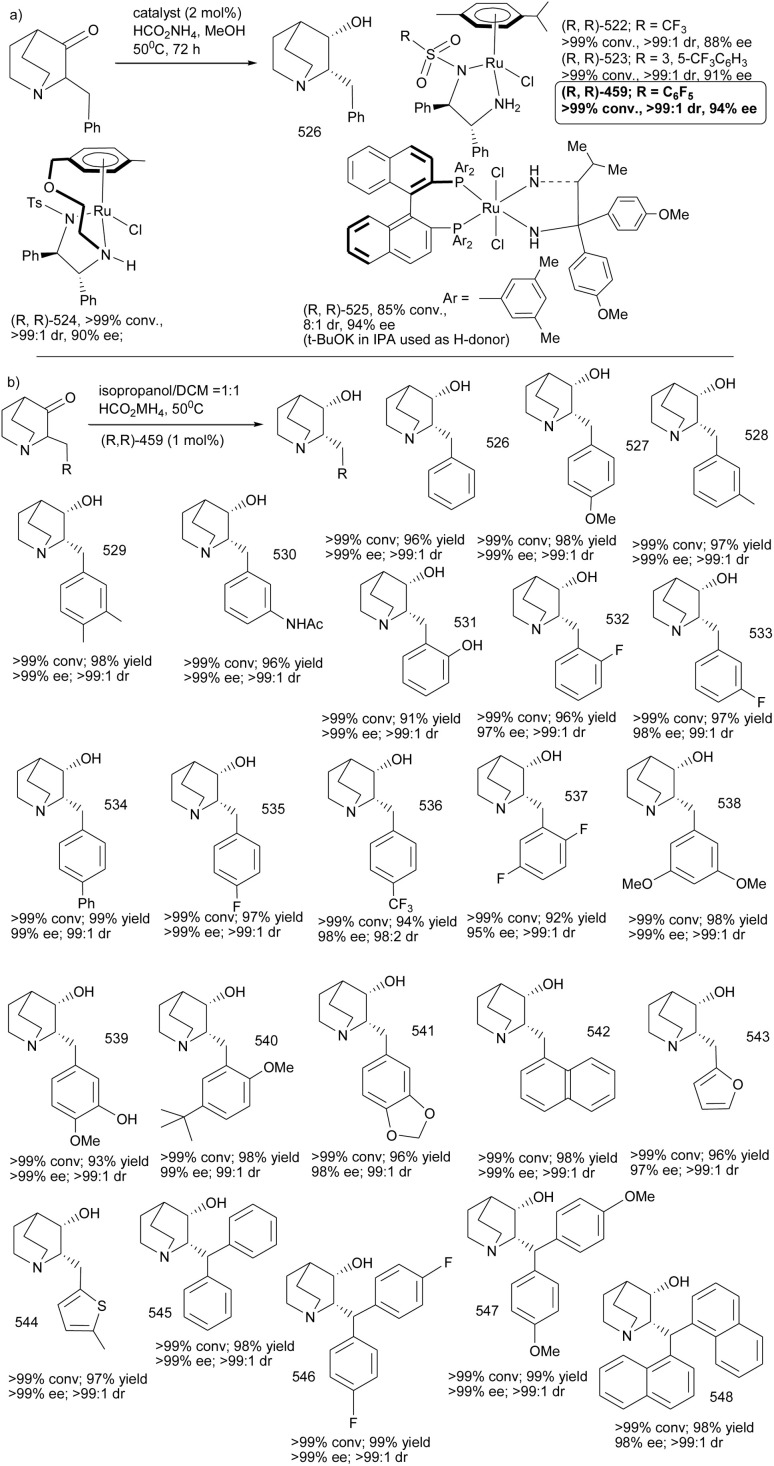
(a) Screening of catalysts for ATH of 3-quinuclidinone. (b) DKR-ATH of 3-quinuclidinone substrates catalyzed by (*R*,*R*)-459. Reproduced from ref. 108 with permission from the American Chemical Society. Copyright 2020.

The Lei Zhang group^108^ (year 2020) reported a successful and effective synthesis of *cis*-3-quinuclidinols *via* an ATH process with a variety of functionalised substituents at the 2-position. Several widely utilised Ru-diamine complexes [(*R*,*R*)-522 -(*R*,*R*)-525 and (*R*,*R*)-459] were employed as a catalyst, and HCO_2_H/Et_3_N used as a hydrogen donor (Scheme 28). Nearly all of the examples resulted in quantitative conversions, but most of them produced moderate to low enantioselectivities (63–75% ee). Despite having a poor dr of 14 : 1, only catalyst (*R*,*R*)-522 demonstrated good catalytic activity with an ee of 91%. Then it was found that HCO_2_NH_4_ is more efficient hydrogen donor compared to HCO_2_H/Et_3_N, delivering enhanced enantioselectivities (88–94% ee). This result indicates that the selectivity of the ATH reaction is significantly influenced by the hydrogen donor. Further optimization identified catalyst (*R*,*R*)-459 in combination with HCO_2_NH_4_ as the most effective system. Solvent screening showed that polar aprotic solvents such as DCM or ethyl acetate alone were less efficient, whereas isopropanol (IPA), either alone or in combination with DCM, significantly improved enantioselectivity. The optimal conditions were achieved using IPA/DCM (1 : 1, v/v) at 50 °C for 72 hours with 1.0 mol% of (*R*,*R*)-459 and HCO_2_NH_4_ as hydrogen source. Under these optimized conditions, a broad range of 2-arylmethyl-3-quinuclidinone derivatives were reduced smoothly to the corresponding *cis*-3-quinuclidinols with excellent results, delivering up to 99% yield, >99 : 1 dr, and up to 99% ee (526–544) (Scheme 28b). Substituent effects on the aromatic ring were found to be minimal. Electron-donating groups such as methyl and methoxy generally afforded slightly higher enantioselectivities (>99% ee), whereas electron-withdrawing groups such as fluoro and trifluoromethyl gave slightly reduced but still high enantioselectivities (96–97% ee). Heteroaromatic substrates were also well tolerated, affording products 543 and 544 with 97% ee and >99% ee, respectively. Additionally, 2-diarylmethyl-3-quinuclidinone derivatives (545–548) were reduced with excellent stereocontrol, providing products with 98–99% ee and >99 : 1 dr. Importantly, the methodology was successfully extended to gram-scale synthesis using only 0.2 mol% catalyst loading, affording product 526 quantitatively with >99% ee and >99 : 1 dr. Overall, this study demonstrates that fine tuning of both the hydrogen source and reaction medium, in addition to catalyst selection, is essential for achieving high stereocontrol in the ATH of quinuclidinone derivatives.

**Scheme 29:**
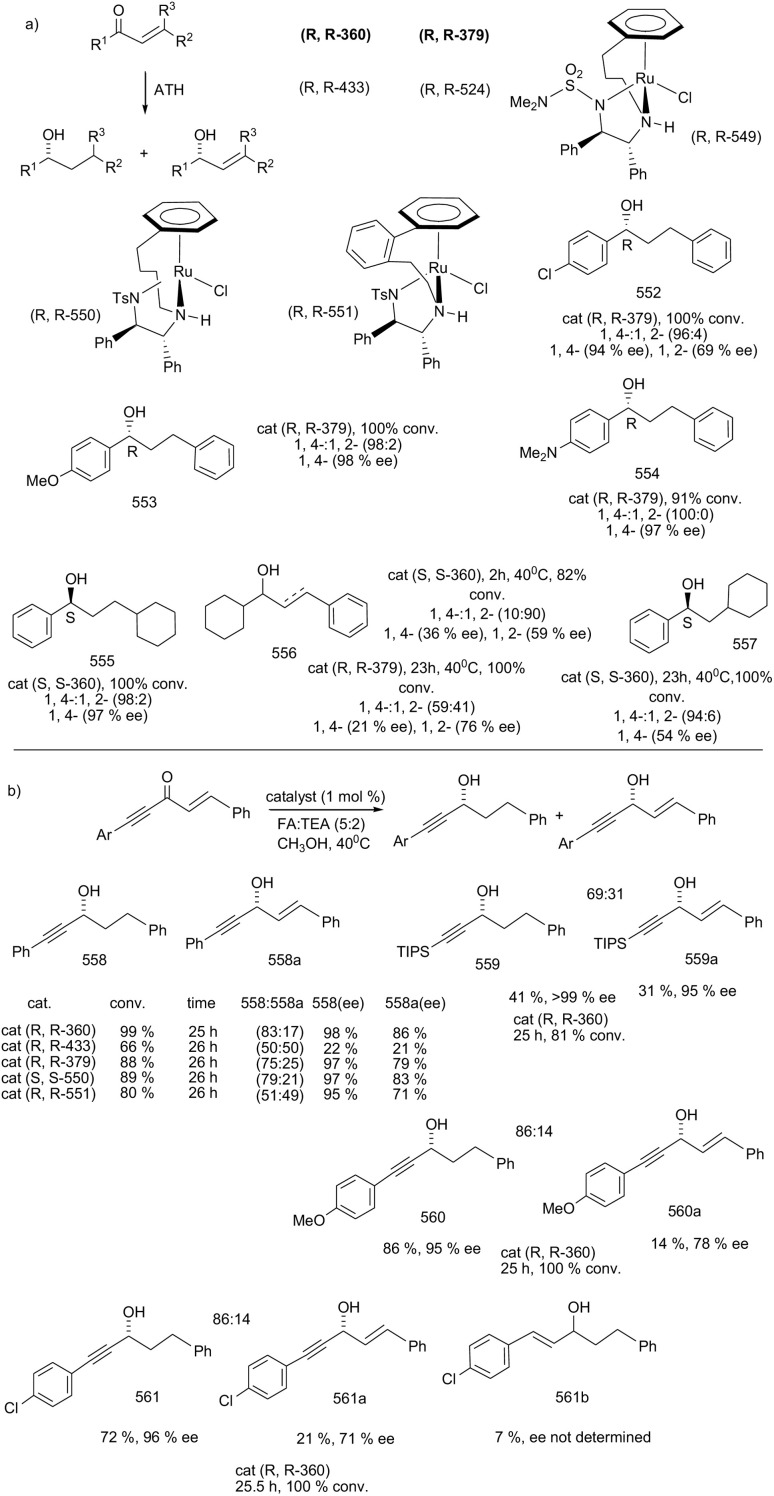
(a) Products of ATH of chalcone derivatives. (b) Products of ATH of alkene/alkyne substrates. Reproduced with permission from ref. 109. Copyright 2021 Elsevier.

Martin Wills *et al.*^109^ (2021) reported the ATH of enones using ruthenium-based catalysts, employing a range of substrates bearing both electron-withdrawing (Cl) and electron-donating (OMe, NMe_2_) substituents. The reactions were carried out using formic acid/triethylamine as the hydrogen source in the presence of catalyst 379 (Scheme 29). Under these conditions, *para*-substituted enones showed excellent chemoselectivity for 1,4-reduction over 1,2-reduction. In the case of the *para*-chloro derivative, a 96 : 4 ratio of 1,4- to 1,2-addition products was observed, with 94% ee for the major product. Improved selectivity was obtained with electron-donating substituents: the *para*-methoxy substrate gave a 98 : 2 ratio with 98% ee, while the *para*-dimethylamino analogue showed complete 1,4-selectivity (100 : 0) with 97% ee (554). Substrate structure also played a key role in controlling selectivity. A cyclohexyl-substituted enone adjacent to the alkene underwent efficient reduction with catalyst 360, affording a 98 : 2 ratio of 1,4- to 1,2-addition products and 97% ee (555). In contrast, when the cyclohexyl group was positioned adjacent to the carbonyl group, the selectivity was reversed (10 : 90), and reduced enantioselectivity was observed (36% ee for 1,4-product and 59% ee for 1,2-product) (556). With catalyst 379, this substrate still gave poor chemoselectivity (59 : 41), although modest improvements in enantioselectivity were observed (21% and 76% ee). Enones bearing both alkyne and alkene functionalities also participated effectively in the reaction. Using catalyst 360, an 83 : 17 ratio of 1,4- to 1,2-reduction products was obtained (558/558a), with high ee (98% and 86%, respectively). Similar chemoselectivity was observed with catalysts 433 and 551, although catalyst 433 gave low ee (21–22%), whereas catalyst 551 afforded significantly higher values (up to 95% ee and 71% ee). Catalysts 379 and 550 also showed comparable selectivity patterns with good enantioselectivity overall. The influence of steric and electronic effects was further demonstrated in substrates containing bulky TIPS-substituted alkynes, which gave 69 : 31 product ratios and excellent ee (>99% ee and 95%). Electron-rich aryl systems such as methoxy-substituted rings showed slightly reduced chemoselectivity (86 : 14), but still maintained good ee (up to 95% and 78%). In contrast, chloro-substituted aromatic systems maintained high selectivity for the 1,4-addition pathway, delivering products with 96% ee. Overall, this study highlights that both the electronic nature of substituents and steric environment strongly influence chemoselectivity (1,4 *vs.* 1,2 addition) as well as enantioselectivity in ruthenium-catalyzed ATH of enones.

**Scheme 30:**
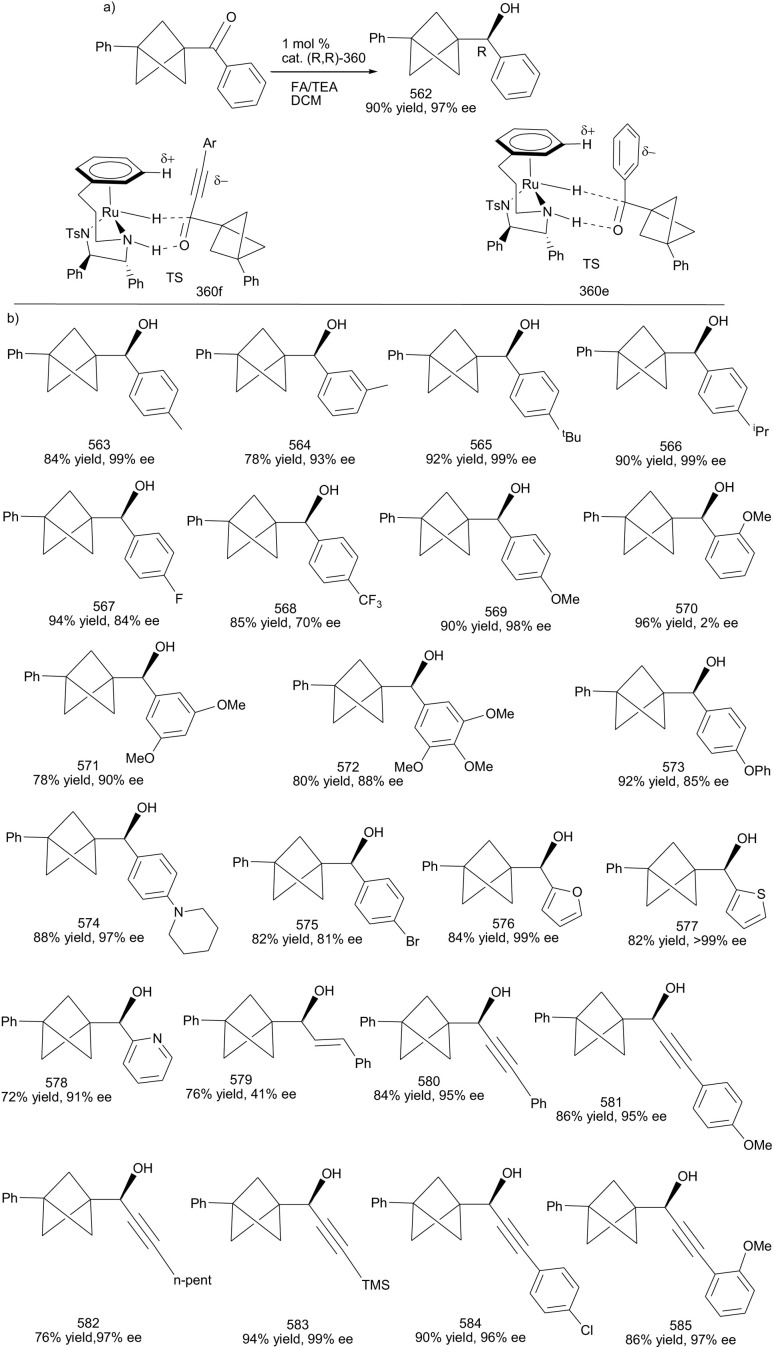
(a) Proposed binding mode of BCP ketones ATH. (b) Products of ATH of BCP derivatives catalysed by (*R*,*R*)-360. Reproduced from ref. 110 with permission from the American Chemical Society. Copyright 2021.

Martin Wills and co-workers^110^ (2021) reported the ATH of bicyclo [1.1.1] pentane (BCP)-containing ketones using ruthenium catalysts. In the presence of a tethered Ru(ii) catalyst (*R*,*R*)-360, formic acid/triethylamine (FA/TEA) served as the hydrogen source in dichloromethane (DCM), affording the corresponding alcohol in 90% yield with 97% enantiomeric excess (ee) (Scheme 30a). The absolute configuration of the product was established as *R* by single-crystal X-ray diffraction. A mechanistic model suggested that the bulky BCP framework is oriented away from the η^6^-arene surface in the key transition state (TS-360e), enabling efficient asymmetric induction. A range of substituted aromatic derivatives was then evaluated. *Para*-substituted aryl–alkyl ketones generally exhibited excellent ee (up to 99% for 563, 565, and 566), while a *meta*-methyl substituent led to a slight decrease (93% ee for 564) (Scheme 30b). Electron-withdrawing groups such as fluoro and trifluoromethyl resulted in reduced ee (84% and 70% for 567 and 568, respectively), attributed to weaker π–arene interactions with the catalyst's η^6^-arene unit. In contrast, electron-donating methoxy substituents delivered consistently high ee (88–98% for 569, 571, and 572). Notably, the *ortho*-methoxy derivative showed a dramatic drop in selectivity (570, 2% ee), likely due to steric repulsion that disrupts the optimal transition-state geometry. Other *para*-substituted groups, including phenoxy, amino, and bromo substituents, were also well tolerated, giving products with 85% ee, 97% ee, and 81% ee, respectively (573–575). Heteroaromatic substrates such as furan, thiophene, and pyridine performed particularly well, affording products with excellent enantioselectivities (up to >99% ee for 576 and 577, and 91% ee for 578). However, enone-containing substrates showed significantly reduced ee (579, 41%), indicating limited compatibility of highly conjugated systems. A broad range of alkynyl substrates, including phenyl-, methoxyphenyl-, chlorophenyl-, alkyl-, and trimethylsilyl-substituted derivatives, were successfully reduced, delivering the corresponding alcohols in excellent yields and high enantioselectivities (95–99% ee for 580–585). The absolute configuration of compound 584 was confirmed as *R* by single-crystal X-ray analysis. A proposed transition-state model (TS-360f) accounts for the observed stereochemical outcome in alkynyl-BCP ketone reductions.

**Scheme 31:**
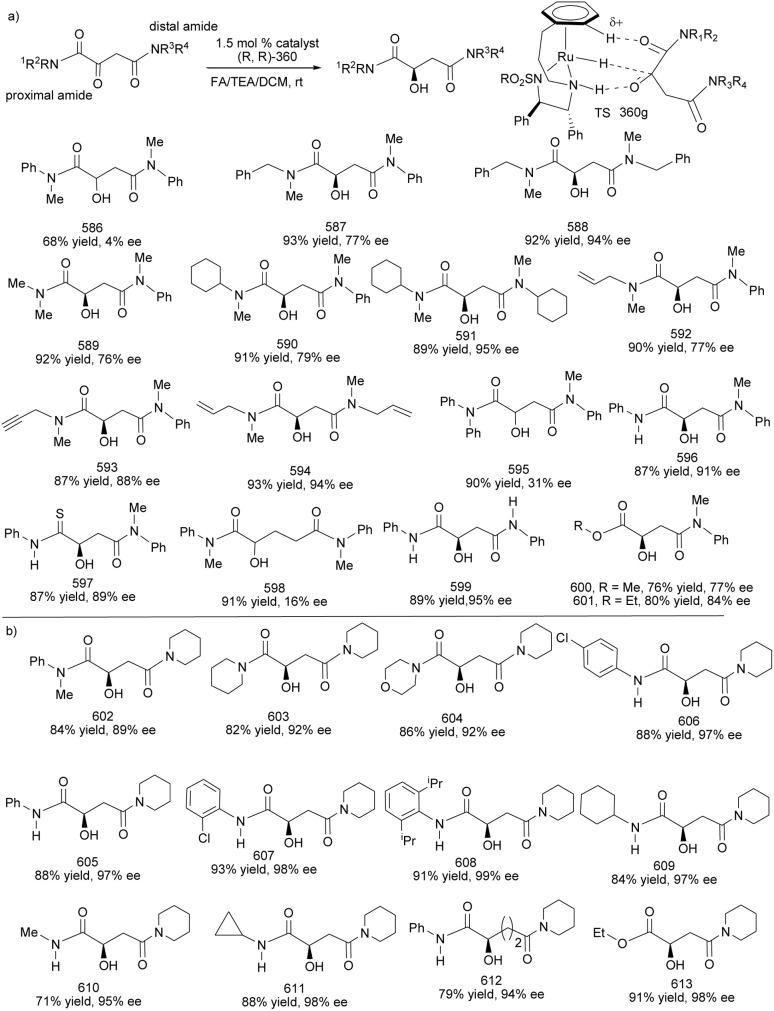
Products of ATH of (a) distal amide NMePh in most cases. (b) distal amide piperidine-derived. Reproduced from ref. 111 with permission from the American Chemical Society. Copyright 2021.

**Scheme 32:**
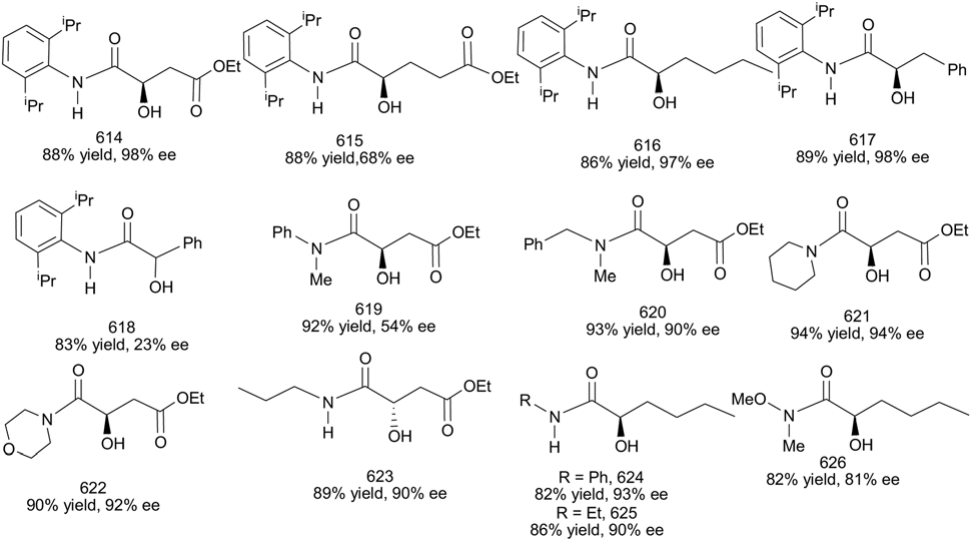
Products of ATH of extended range of amide.

Martin Wills *et al.*^111^ (2021) reported the ATH of α-keto-1,4- diamides using a Ru(ii) Noyori–Ikariya type catalyst, achieving alcohol products with a broad range of enantioselectivities depending on substrate substitution. The reaction employed a 3C-tethered ruthenium catalyst (*R*,*R*)-360, with formic acid (FA) and triethylamine (TEA) as the hydrogen source in dichloromethane under mild conditions. A key finding of this study was the strong influence of amide substituents on enantioselectivity. When both amide positions contained *N*-methylphenyl (NMePh) groups, the reaction gave very low ee (Scheme 31a, 4%, 586). However, replacing one amide with *N*-methylbenzyl (NMeBn) significantly improved enantioselectivity to 77% (587), while substitution on both amide sites with NMeBn further enhanced selectivity to 94% ee (588). Similarly, substrates bearing *N*, *N*-dimethyl (NMe_2_) and *N*-methylcyclohexyl (NMeCy) substituents afforded moderate to good enantioselectivities (76–79% ee, 589–590), whereas dual NMeCy substitution led to excellent ee (95%, 591). Other structural variations also showed notable trends. Allyl and alkyne substituents produced alcohols with 77% and 88% ee, respectively (592–593), and dual allyl substitution improved enantioselectivity to 94% ee (594). In contrast, diphenyl amide (NPh_2_) substitution resulted in poor ee (31%, 595), while secondary aromatic amides (PhNH) and thioamide analogues gave significantly improved outcomes (91% and 89% ee, 596–597). Extension of the carbon backbone in related systems led to reduced ee (16%, 598), whereas NHPh-containing systems delivered high ee (95% ee, 599). Ester-containing substrates also participated effectively, giving products with 77–84% ee (600–601). Incorporation of piperidine in the distal amide further improved stereocontrol (Scheme 31b). A series of more complex substrates (605–611), containing both aromatic and alkyl substituents in the proximal amide, showed consistently high enantioselectivity. In particular, sterically bulky substituents enhanced enantioinduction, with compound 608 giving the best result, highlighting the importance of steric and electronic effects in catalyst–substrate interactions. The absolute configuration of 606 was R, determined from a single X-ray crystallography structure. Additionally, the ester derivative produced an alcohol product with 98% ee (613); the proposed mode for the formation of alcohol was illustrated in TS-360g. Substrates with hindered amides adjacent to ketones were subjected to ATH (compounds 614–618) (Scheme 32). Ester groups in the distal amide were reduced to give an alcohol product (614) with an ee (98%), but increasing the carbon length in the alkane chain decreased the ee (68%, 615). The absolute configuration 614 was confirmed by X-ray crystallography structure; substrates containing butyl and benzyl groups yielded excellent ee (97%, 98%, 616, 617), respectively, while β-phenyl-containing amides showed lower ee (618, 23%). These studies revealed how the steric factor influences the stereoselectivity. Additionally, ester-containing substrates with NMePh produced alcohol products with lower ee (619, 54%); whereas, substrates with NMeBn, piperidine, morpholine, and *N*-alkyl groups produced alcohol products with very good ee (90% to 94%, 620–623). A similar trend was observed for butyl group-containing substrates with NHPh, NHEt, NMeOMe, and OEt, which yielded corresponding alcohol products with 93% ee, 90% ee, 81% ee, and 80% ee, respectively (624–626). Notably, when a substrate contained both ester and amide functional groups, the resulting alcohol product had an enantioselectivity of only 4% ee.

**Scheme 33:**
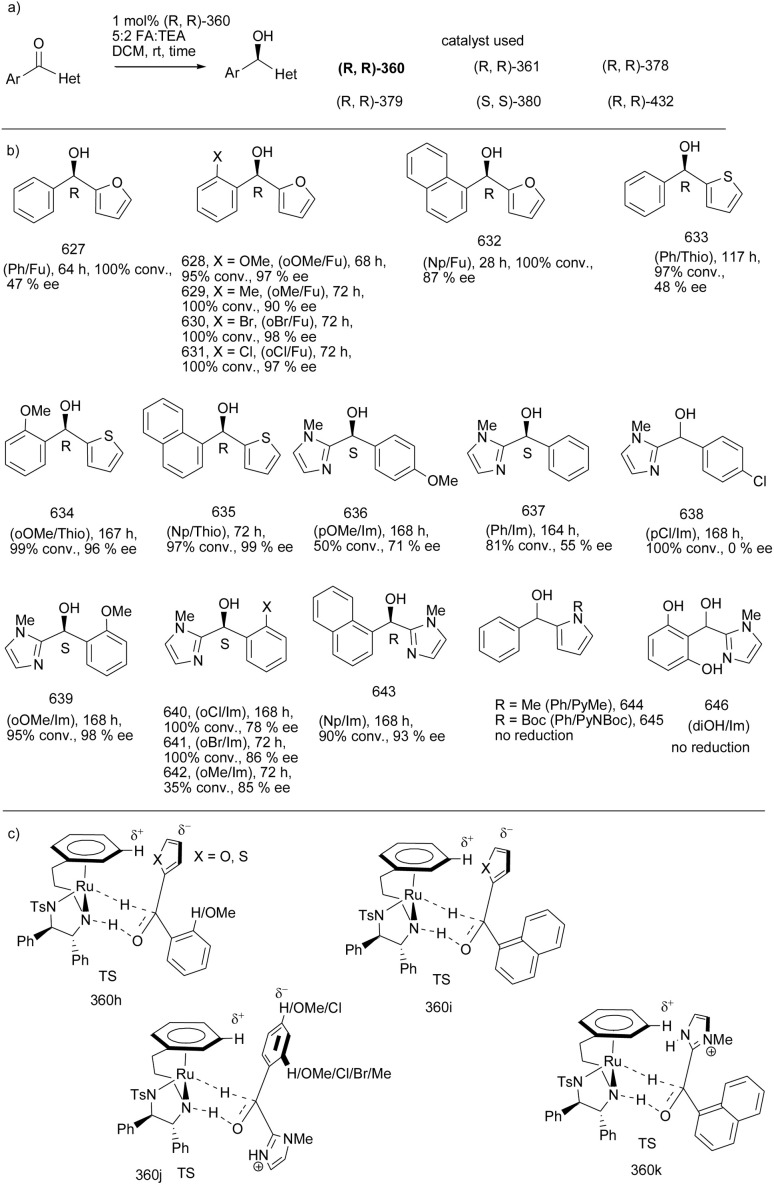
(a) Catalyst used in this reaction. (b) Products of ATH formed in this study. (c) Proposed favoured mode of hydrogen transfer. Reproduced from ref. 112 with permission from Wiley-VCH. Copyright 2021.

Martin Wills^112^ (year 2021) reported that heterocyclic rings containing ketones can be reduced by ruthenium catalysts to yield products with up to 99% ee. Several catalysts employed in these studies include (*R*,*R*)-432, tethered systems (*R*,*R*)-360 and (*R*,*R*)-361, benzyl-tethered (*R*,*R*)-378, OMe-tethered (*R*,*R*)-379, and cymene-based (*S*,*S*)-380. The reactions were performed using a formic acid/triethylamine (FA/TEA) mixture in dichloromethane under mild conditions (Scheme 33a). Among the tested catalysts, (*R*,*R*)-360 exhibited the highest catalytic activity, achieving complete conversion in a shorter reaction time compared to others. In the reduction of a furan–phenyl ketone substrate, catalyst (*R*,*R*)-378 provided the highest ee (61%) within this specific set. Substrates bearing *ortho*-methoxy, methyl, bromo, and chloro substituents on the phenyl ring underwent efficient reduction with excellent ee (90–98%, 628–631), particularly with catalyst (*R*,*R*)-360. Other catalysts, such as (*R*,*R*)-378 and (*S*,*S*)-380, delivered comparable enantioselectivities (88–89% ee), whereas catalyst (*R*,*R*)-379 showed significantly lower selectivity (56% ee). Naphthyl- and furan-containing ketones were also well tolerated, giving consistently high ee (87–94%) across different catalysts, with (*R*,*R*)-360 again showing superior activity. The absolute configuration of product 632 was determined to be *R* by single-crystal X-ray diffraction. Thiophene-containing ketones were also examined. In the case of phenyl-substituted substrates, catalyst (*R*,*R*)-378 gave the highest ee (69%), while (*R*,*R*)-360 afforded 48% ee (633). Interestingly, substrates bearing *ortho*-methoxy substitution showed significantly improved ee (up to 96%, 634). Naphthyl–thiophene ketones were reduced with excellent outcomes, giving up to 99% ee (635) with catalysts (*R*,*R*)-360 and (*R*,*R*)-378, while (*R*,*R*)-379 gave 80% ee and (*S*,*S*)-380 provided 97% ee. The enantioselectivity trends were rationalized through transition-state models (TS-360h and TS-360i), which suggest that electron-rich substrates preferentially interact with the η^6^-arene region of the catalyst, influencing hydride transfer and stereochemical outcome (Scheme 33c). Further studies on *N*-methyl imidazole-containing ketones revealed a broad range of enantioselectivity (0–98% ee) depending on substituents. Electron-donating groups such as methoxy afforded the highest enantioselectivity (636), whereas electron-withdrawing groups such as chloro led to modarate selectivity (638). For *ortho*-substituted derivatives, significantly improved enantioselectivity was observed (78–98% ee), with the methoxy-substituted substrate giving the best result (639, 98% ee), while chloro, bromo, and methyl substituents afforded 78%, 86%, and 85% ee, respectively (640–642). A naphthyl-containing substrate also gave high ee (643, 93%). The absolute configurations of key products (639 and 643) were determined as *S* and *R*, respectively, supporting the proposed binding models (TS-360j and TS-360k), which highlight distinct orientations of the substrate relative to the η^6^-arene surface of the catalyst. Finally, substrates bearing two hydroxyl groups were found to be incompatible with this ATH system, as no reduction occurred (646), likely due to strong coordination or deactivation of the catalyst.

**Scheme 34:**
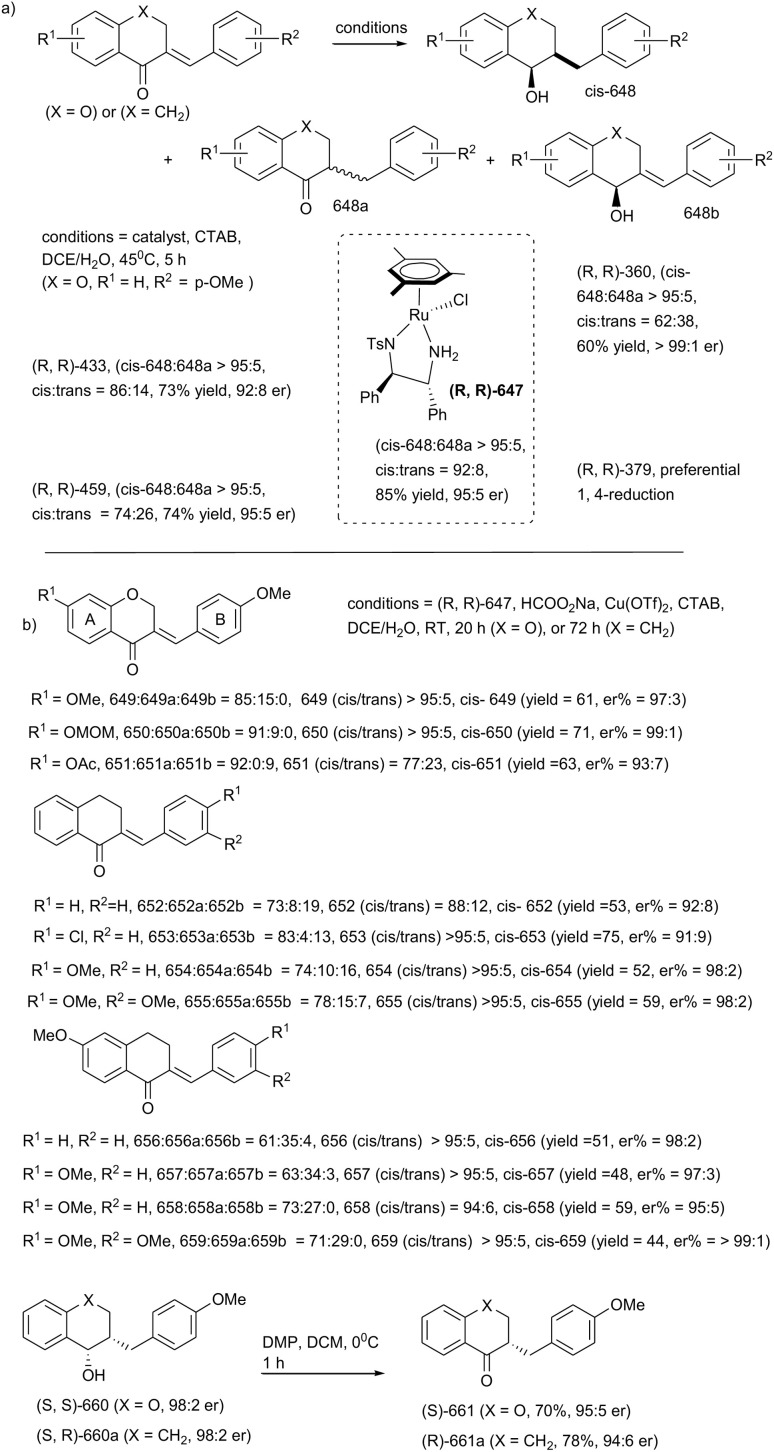
(a) Screening the optimized reaction conditions. (b) Scope of the ATH–DKR of enone catalyzed by (*R*,*R*)-647. Reproduced from ref. 113 with permission from the American Chemical Society. Copyright 2021.

Paulo R. R. Costa *et al.*^113^ (2021) reported an ATH process that efficiently affords chiral alcohol 648 and intermediate 648a using a Ru(ii) Noyori–Ikariya catalyst. The reaction was performed with 5 mol% of catalyst (*R*,*R*)-433, an enone substrate (X = O, R^1^ = H, R^2^ = OMe), and 20 mol% cetyltrimethylammonium bromide (CTAB) as a phase-transfer catalyst in a DCE/H_2_O solvent system at room temperature for 20 hours. The transformation proceeds *via* a DKR pathway, delivering predominantly the *cis*-configured (*R*,*R*)-alcohol 648 (Scheme 34a). To improve stereoselectivity, various reaction parameters were systematically optimized. In methanol, although the *cis*/*trans* ratio remained unchanged (72 : 28), the product ratio (648/648a >95 : 5) and er improved significantly to 97 : 3. In contrast, using MeOH/H_2_O led to reduced dr (62 : 38) and lower er (88 : 12), whereas DCE/H_2_O improved the *cis*/*trans* ratio (82 : 18) but decreased enantioselectivity. The use of neutral or anionic phase-transfer catalysts, as well as additives such as Na_2_CO_3_ or HCO_2_H, did not provide any further enhancement in stereocontrol. Interestingly, shortening the reaction time from 20 hours to 5 hours improved stereoselectivity, suggesting that prolonged reaction times may promote secondary processes that affect selectivity. A series of related catalysts (360, 379, 459, 647) was also evaluated. All of them improved stereoselectivity compared to catalyst 433, with catalyst (*R*,*R*)-647 providing the best overall balance between dr and er (92 : 8 *cis*/*trans*, 95 : 5 er). Although catalyst (*R*,*R*)-360 delivered the highest er (>99 : 1), it did so at the expense of reduced dr. The superior performance of catalyst 647 was attributed to favorable electrostatic and CH⋯π interactions between the substrate and the η^6^-mesitylene ligand. The methodology was further applied to the synthesis of substituted homoisoflavones. Substrates bearing electron-donating protecting groups such as OMe and OMOM afforded products 649 and 650 in good yields (85–91%) with excellent dr (>95 : 5) and very high er (up to 99 : 1). However, substrates containing an acetate (OAc) group showed slightly reduced stereocontrol (651, 93 : 7 er) along with the formation of a minor allylic alcohol byproduct (651b, 9%). The reactivity of chromanone-based enones was also investigated.

**Scheme 35:**
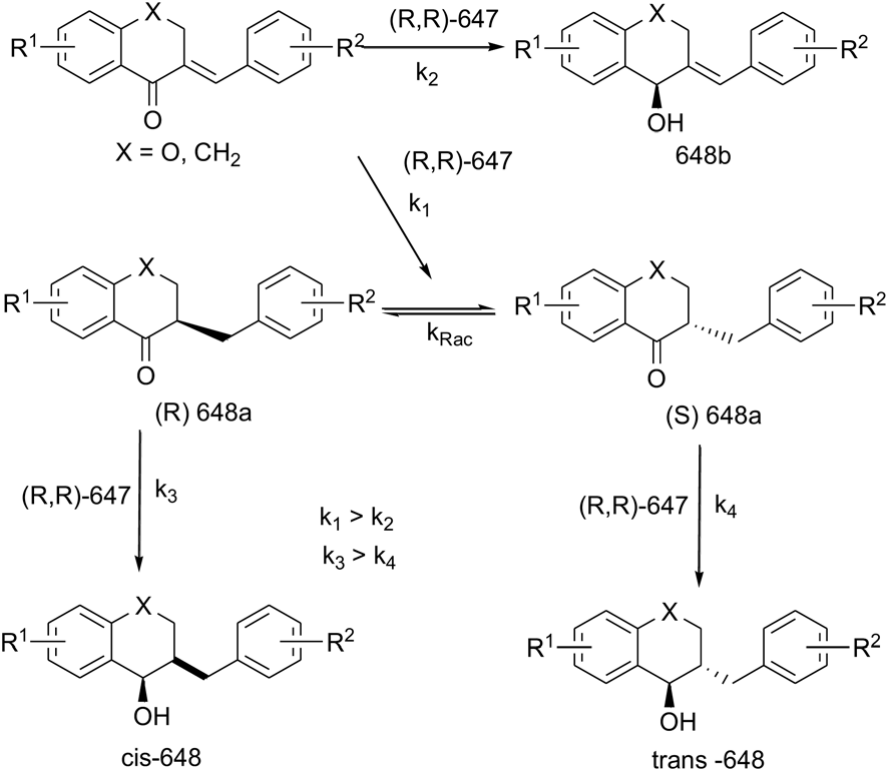
Mechanistic proposal for the reductions of enones.

Substituents on the A-ring significantly influenced the reaction outcome: unsubstituted substrates were more reactive, giving *cis*-products (652–655) with high dr and er, along with small amounts of allylic alcohols (652b–655b). In contrast, methoxy-substituted A-ring substrates exhibited reduced reactivity and led to higher proportions of unreacted ketone (656a–659a, 29–35%), although the major *cis*-products (656–659) were still obtained with good stereocontrol. Extension of reaction time to 72 hours at room temperature improved conversion in these cases. Further synthetic utility was demonstrated using catalyst (*S*,*S*)-647, which enabled access to alcohols *cis*-660 and *cis*-660a. Subsequent oxidation with Dess–Martin periodinane (DMP) afforded enantiomerically enriched ketones (*S*)-661 and (*R*)-661a with excellent optical purity. Mechanistically, the reaction is proposed to proceed through initial 1,4-reduction of the enone to form intermediate 648a, which exists in equilibrium with its enol form. Although 1,2-reduction to 648b is also possible, it occurs more slowly than 1,4-reduction. The key step involves DKR of intermediate 648a under Ru–H catalysis, leading selectively to the *cis*-alcohol 648 with high stereocontrol (Scheme 35).

Virginie Ratovelomanana-Vidal *et al.*^114^ (2021) reported the ATH of 3-fluorochromanone derivatives to access *cis*-3-fluorochroman-4-ols using Ru(ii)-based Noyori–Ikariya type catalysts. The transformation proceeds through a DKR pathway, enabling the formation of two adjacent stereogenic centers with high stereocontrol. Initially, 3-fluorochroman-4-one was reduced using catalyst (*R*,*R*)-433 with a 5 : 2 mixture of formic acid and triethylamine in dichloromethane, affording *cis*-662 in 95% yield with 95% ee; however, the dr was moderate (58 : 42) under these conditions. In contrast, when the reaction was performed in an acetonitrile solution with a 1 : 1 ratio of HCO_2_H and Et_3_N, very good yields were achieved (95%) with excellent ee (99%) with high dr (97 : 3). The catalyst (*R*,*R*)-524 also showed impressive results with a yield of 87% and outstanding ee (>99%) with very good dr (96 : 4).

**Scheme 36:**
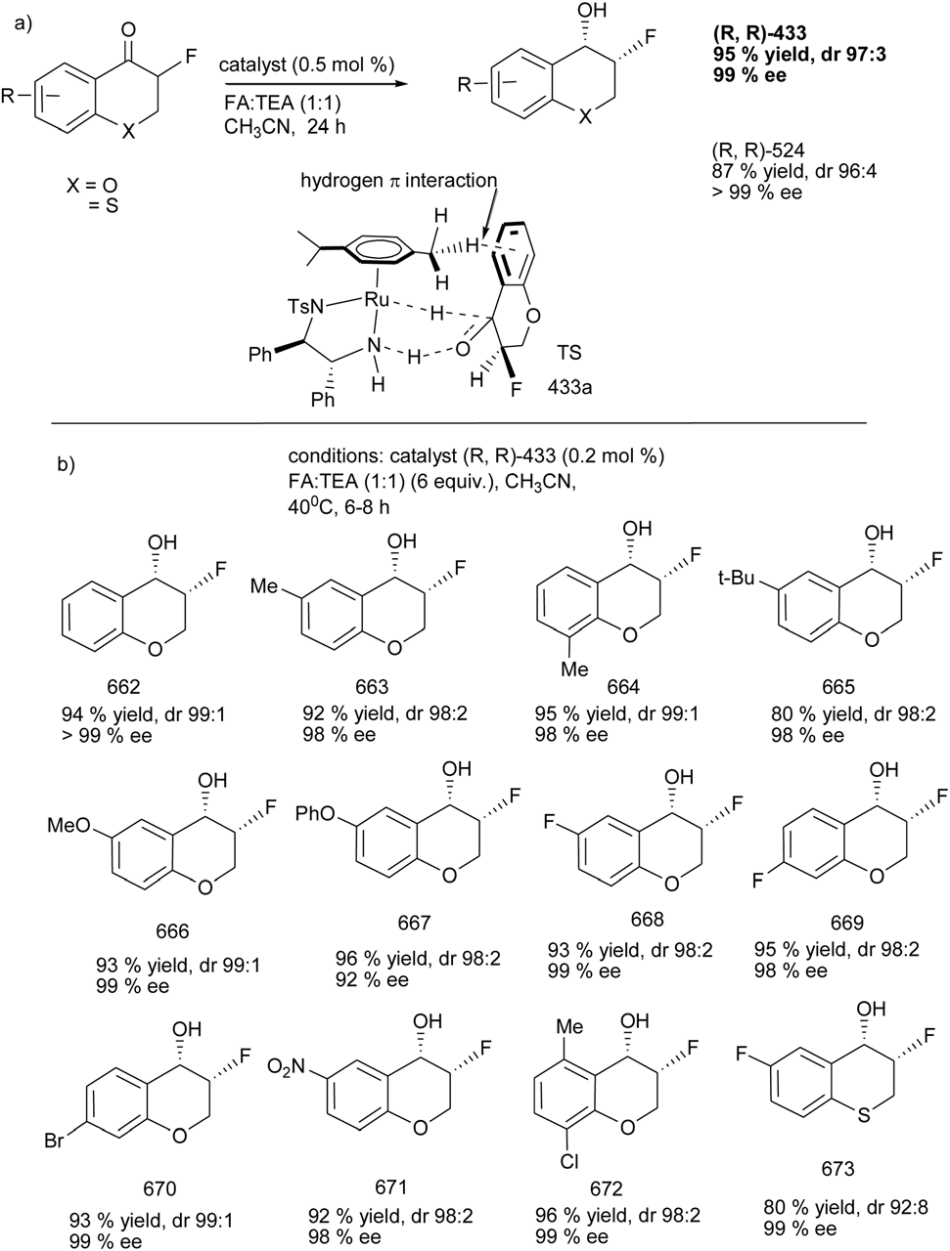
Catalyst used and substrate scope of the ATH/DKR of the 3-fluorochromanone and 3-fluorothiochromanone derivatives. Reproduced from ref. 114 with permission from the American Chemical Society. Copyright 2021.

Based on these results, the catalyst (*R*,*R*)-433 was selected for further reactions (Scheme 36a). Alternative hydrogen source reagents, such as HCO_2_H/DBU (1 : 1) and HCO_2_H/DABCO (1 : 1), were tested in this reaction, resulting in excellent enantioselectivity, although the dr was lower in the former case. Various solvents, including DCM, THF, ^i^PrOH, AcOEt, and toluene, were utilised, but all yielded lower dr compared to acetonitrile. Optimization of hydrogen donor loading showed that 6 equivalents of HCO_2_H/Et_3_N were sufficient to maintain high yield (95%), whereas reducing the amount to 3 equivalents led to a decrease in yield (74%). The optimal conditions were identified as 6 equivalents of HCO_2_H/Et_3_N, S/C = 500, 40 °C, and 6 hours reaction time. A broad range of racemic 3-fluorochroman-4-ones bearing different substituents was then evaluated. Both electron-donating groups (Me, *t*-Bu, OMe, OPh) and electron-withdrawing groups (F, Br, NO_2_, Cl) on the aromatic ring were well tolerated, affording products (663–672) in good to excellent yields (80–96%) with high dr (up to 98 : 2) and excellent ee (92–99%) (Scheme 36b). Additionally, 3-fluorothiochromanone derivatives were also successfully reduced, providing the corresponding *cis*-alcohol 673 in 80% yield with 92 : 8 dr and 99% ee. The absolute configurations of representative products (663 and 672) were confirmed as (3*R*,4*S*) by single-crystal X-ray diffraction. The proposed transition structure (TS-433a) for ATH suggests that a CH–π interaction occurs between the methyl group of the arene and the benzene ring of chromanone, facilitating both hydride transfer from the metal and proton transfer from the ligand to the ketone, resulting in alcohol formation with high ee. The formation of the *cis* isomer is favoured due to the spatial orientation of the ketone and the fluorine being on opposite sides. Finally, a gram-scale reaction was also tested, yielding 98% with a dr of 98 : 2 and >99% ee (662).

**Scheme 37:**
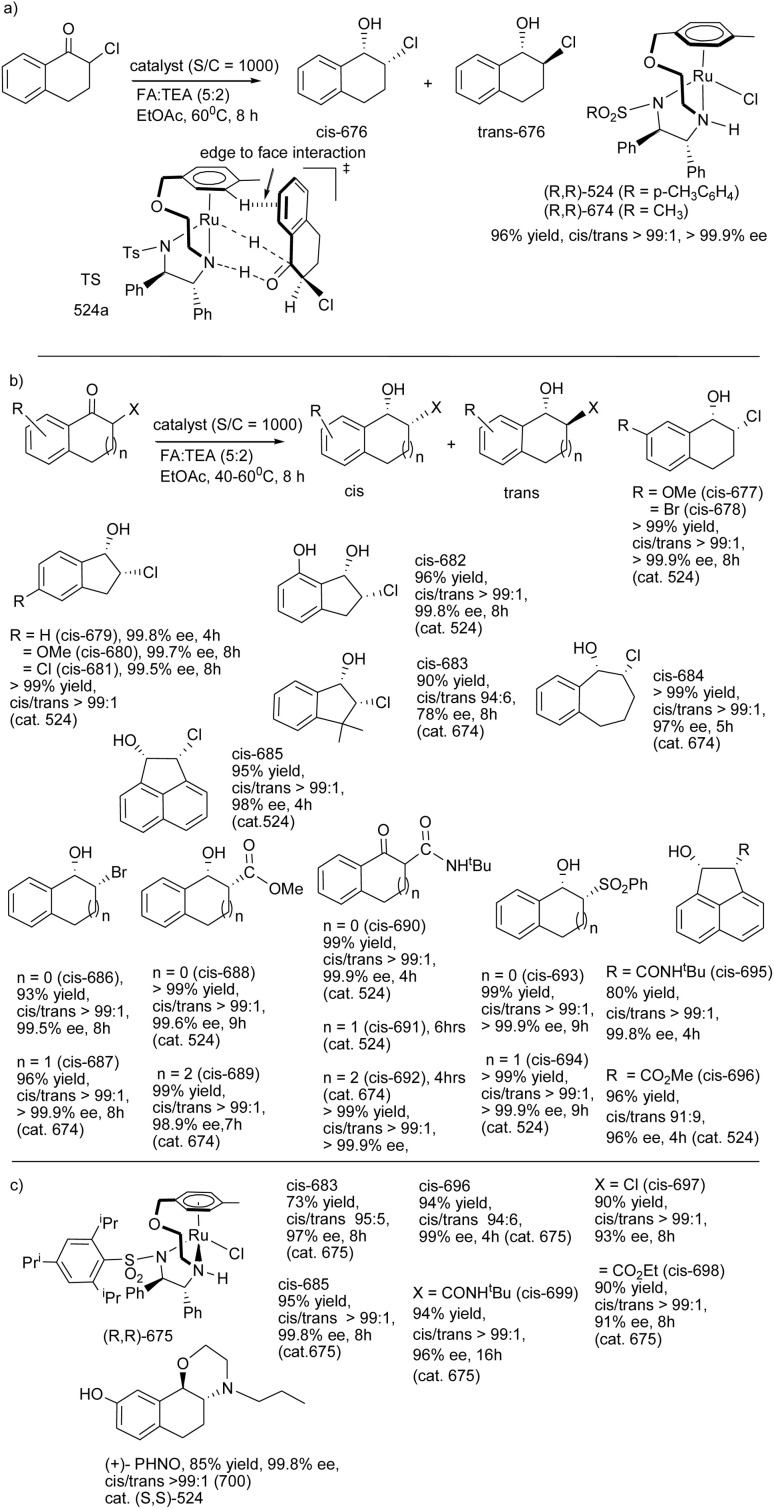
Asymmetric transfer hydrogenation of α-substituted benzocyclic ketones. Reproduced from ref. 115 with permission from the American Chemical Society. Copyright 2021.

Yoshihito Kayaki^115^ (year 2021) reported that an oxo-tethered Ru catalyst efficiently converted α-halogenated ketones to alcohol *via* an ATH-DKR process. The reaction was investigated using racemic 2-chloro-1-tetralone with a 5 : 2 mixture of formic acid (HCOOH) and triethylamine (Et_3_N) as the hydrogen source in ethyl acetate at 60 °C (Scheme 37). Both catalysts (*R*,*R*)-524 and (*R*,*R*)-674 afforded 2-chloro-1-tetralol (*cis*-676) in excellent yield (96%), with outstanding ee (>99.9%) and high dr (*cis*/*trans* >99 : 1). A broad range of α-chlorinated benzocyclic ketones were then examined using catalyst 524. Electron-donating and electron-withdrawing substituents, such as methoxy and bromo groups, were well tolerated, delivering products (*cis*-677 and *cis*-678) in quantitative yield with >99.9% ee and >99 : 1 dr. Under optimized ATH–DKR conditions (substrate-to-catalyst ratio S/C = 200–400, 60 °C, 4–8 h), various substituted 2-chloro-1-indanones were smoothly reduced to the corresponding alcohols (*cis*-679 to *cis*-681) in >99% yield with excellent ee (99.5–99.8%) and dr (>99 : 1). Phenolic (–OH) substituted substrates were also compatible, affording *cis*-682 with high stereocontrol. However, a sterically demanding dimethyl-substituted indanone showed reduced reactivity, giving *cis*-683 in 90% yield and 78% ee when catalyst 674 was used. The methodology was further extended to larger ring systems. A seven-membered benzocyclic α-chloroketone underwent efficient DKR-ATH with catalyst 674, affording *cis*-chlorohydrin 684 in 97% ee. Similarly, acenaphthenone derivatives containing chloro substituents were reduced at 40 °C to give product 685 in 95% yield and 98% ee. α-Bromo ketones also showed excellent reactivity under milder conditions, furnishing products 686 and 687 with high efficiency. A wide variety of functional groups, including esters, amides, and sulfonyl substituents, were well tolerated, providing the corresponding alcohols (688–694) in excellent yields with enantioselectivities up to >99.9% ee using both catalysts 524 and 674. Acenaphthenone derivatives bearing amide and ester groups also reacted efficiently, affording products 695 and 696 in good yields (80–96%) and high ee values (96–99.8%). To further enhance steric control, a bulkier 2,4,6-triisopropylbenzenesulfonamide (TIPPs)-modified catalyst (*R*,*R*)-675 was employed. This catalyst was successfully applied to the substrates dimethyl indanone and acenaphthenones containing chloro and ester, providing yields of 73%, 95%, and 94%, respectively, with enantioselectivities of 97%, 99.8%, and 99% (683, 685, 696). This catalytic method is also applicable for chloro, ester and carboxamide-substituted aliphatic cyclohex-2-ene-1-one compounds, yielding 90–94% with enantioselectivities of 91–96% (697–699). Importantly, (+) PHNO (700) was synthesized using this method; this compound is a dopamine D3 receptor ligand.

**Scheme 38:**
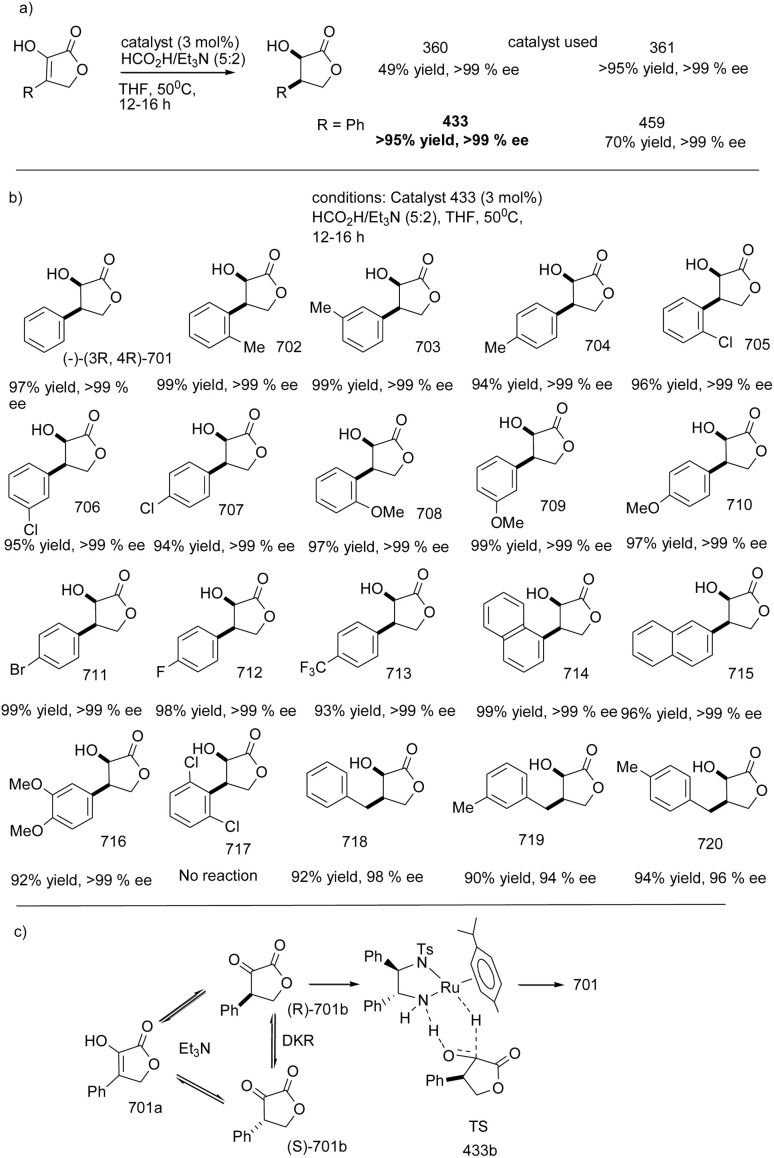
(a) Optimization of Ru-catalyzed ATH. (b) Substrate sacope for the synthesis of α-hydroxybutyrolactones. (c) Proposed possible catalytic mechanism. Reproduced from ref. 116 with permission from the American Chemical Society. Copyright 2021.

Yong Zhou *et al.*^116^ (year 2021) reported the synthesis of substituted α-hydroxybutyrolactones from substituted α-oxobutyrolactones using a ruthenium catalyst. This ATH process occurs *via* DKR, leading to the formation of two contiguous stereogenic centres. 3-hydroxy-4-phenylfuran-2(5*H*)-one was reduced by catalyst 433 in the presence of an azeotropic mixture of formic acid and triethylamine, yielding the reduced product (701) with over 99% ee and a dr of more than 20 : 1 (Scheme 38a). Various solvents, including ethyl acetate, THF, toluene, DCE, and DMF, were evaluated, among which THF provided the best catalytic performance. A set of ruthenium catalysts (360, 361, 433, 459) was also screened and showed comparable high activity and excellent enantioselectivity; however, catalyst 433 was selected for further studies due to its cost-effectiveness and ready availability. A broad range of β-substituted α-oxobutyrolactones bearing substituents such as Me, Cl, and OMe at different positions of the aromatic ring were successfully reduced, giving excellent yields (94–99%) and uniformly high ee (>99% ee) (702–710) (Scheme 38b). Substrates containing electron-withdrawing groups at the *para* position also performed efficiently, affording products in 93–99% yield with >99% ee (711–713). Naphthyl-substituted substrates (1- and 2-naphthyl) were well tolerated, delivering excellent outcomes in both yield and stereoselectivity (714–715). Substrates bearing methoxy groups at the 3- and 4-positions also showed excellent reactivity (716). In contrast, the 2,6-dichlorophenyl-substituted substrate (717) failed to give product formation, likely due to steric congestion around the reactive site. Benzyl-substituted derivatives also underwent smooth transformation, providing products in 90–94% yield with 94–98% ee (718–720). A gram-scale reaction further confirmed the robustness of the protocol, affording the product in 94% yield with >99% ee. Mechanistically, the reaction is proposed to proceed *via* initial tautomerization of substrate 701a to form *R*-701b and *S*-701b under DKR conditions. The reactive *R*-isomer then undergoes ATH in the presence of the chiral ruthenium catalyst. The stereochemical outcome is controlled by a well-defined transition state in which favorable alignment between the substrate and the catalyst minimizes steric repulsion and directs hydride/proton transfer to furnish the final product 701 with high stereocontrol (Scheme 38c).

**Scheme 39:**
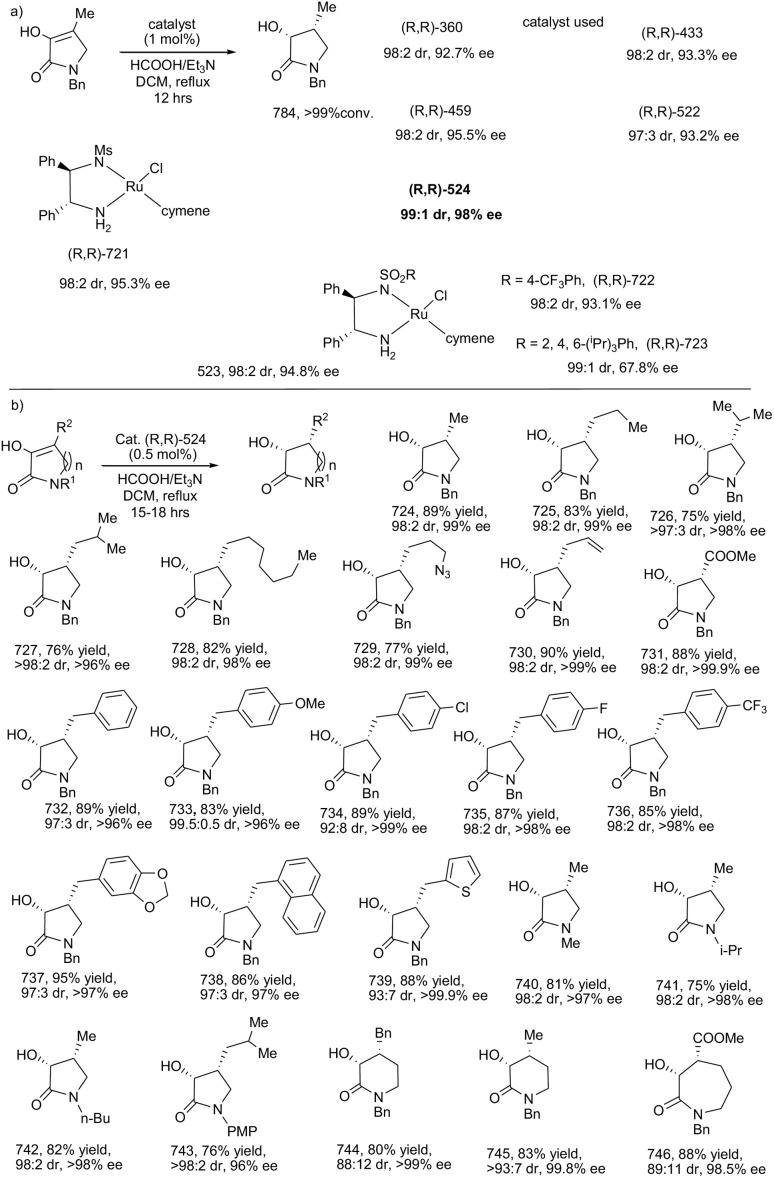
(a) Evaluation of chiral catalysts. (b) Substrate scope of DKR-ATH for α, β-disubstituted-lactams. Reproduced from ref. 117 with permission from Wiley-VCH. Copyright 2021.

Z. Wang *et al.*^117^ (2021) reported the synthesis of α, β-disubstituted lactams *via* an ATH–DKR strategy using various ruthenium catalysts. The racemic substrate, 1-benzyl-4-methylpyrrolidine-2,3-dione, was used to evaluate catalytic performance. All the tested catalysts achieved full conversion (>99%), while (*R*,*R*)-522 and Noyori's catalyst (*R*,*R*)-433 showed particularly high efficiency (Scheme 39a). A series of sulfonamide-based catalysts [(*R*,*R*)-721, (*R*,*R*)-459, (*R*,*R*)-722, (*R*,*R*)-523] delivered products with enantioselectivities up to 95.5% ee. In contrast, catalyst (*R*,*R*)-723 gave a lower ee (67.8%), which was attributed to steric hindrance from its isopropyl substituent, reducing catalytic efficiency. Tethered ruthenium catalysts [(*R*,*R*)-524 and (*R*,*R*)-360] were also investigated; catalyst 360 afforded 92.7% ee with a diastereomeric ratio of 98 : 2, while catalyst 524 provided the product with an excellent 98.7% ee. A broad range of 4-substituted *N*-benzyl pyrrolidine-2,3-diones was then explored under optimized ATH–DKR conditions (Scheme 39b). Variations in alkyl substituents at the 4-position were well tolerated, giving products with excellent ee (up to 99%, compounds 724–728). Only compound 727 showed a slightly reduced ee (96%). Functional groups such as azido, alkenyl, and ester substituents were also compatible, consistently delivering products with 99% ee (729–731). Aryl and heteroaryl methylene substituents, including phenyl, substituted phenyl, 1-naphthyl, and 2-thienyl groups, were smoothly reduced to give high yields and excellent enantioselectivities (732–739). Slightly lower regioselectivity was observed for compounds 734 and 739 (92 : 8 and 93 : 7, respectively).

**Scheme 40:**
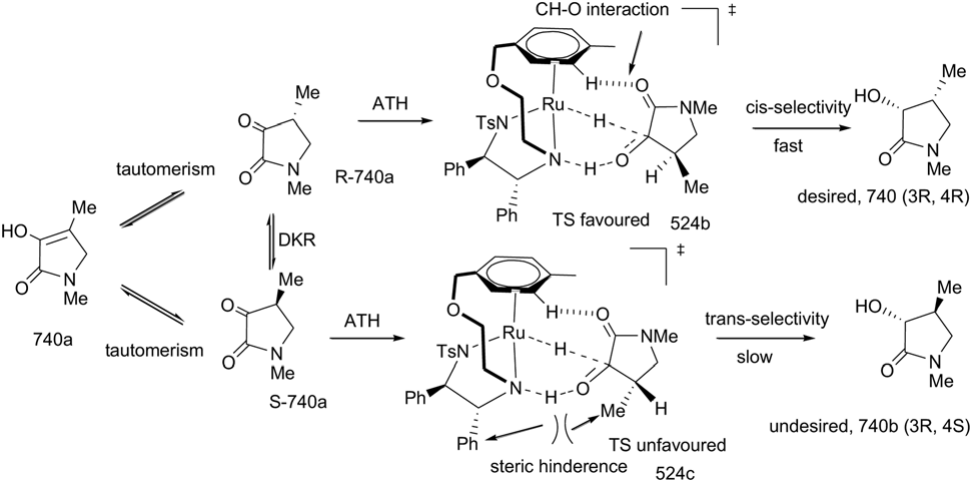
Proposed catalytic mechanism.

The absolute configuration of compound 736 was confirmed by single-crystal X-ray diffraction. Additionally, modification of the *N*-benzyl group with substituents such as Me, ^*i*^Pr, *n*-Bu, and PMP was well tolerated, affording products with good to excellent regio- and enantioselectivity (740–743). Six- and seven-membered ring substrates were also compatible, providing products with high enantioselectivity, although with a slight decrease in dr (744–746). The proposed mechanism (Scheme 40) suggests that the enol form of the substrate (740a) undergoes tautomerization to both *R*- and *S*-isomers. The *R*-isomer reacts preferentially with the Ru–H species *via* a favorable six-membered transition state (TS-524b), stabilized by a C–H⋯O interaction between the catalyst's arene and the substrate oxygen, leading to the expected product 740. In contrast, the *S*-isomer proceeds through a less favorable transition state (TS-524c), where steric repulsion between the catalyst and substrate destabilizes the pathway, resulting in disfavored product formation (740b).

**Scheme 41:**
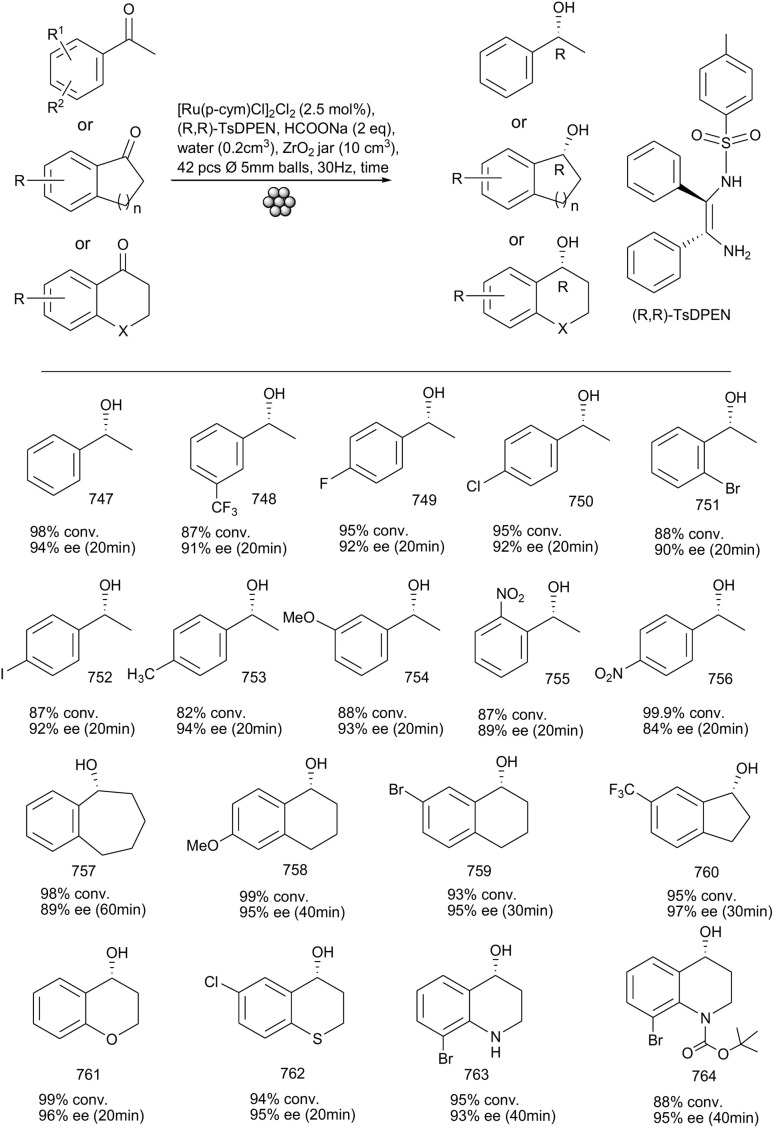
Mechanochemical ATH of acetophenone derivatives, carbocyclic ketones, heterocyclic ketones. Reproduced from ref. 118 with permission from Wiley-VCH. Copyright 2022.

The G. Szőllősi group^118^ (2022) demonstrated a sustainable and environmentally friendly mechanochemical ATH process using the Noyori–Ikariya Ru–TsDPEN catalyst in an aqueous medium with HCOONa. They examined the ATH reaction of acetophenone using a ball milling method, optimising the number and size of milling balls, milling frequency, and milling time. The optimal conditions were found to be 42 balls with a diameter of 5 mm at a frequency of 30 Hz for 15 minutes. In this mechanochemical process, a range of acetophenone derivatives as well as carbo- and heterocyclic ketones were reduced, resulting in high enantioselectivity and conversion within a short time (Scheme 41). In contrast, the same reaction under magnetically stirred batch conditions required significantly more time, as reported previously. The reduction of aryl rings with both electron-donating and electron-withdrawing groups yielded excellent conversion and enantioselectivity (747–756). Additionally, aliphatic cyclic ketones fused with aromatic rings were also reduced with good conversion and very high enantioselectivity, though the reaction took slightly longer compared to that of acetophenone (757–760). Numerous heterocyclic ketones were processed successfully; 4-chromanone and 4-thiochromanone smoothly reduced with very good conversion (761–764). N-heterocyclic ketones were performed well delivering the products with very good conversion, but this took a bit longer (40 minutes) due to the presence of a bulky bromine substituent and a large *tert*-butoxycarbonyl (Boc) group protecting the nitrogen, which inhibited coordination with the metal and suppressed reactivity. After scaling up the reaction, a 10 cm^3^ grinding jar was found to be sufficient for the reduction of 8 mmol of ketone. Notably, 6-methoxytetralone was not reduced under batch conditions; however, the mechanochemical process successfully achieved the reduction, resulting in a good conversion and very good ee (97% yield, 94% ee, 2 hours).

**Scheme 42:**
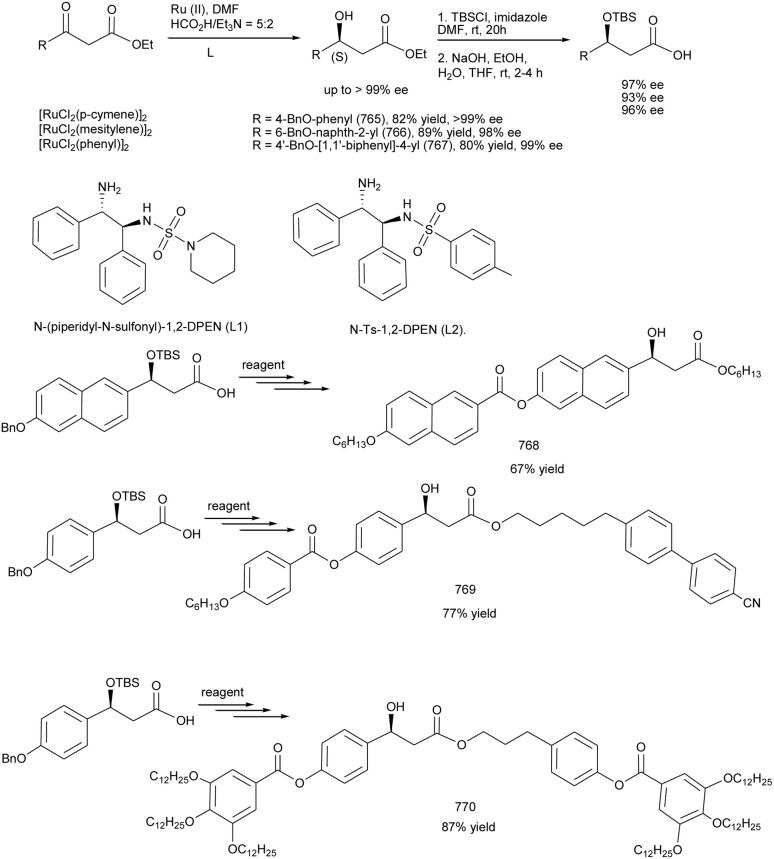
Ruthenium catalyzed ATH reaction and protection of hydroxyl group and synthesis of LC. Reproduced from ref. 119 with permission from the American Chemical Society. Copyright 2022.

Andreja Lesac *et al.*^119^ (2022) reported the synthesis of 3-aryl-3- hydroxypropanoic esters (aryl = phenyl, naphthyl, biphenyl) *via* a Ru(ii)-catalyzed ATH process (Scheme 42). These compounds serve as important chiral building blocks for liquid crystalline (LC) materials, enabling the construction of rod-shaped, bent-shaped, flexible dimeric, and polycatenar architectures exhibiting distinct mesomorphic properties. The ATH reaction was carried out using dimeric precatalysts [RuCl_2_(η^6^-arene)]_2_ (arene = *p*-cymene, mesitylene, or benzene) in combination with chiral ligands such as *N*-(piperidyl-*N*-sulfonyl)-1,2-diphenylethylenediamine (L1) and N-Ts-1,2-DPEN (L2). Under optimized conditions, excellent levels of stereocontrol were achieved. For the phenyl-substituted substrate, the use of 5 mol% [RuCl_2_(η^6^-mesitylene)]_2_ with ligand L2 at room temperature afforded the corresponding alcohol in 82% yield with >99% ee (compound 765). Naphthyl and biphenyl derivatives showed equally impressive performance under milder catalyst loading (2 mol%), giving products with 89% and 80% yields and 98% and 99% ee, respectively (766–767). To access the desired chiral intermediates, the hydroxy group was initially protected using *tert*-butyldimethylsilyl chloride (TBSCl), followed by hydrolysis to generate the corresponding acids. These intermediates were further coverted into advanced LC architectures. Notably, the rod-shaped material (768) was synthesized in 67% overall yield, while the flexible bent-shaped dimer (769) was obtained with 99% ee. A more complex hexacatenar bent-shaped dimer (770) was successfully prepared in 87% yield. Importantly, compounds 768 and 769 exhibited TGBA and BPIII mesophases, confirming that these chiral 3-aryl-3-hydroxypropanoic esters are effective functional units for liquid crystalline materials. In general, this work explored the utility of ATH-derived chiral alcohols as multifunctional building blocks for the design of advanced soft materials with potential applications in optoelectronic and display technologies.

**Scheme 43:**
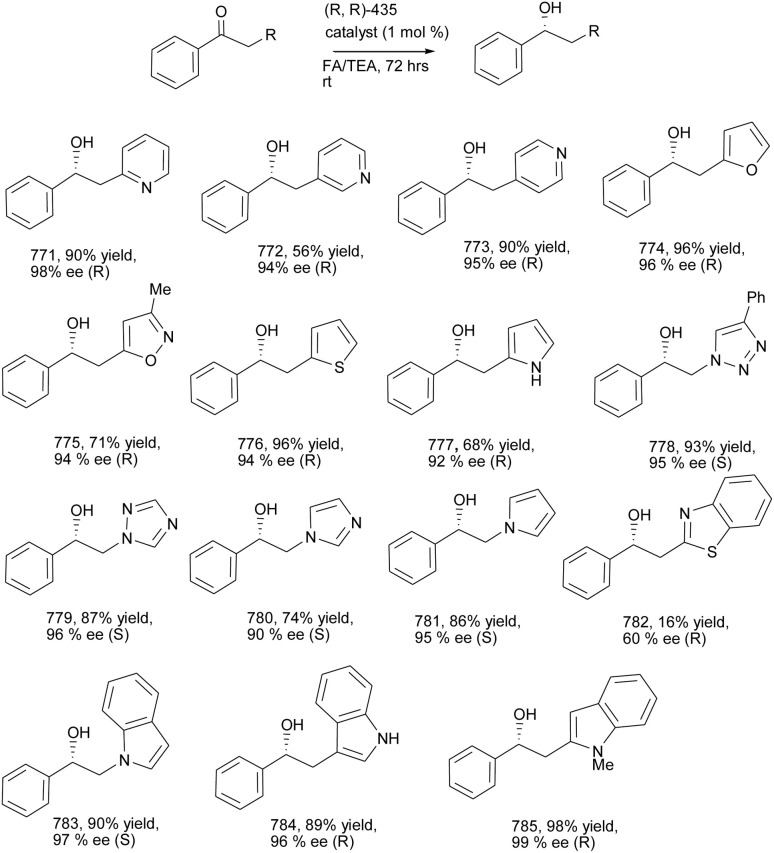
ATH products from heterocyclic ketones. Reproduced from ref. 120 with permission from Elsevier. Copyright 2022.

Martin Wills *et al.*^120^ (2022) reported the ATH of a range of heterocyclic acetophenone derivatives using an N-functionalised [(benzene)Ru(TsDPEN)] catalyst, [(*R*,*R*)-435]. The reactions were carried out using a formic acid/triethylamine (FA/TEA) system as the hydrogen source, providing chiral heteroaryl alcohols with generally high yields and excellent enantioselectivities (Scheme 43). Pyridyl-substituted ketones were efficiently reduced: 2-pyridyl, 3-pyridyl, and 4-pyridyl derivatives afforded the corresponding alcohols (771–773) in 90%, 56%, and 90% yield with enantioselectivities of 98%, 94%, and 95%, respectively. Among oxygen-containing heterocycles, furan and isoxazole substrates also performed well, giving products 774 and 775 in 96% yield (96% ee) and 71% yield (94% ee), respectively. Sulfur- and nitrogen-containing heterocycles were similarly well tolerated. Thiophene and pyrrole derivatives furnished alcohols 776 and 777 in 96% yield (94% ee) and 68% yield (92% ee), respectively. Triazole-based substrates, including substituted and fused variants, also underwent smooth reduction, providing products 778 and 779 in good yields (87–93%) with high ee (95–96%). Imidazole and pyrrole-containing ketones afforded alcohols 780 and 781 in 74% (90% ee) and 86% (95% ee) yield, respectively. More challenging heterocycles were also investigated. Benzothiazole-containing substrates showed reduced reactivity, delivering product 782 in only 16% yield with moderate ee (60%), which was attributed to poor solubility under the reaction conditions. In contrast, indole-containing ketones were efficiently reduced, giving products 783–785 in excellent yields (89–98%) with outstanding enantioselectivities (96–99% ee). Overall, this study highlights the broad applicability of the Noyori–Ikariya type ruthenium catalyst system for the enantioselective reduction of structurally diverse heteroaromatic ketones, demonstrating both functional group tolerance and high stereocontrol.

**Scheme 44:**
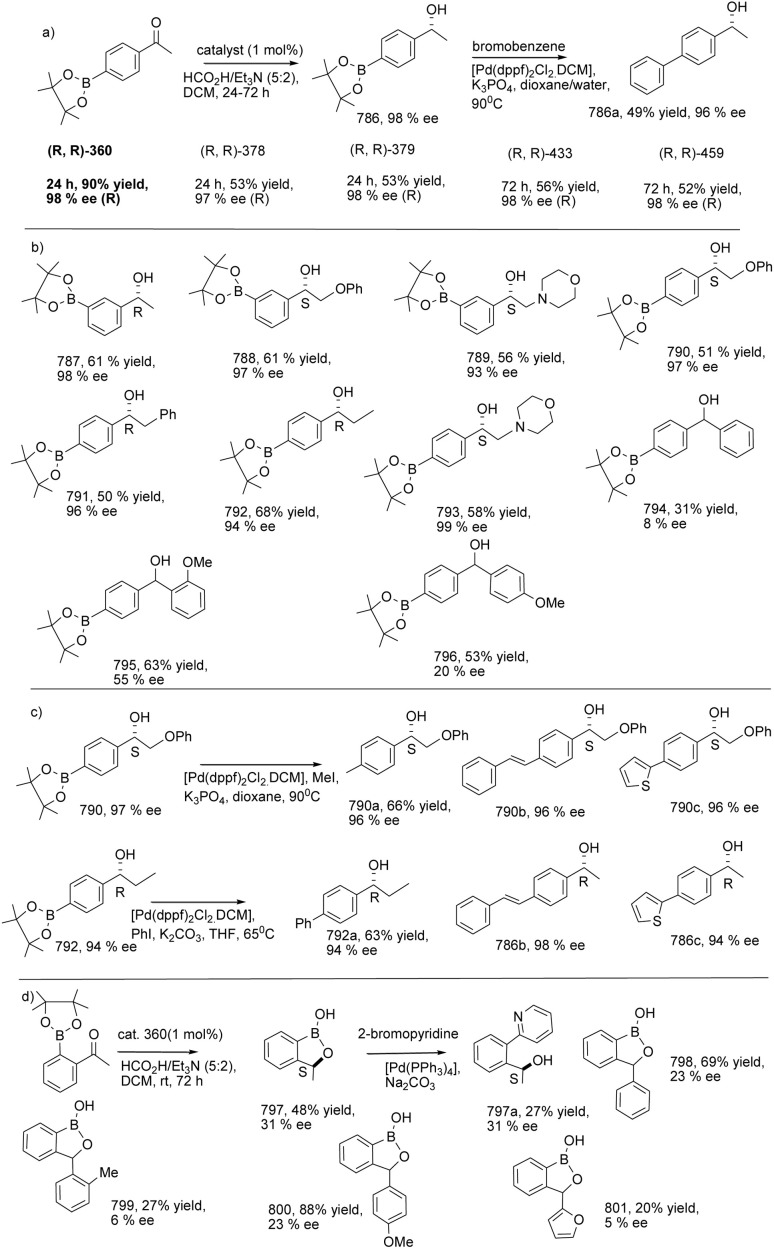
(a) Catalysts used in this study. (b) ATH products of Bpin-containing ketones. (c) Coupling products and ATH products. (d) ATH of *ortho*-Bpin acetophenone and Bpin-containing aromatic rings ketones. Reproduced from ref. 121 with permission from the Royal Society of Chemistry. Copyright 2022.

Wills *et al.*^121^ (2022) reported the ATH of *para*-substituted Bpin-containing acetophenones using a ruthenium catalyst in the presence of a 5 : 2 azeotrope of formic acid/triethylamine (FA/TEA) in dichloromethane. The transformation afforded the corresponding chiral alcohols in good to excellent yields with high ee. A series of Noyori–Ikariya type ruthenium catalysts (360, 378, 379, 433, 459) were evaluated (Scheme 44a), among which the 3C-tethered catalyst (*R*,*R*)-360 proved to be the most efficient. Under optimised conditions, this catalyst furnished product 786 in 90% yield with 98% ee after 24 hours. The resulting Bpin-containing alcohol was further functionalized *via* Pd-catalysed cross-coupling with bromobenzene to give the corresponding 4-phenyl derivative (786a). Importantly, both *para*- and *meta*-Bpin-substituted substrates were fully converted to the corresponding alcohols without loss of ee. The substrate scope was further expanded to α-substituted ketones bearing Me, Ph, phenoxy, and morpholine groups, all of which were smoothly reduced to the corresponding alcohols with excellent conversions and high enantioselectivities (93–99% ee, compounds 787–793) (Scheme 44b). In contrast, sterically demanding *ortho*-Bpin-substituted substrates behaved differently: reduction of such a substrate led to cyclisation, forming a benzoboroxole derivative (797) with significantly lower ee (31%) (Scheme 44d). Further studies revealed a strong influence of both electronic and steric effects on enantioselectivity. For example, replacement of the Bpin group with phenyl or methyl substituents in substrates 790 and 792, followed by Pd-catalysed coupling, furnished alcohols 790a and 792a with excellent enantioselectivities of 96% and 94%, respectively. However, a *para*-Bpin substrate bearing a phenyl substituent gave only 8% ee (794), while *ortho*- and *para*-methoxy substituents resulted in reduced ee (55% for *ortho*-OMe, 20% ee for *para*-OMe), likely due to steric and electronic perturbations (795–796). Additionally, *ortho*-Bpin-substituted aromatic ketones tended to undergo intramolecular cyclisation during reduction, giving cyclic products 798–801 with generally poor ee. Finally, the synthetic utility of this methodology was demonstrated through subsequent Pd-catalysed cross-coupling reactions with 2-bromostyrene and 2-bromothiophene, affording coupled products (786b, 786c, 790b, and 790c) without erosion of stereochemical integrity.

**Scheme 45:**
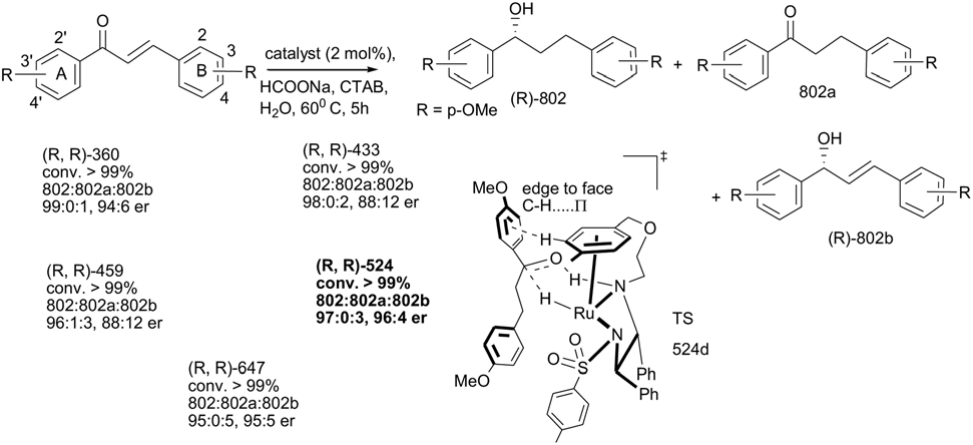
Screening of catalysis of the ATH of enones. Reproduced from ref. 122 with permission from the American Chemical Society. Copyright 2022.

**Scheme 46:**
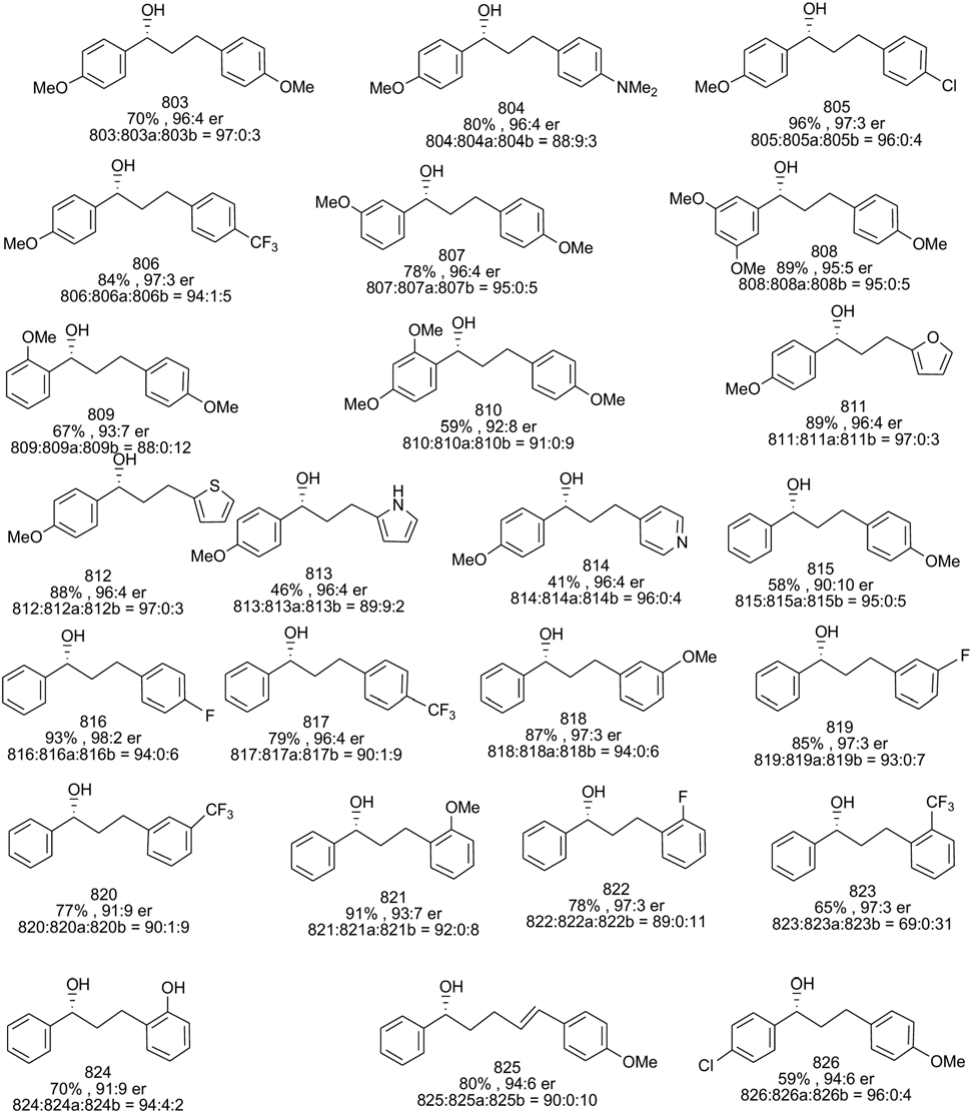
Scope of the ATH of enones by catalyst (*R*,*R*)-524. Reproduced from ref. 122 with permission from the American Chemical Society. Copyright 2022.

P. R. R. Costa^122^*et al.* (2022) reported the reduction of chalcone using a tethered Ru(ii) Noyori–Ikariya catalyst ((*R*,*R*)-360) with HCO_2_Na and CTAB in a 1 : 1 MeOH/H_2_O mixture at 40 °C for 5 hours (Scheme 45). The reaction afforded 1,3-diarylpropan-1-ol (802, 59%) as the major product, along with dihydrochalcone (802a, 37%) and a minor amount of allylic alcohol (802b, 4%), with an overall product ratio of 99 : 0 : 1. When methanol was used as the sole solvent, the overall yield increased, but dihydrochalcone (802a) became the dominant product. In contrast, using water as the solvent reduced conversion, although 802 remained the major product. Increasing the temperature to 60 °C led to complete conversion (>99%) and exclusively formed product 802 with improved er (94 : 6). Several Noyori–Ikariya type catalysts, including (*R*,*R*)-433, (*R*,*R*)-459, (*R*,*R*)-647, and (*R*,*R*)-524, were evaluated. All catalysts showed high activity and full conversion; however, catalysts 433 and 459 bearing a *p*-cymene ligand exhibited reduced stereoselectivity (88 : 12 er). In contrast, the mesitylene-based catalyst (*R*,*R*)-647 improved stereo-control and primarily furnished product 803 with only a minor amount of 802a (5%), attributed to stabilizing C–H⋯π interactions between the mesitylene ring and the substrate. Notably, the oxo-tethered Ru(ii) catalyst (*R*,*R*)-524 delivered the best stereoselectivity (96 : 4 er), along with only 3% formation of side product 802b. The enhanced performance of 524 is attributed to the rigid tethering between the arene and diamine ligands, which improves both catalytic efficiency and stereo-discrimination. Using catalyst 524, a broad range of chalcone derivatives was investigated (Scheme 46). Variations in the B-ring substituents, including electron-donating and electron-withdrawing groups, were well tolerated, affording 1,3-diarylpropan-1-ols (*R*)-803–806 in good to excellent yields (70–96%) and high enantioselectivities (96 : 4 to 97 : 3 er). Electron-donating substituents such as 4-NMe_2_ led to slightly higher formation of side products (804a, 9%), whereas electron-withdrawing groups such as 4-Cl and 4-CF_3_ improved selectivity toward the desired alcohols (805–806, 84–96% yield). Substituents on the A-ring (OMe at 3′ and 5′ positions) also showed strong reactivity, providing products 807 and 808 with 78–89% yield and 95 : 5–96 : 4 er. However, substitution at the 2′-position slightly reduced enantioselectivity due to steric effects, accompanied by a minor increase in side products (9–12%). Heteroaryl-substituted chalcones were also compatible with the reaction. Due to the production of an 813a product of 9%, the enone compound containing pyrole exhibited the lowest reactivity (46% yield), whereas the enone compound having furan exhibited the highest reactivity (89% yield). By changing the *ortho*, *meta*, and *para* substituents in ring B with the OMe, F, and CF_3_ groups, the stereoelectronic effect was evaluated. In the *para* and *meta* positions, chalcone with electron-withdrawing groups –F and –CF_3_ were reduced to produce the enol product (816–820) with a small amount of 1,4 selectivity product; in the enol product, the er value slightly increased. Allylic alcohol and the enol derivative (821–823) were formed upon reduction of chalcone containing electron-withdrawing groups such as –OMe, –F, and –CF_3_. In this case, the alcohol product increased compared to the *para* and *meta* position isomers due to the steric hindrance. This impact was more pronounced for the chalcone-containing –CF_3_ group (823 : 823b = 69 : 31). The –OH containing B ring produced a 70% yield and an er of 91 : 9 (824), whereas the –Cl containing A ring gave a 59% yield (826) and an er of 94 : 6. Alcohol was produced with a good yield (825, 80%) and er 94 : 6 by reducing the carbon–carbon double bond between the A and B rings substrate. The 2′-hydroxy-4-methoxychalcone showed faster reactivity than 4-methoxychalcone, but the er value dropped (73 : 27). However, the er value increased (95 : 5) when the reaction took place at room temperature due to the hydrogen bond between the carbonyl oxygen and OH hydrogen. The C–H⋯π interaction between the arene ligand and substrate in Scheme 45 (TS-524d) explains the stereoselectivity of the 524 catalysts; the arene ligand and the electron-rich (4′-OMe) phenyl ring have a strong interaction that favours the higher er value. Moreover, a gram-scale ATH reaction was conducted in water using a 16-fold substrate (1.07 gm) and a 10-fold lowered catalyst (0.2 mol%) under the same conditions. The product was obtained with a 66% yield and >99 : 1 er (803, recrystallisation). Using this ATH condition, (*S*)-BW683C was synthesized with an 85% yield and 90 : 10 er; likewise, (*S*)-tephrowatsin *E* was synthesized with 92 : 8 er and 62% yield.

**Scheme 47:**
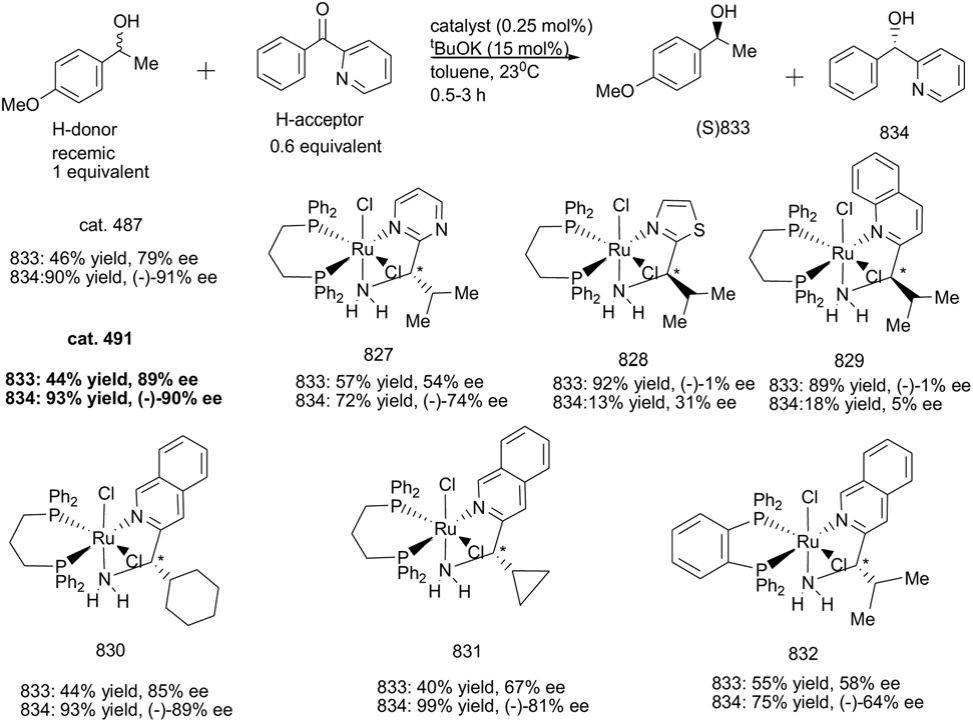
Identification of the optimal catalyst. Reproduced from ref. 123 with permission from the American Chemical Society. Copyright 2022.

**Scheme 48:**
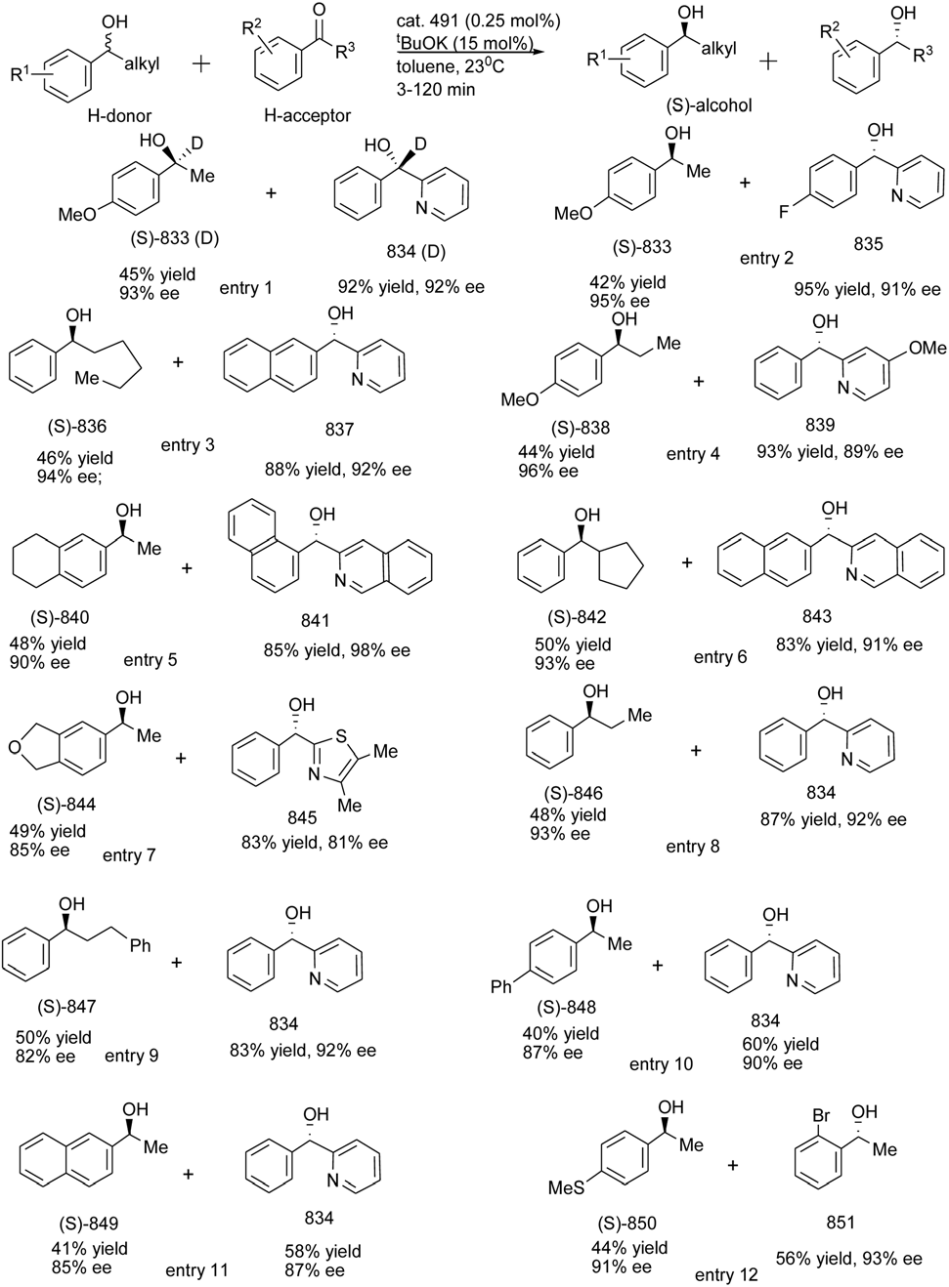
Substrate scope for simultaneous kinetic resolution of racemic alcohols and asymmetric reduction of matching ketones *via* one-pot cross transfer hydrogenation. Reproduced from ref. 123 with permission from the American Chemical Society. Copyright 2022.

Applying a ruthenium chiral catalyst, Xiangyou Xing^123^ (year 2022) reported cross-ATH between the reaction of H-acceptor prochiral ketone and H-donor racemic alcohols, leading to the formation of two enantio-enriched alcohols. Electron-withdrawing groups in aryl ketones, which are more electrophilic and better hydride acceptors, and easy H-transfer between the H-donor and the H-acceptor occur when the equilibrium constant *K* value is large. This reaction was carried out with α-methyl-4-methoxybenzyl alcohol as the H-donor, while phenyl 2-pyridiyl ketone was used as the H-acceptor. Various ruthenium catalysts were employed in Scheme 47 to assess the enantioselectivity of the substrate. Utilising catalyst 487 in this reaction resulted in a 46% yield of 833 (79% ee), while compound 834 exhibited excellent selectivity (91% ee, 90% yield). The more electron-deficient pyrimidine ring in the diamine ligand of catalyst 827 results in 833 exhibiting a 54% ee and hydrogenated 834 showing a 74% ee, which is lower than that of catalyst 487. Interestingly, catalysts with bulkier quinoline (catalyst 829) and electron-rich thiazole (catalyst 828) demonstrated significantly lower selectivity. In contrast, catalyst 491, which features an isoquinoline ring, showed outstanding selectivity for hydrogenated 833 and 834. Modification of the stereogenic center substituent in the diamine ligand also influenced performance. Catalyst 830, bearing a cyclohexyl group, showed kinetic resolution and asymmetric induction comparable to catalyst 829. However, the cyclopropyl-substituted catalyst 831 delivered lower selectivity (67% ee for kinetic resolution and 81% ee for asymmetric induction). Additionally, incorporation of achiral diphosphine 1,2-bis(diphenylphosphanyl)benzene (832) led to a noticeable decline in both kinetic resolution efficiency and enantioselectivity. Based on these results, catalyst 491 was identified as the most effective system and was subsequently applied to a broad range of substrates (Scheme 48). Various aryl and heteroaryl ketones, including 4-fluorophenyl-2-pyridyl, naphthyl-pyridyl, methoxy-substituted pyridyl, and thiazolyl derivatives, were successfully converted. A wide range of alcohols was also employed as hydrogen donors, including both electron-rich and electron-neutral aryl–alkyl alcohols as well as alkyl alcohols of varying steric demand.

Substituent effects were also evident: for example, an *ortho*-bromo substituent on the aryl ring suppressed product dehydrogenation, thereby directing the reaction pathway and enhancing selectivity. Notably, the system enabled access to both enantiomers of the products depending on the reaction sequence and hydrogen source employed. In time-course studies using 1 mol% catalyst 491 and phenyl 2-pyridyl ketone, the ee of (*R*)-834 remained consistently high (96.5% after 5 s and 99.5% after 1.5 h). Upon addition of ^*i*^PrOH to the reaction mixture as an H-donor, a decrease in the ee of the opposite enantiomer (*R*)-833 was observed over time (ee at 2 hours = 91%, ee at 15 hours = 5%). The resulting alcohols have an opposite stereochemistry, produced through kinetic resolution and asymmetric reduction. Various aryl–alkyl substituted (*R*)-enantiomers were synthesised by reacting racemic alcohols with H-acceptor 2-pyridiyl ketone, followed by the addition of excess H-donor isopropanol (^*i*^PrOH). Interestingly, (*S*)-enantiomers of compounds 833, 847, 849, and 850 were produced through kinetic resolutions. This was followed by a sequential TH process, which yielded the corresponding (*R*)-enantiomers. This cross-TH reaction successfully resolves racemic alcohols and asymmetrically reduces ketones, providing an elegant one-step method for the production of enantiomeric alcohols.

**Scheme 49:**
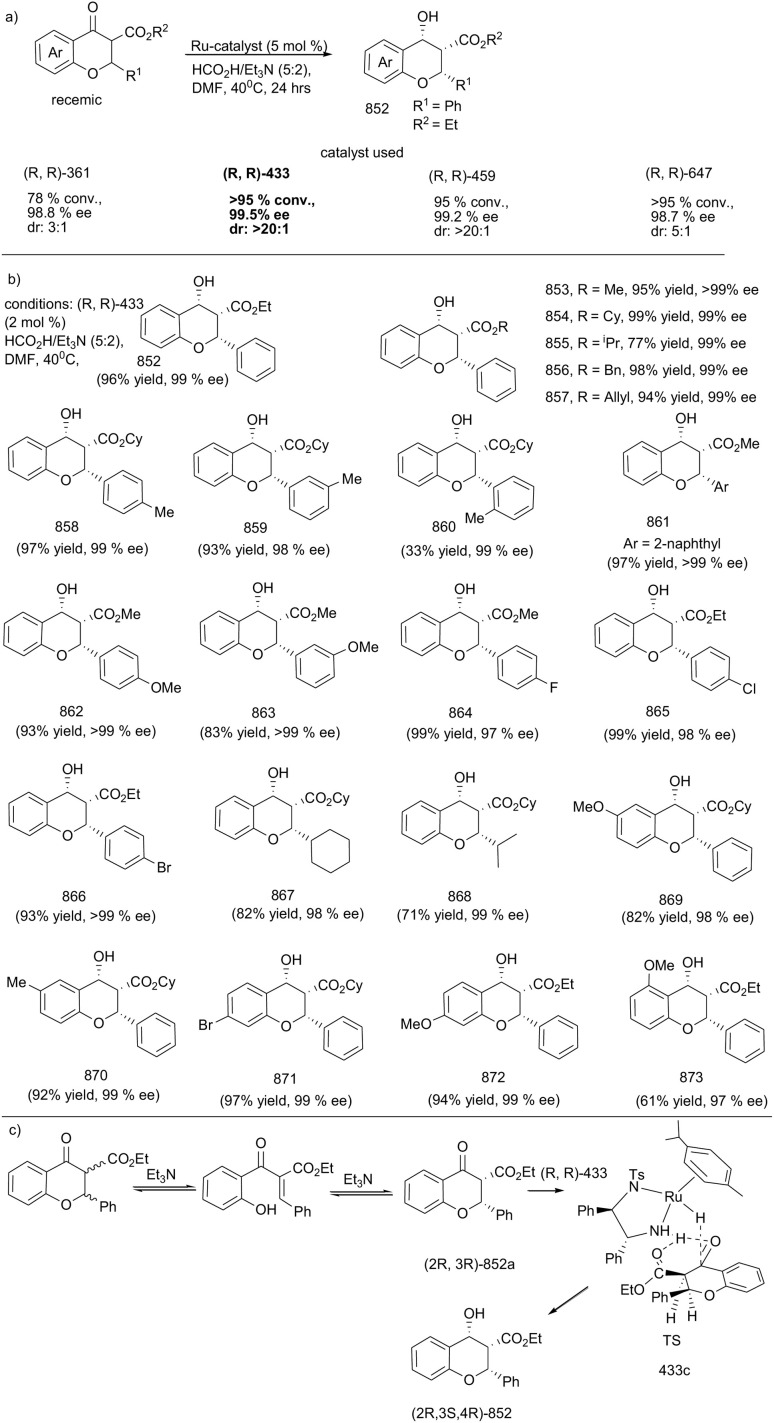
(a) Catalyst screening. (b) Substrate scope for the synthesis of chiral 2,3-disubstituted flavanols. (c) Proposed reaction mechanism. Reproduced from ref. 124 with permission from the American Chemical Society. Copyright 2022.

Yong-Gui Zhou *et al.*^124^ (2022) reported a ruthenium-TH of readily available 2,3-disubstituted flavanones, providing access to enantioenriched flavanols with excellent ee and dr. This transformation proceeds *via* a DKR process and constructs three contiguous stereogenic centers in a single catalytic operation. Initially, the ATH of 2,3-disubstituted flavanones was performed using the (*R*,*R*)-433 catalyst in ethyl acetate, affording the corresponding flavanol in 83% yield with 94% ee (Scheme 49a). A range of solvents, including DCM, THF, toluene, and DMF, was screened to optimise the reaction condition, with DMF giving the best overall performance. Among related catalysts, (*R*,*R*)-647 provided comparable activity but reduced dr, whereas (*R*,*R*)-459 delivered both excellent ee and dr. In contrast, catalyst (*R*,*R*)-361 maintained high ee but afforded only moderate dr (3 : 1). Under optimised conditions (2 mol% (*R*,*R*)-433, HCO_2_H/Et_3_N (5 : 2), DMF, 40 °C), a wide range of flavanone derivatives was successfully reduced. Substrates bearing various ester groups such as ethyl, methyl, and cyclohexyl afforded products 852–854 in excellent yields (95–99%) with outstanding ee (up to 99%). Other esters including isopropyl, benzyl, and allyl also performed well, giving good yields (77–98%) while maintaining excellent ee values (855–857, ≈99% ee). Substituent effects on the aromatic ring were also investigated. Cyclohexyl ester derivatives bearing *ortho*-methyl substitution showed a significant decrease in yield (33%) due to steric hindrance, although ee remained excellent (99%, 860). In contrast, *meta*- and *para*-methyl substituted analogues delivered high yields (93–97%) with excellent ee (858, 859, 98–99%). A 2-naphthyl-substituted substrate afforded 861 in 97% yield with >99% ee. Electron-donating groups (OMe) at *ortho* and *meta* positions gave good yields (83–93%) with excellent ee (>99%), while electron-withdrawing substituents (F, Cl, Br) at the *para* position were also well tolerated, providing high yields (93–99%) and excellent ee (97–99%). Aliphatic substituents such as cyclohexyl and isopropyl were likewise compatible, affording products with good yields and high ee. Further variation of multi-substituted aromatic systems (869–873) demonstrated consistently high ee, although slight yield reduction was observed in the *ortho*-substituted case due to steric effects. The methodology was also successfully demonstrated on a gram scale, delivering the product in 95% yield with 99% ee.

Mechanistically, the reaction is proposed to proceed through racemisation of flavanones followed by enantioselective reduction. The key stereodetermining step involves hydride and proton transfer through a well-defined transition state (TS-433c), leading to the formation of the (2*R*,3*S*,4*R*)-flavanol product.

**Scheme 50:**
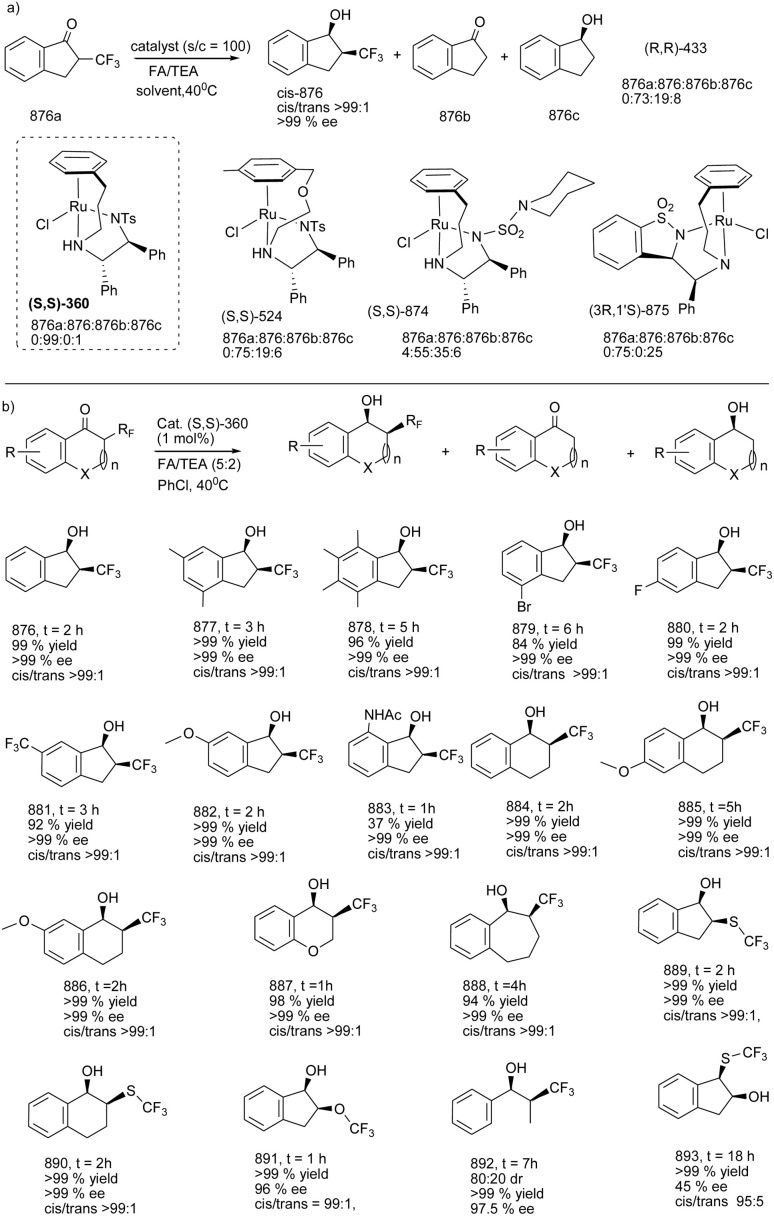
(a) Catalyst used to get optimized reaction condition. (b) Scope of the DKR–ATH. Reproduced from ref. 125 with permission from the American Chemical Society. Copyright 2022.

A. E. Cotman *et al.*^125^ (2022) reported an efficient dynamic kinetic resolution-asymmetric transfer hydrogenation (DKR–ATH) approach for the synthesis of β-CF_3_, β-SCF_3_, and β-OCF_3_ benzylic alcohols using 2-CF_3_-1-indanone as the model substrate. A series of five Noyori–Ikariya-type Ru(ii) catalysts were evaluated (Scheme 50a), including the parent catalyst 433, ether-tethered and oxy-tethered variants 360 and 524 (developed by Wills and Ikariya), as well as sulfamoyl-DPEN and benzosultam-based catalysts 874 and 875, respectively. All catalysts delivered excellent performance, providing the desired product 876 in high yield with outstanding stereocontrol, achieving *cis*/*trans* ratios greater than 99 : 1 and ee >99%. Although minor side products (876b and 876c) were observed, catalyst 360 proved to be the most efficient system, operating effectively at 1 mol% loading in the presence of a HCOOH/Et_3_N (5 : 2) hydrogen donor system in chlorobenzene at 40 °C, with reaction times ranging from 1 to 6 hours. Under optimized conditions, a wide range of indanone derivatives bearing substituents such as methyl, bromo, fluoro, trifluoromethyl, and methoxy groups were smoothly converted into the corresponding products (876–882) with consistently excellent stereoselectivity (*cis*/*trans* >99 : 1, >99% ee). However, substrates bearing an acetamido substituent showed reduced reactivity, affording only 37% yield (883). Tetralin-derived substrates also performed well (884–887), and benzosuberol (888) was obtained in excellent yield (94%). Importantly, fluorinated and trifluoromethoxy-substituted systems such as 2-SCF_3_ and 2-OCF_3_ derivatives were successfully synthesized with excellent yields (>99%, 889–891). The 2-trifluoromethoxy-1-indanol product (891) exhibited slightly reduced ee (96%). Compound 892 was also obtained efficiently under the standard conditions, whereas 1-SCF_3_-2-indanone (893) required an extended reaction time of 18 hours and showed lower ee (45%), indicating substrate-dependent limitations. The absolute configurations of selected products (876, 879, 881, and tetralin-derived 886) were confirmed by single-crystal X-ray diffraction (SCXRD). Interestingly, several of the obtained crystalline products (876, 879, 881, 886, 889, 890, and 891) exhibited elastically or plastically flexible behavior, suggesting potential applications in advanced functional materials. Overall, this study highlights the versatility of DKR–ATH methodology in accessing highly enantioenriched fluorinated and polyfluorinated alcohols with both synthetic and materials relevance.

**Scheme 51:**
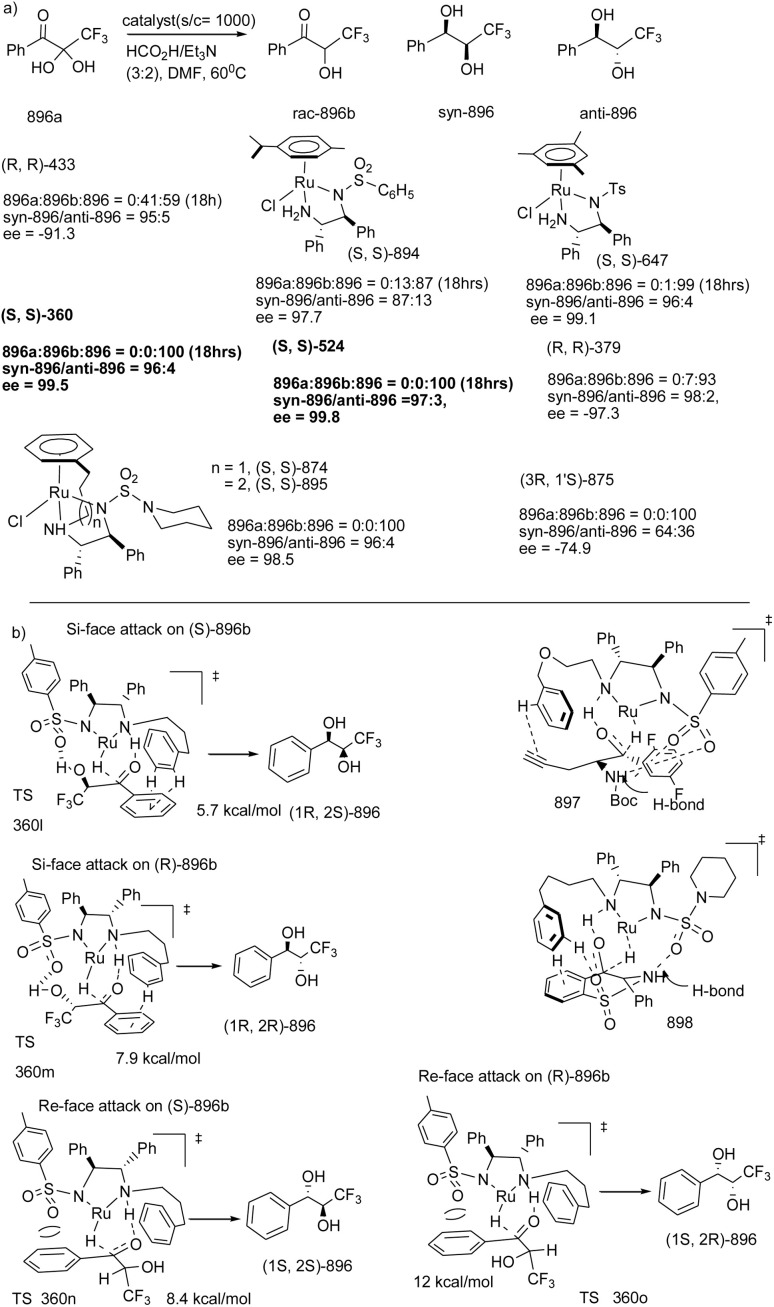
(a) Catalyst used in this reaction. (b) Optimized transition state (TS) geometries leading to the four stereomeric products. Reproduced from ref. 126 with permission from the American Chemical Society. Copyright 2023.

Cotman demonstrated^126^ (2023) that CF_3_-substituted syn-1,2-diols were synthesised from racemic α-hydroxyketones in the presence of formic acid and triethylamine through a process known as DKR. 1-Phenyl-2-trifluoromethylethanedione was reduced with the ATH process; several catalysts were tested to achieve optimal results (Scheme 51a). Catalysts 360, 524, 874, and 895 achieved complete conversion of the product, but the highest stereoselectivity was observed for catalysts 360 (99.5% ee) and 524 (99.8% ee), so further studies were conducted with these two catalysts. Density Functional Theory (DFT) calculations were performed to investigate the origin of the selectivity of product 896, in the presence of catalyst, the 896a substrate produced different enantiomeric products, (1*R*,2*S*)-896, (1*R*,2*R*)-896, (1*S*,2*S*)-896, and (1*S*,2*R*)-896 (Scheme 51b). The individual prereaction complexes (PrC) involving substrate rac-896b and the catalyst at an infinite distance were optimized, and their energies were calculated. The relative stability of individual reactants was found to be −7.0, −9.2, −5.2, and −0.9 kcal mol^−1^, respectively. This stability energy explains why (1*R*,2*S*)-896 and (1*R*,2*R*)-896 were produced more readily than the other enantiomers. The activation energies for the formation of the enantiomers were determined as follows: (1*R*,2*S*)-896 (12.7 kcal mol^−1^), (1*R*,2*R*)-896 (17.1 kcal mol^−1^), (1*S*,2*S*)-896 (13.6 kcal mol^−1^), and (1*S*,2*R*)-896 (12.9 kcal mol^−1^) with respect to each PrC. The results indicate that (1*R*,2*S*)-896 was the most favorable product compared to the other enantiomers. The favored transition state (TS-360l, Scheme 51b) leading to the formation of (1*R*,2*S*)-896 is stabilized by a hydrogen bond between the SO_2_ group of the catalyst (1.92 Å) and the ketone of the substrate, alongside a CH/π interaction between the arene and the ketone. In the alternative disfavored transition state (TS-360m) for the si-face attack on (*R*)-896b, the hydrogen bond distance was 1.75 Å, with a weak CH/π interaction due to the orientation of the aromatic ring. In the case of the re-face attack on both enantiomers, there were no hydrogen bond interactions, and a repulsive force between the SO and π systems disfavored product formation (TS-360n, TS-360o).

**Scheme 52:**
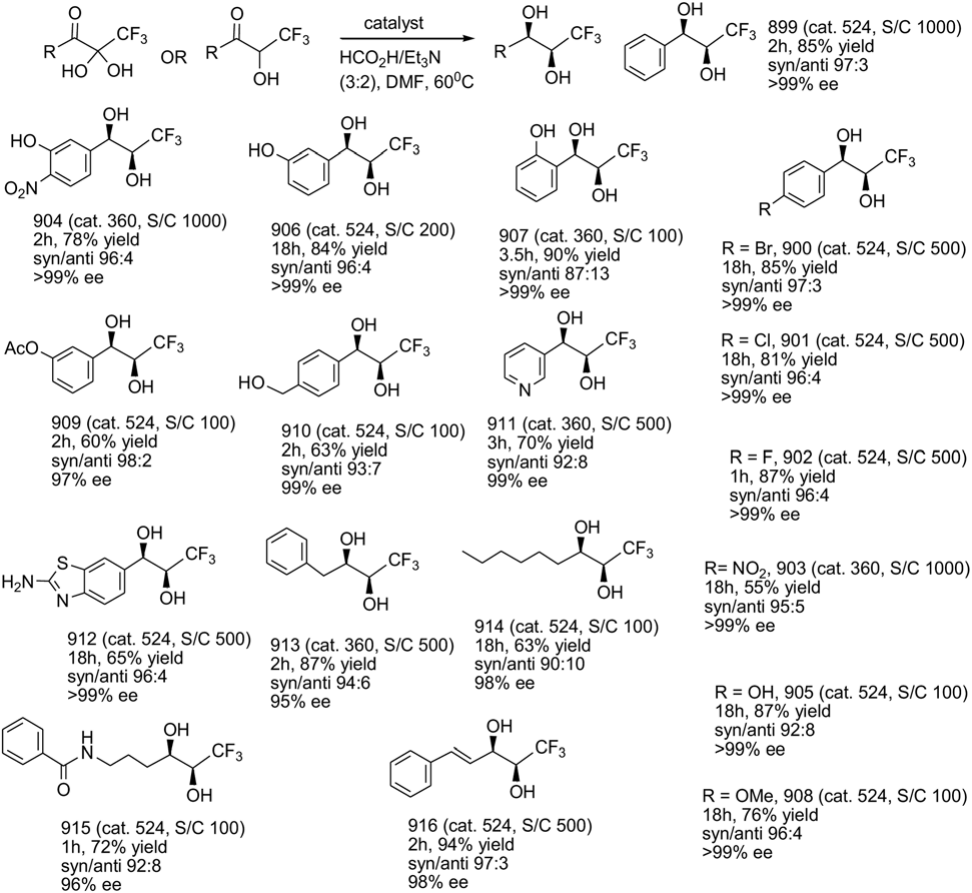
Scope of the CF_3_-subsituted 1,2-diols for Ru(ii)-catalyzed ATH-DKR. Reproduced from ref. 126 with permission from the American Chemical Society. Copyright 2023.

The ATH-DKR process has been documented in the literature; there were multiple non covalent interaction, including SO⋯NH hydrogen bonding, CH/π interactions, and η^6^-arene–substrate interactions (897 (ref. 127), 898 (ref. 128)), which played crucial role in controlling the stereochemical outcome. The starting material, CF_3_-substituted 1, 2-diketones, was prepared for reduction, and racemic monoalcohols were also synthesised for the reduction process. The two best-performing catalysts, 360 and 524, were employed for the ATH–DKR process, yielding excellent ee and dr for aryl ketones, particularly those with electron-withdrawing (902–904) and electron-donating groups (Scheme 52), although a higher amount of catalyst was necessary for electron rich system (905–909). Notably, aldehyde-containing 1,2-diketones were reduced simultaneously both functional group with high ee (910, 99%). Heterocyclic substrates were reduced with excellent ee (911, 912, 99%), whereas benzyl ketone was reduced using catalyst 360, producing an alcohol product with slightly lower ee (95%, 913). Alkyl and amide containing substrates were tolerated in this reducing process, achieving ee ranging from 96% to 98% (914, 915). Even, the α, β-unsaturated ketone was smoothly reduced to yield an alcohol product (916) with outstanding ee.

**Scheme 53:**
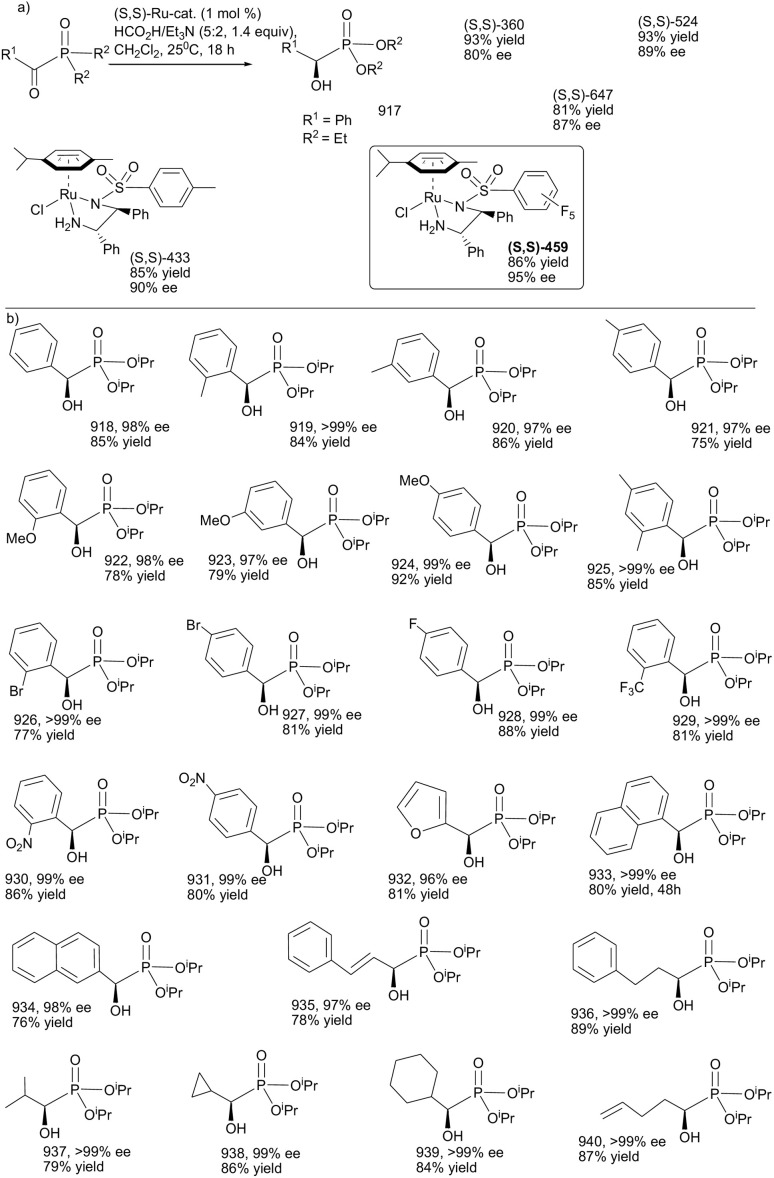
Catalyst and substrate scope. Reproduced from ref. 129 with permission from the American Chemical Society. Copyright 2023.

D. Virieux et *al*^129^ (year 2023) demonstrated the efficiency of ATH of α-ketophosphonates, leading to the production of enantiomerically enriched α-hydroxy phosphonates using Ru catalysts. This study addresses the previously unexplored phosphorus-containing ketone substrate in the ATH reaction. The reaction was performed using an α-substituted ketophosphonate substrate with the Noyori–Ikariya Ru(ii)–TsDPEN catalyst (*S*,*S*)-433, a 5 : 2 mixture of formic acid and triethylamine, was employed at a temperature of 25 °C for 18 hours (Scheme 53). To assess the selectivity of the reaction, both non-polar solvents (THF and Me–THF) and polar solvents (CH_3_CN, EtOAc, and dichloromethane) were utilised. It was observed that the yields were approximately the same across all solvents; however, the use of dichloromethane resulted in a higher ee in the product. A series of catalysts was then evaluated, including (*S*,*S*)-647, (*S*,*S*)-360, (*S*,*S*)-524, (*S*,*S*)-459. Catalyst 647, bearing a mesitylene ligand, was more sterically demanding than the *p*-cymene-based catalyst 433, yet it delivered lower stereoselectivity. Tethered Ru(ii) catalysts (360 and 524), featuring a covalent linkage between the diamine and the arene unit, showed good catalytic performance. Among all systems, catalyst 459, which contains an electron-deficient pentafluoro benzene sulfonyl group on the chiral diamine ligand, exhibited the highest ee. In addition, increasing steric bulk at the phosphorus substituent of the substrate further enhanced stereo control, with the isopropyl group giving the best results. Under optimized conditions using catalyst 459 in dichloromethane, a wide range of α-substituted keto phosphonates was successfully reduced. Electron-donating substituents such as methyl and methoxy groups afforded α-hydroxy phosphonates (919–925) in good to excellent yields (75–92%) with very high ee (97% to >99%). Electron- withdrawing groups, including bromo, fluoro, trifluoromethyl, and nitro substituents, also performed well, giving products (926–931) with consistently excellent ee (up to 99%). Heteroaryl and unsaturated substrates were also well tolerated. The 2-furyl and alkenyl derivatives provided products 932 and 935 in 81% and 78% yield, respectively, with high enantioselectivities (96–97% ee). Naphthyl-substituted substrates showed excellent reactivity, affording products (933–934) in high yield with >99% and 98% ee. A broad range of alkyl-substituted α-keto phosphonates, including linear, branched, and cyclic groups, also underwent smooth reduction, delivering products (935–940) in 79–89% yield with up to 99% ee. Finally, the methodology was successfully demonstrated on a gram scale, providing compound 940 in 87% isolated yield with 99% ee. This compound is particularly valuable as a key intermediate in the synthesis of nucleotide phosphonate analogues, highlighting the practical significance of this ATH protocol.

**Scheme 54:**
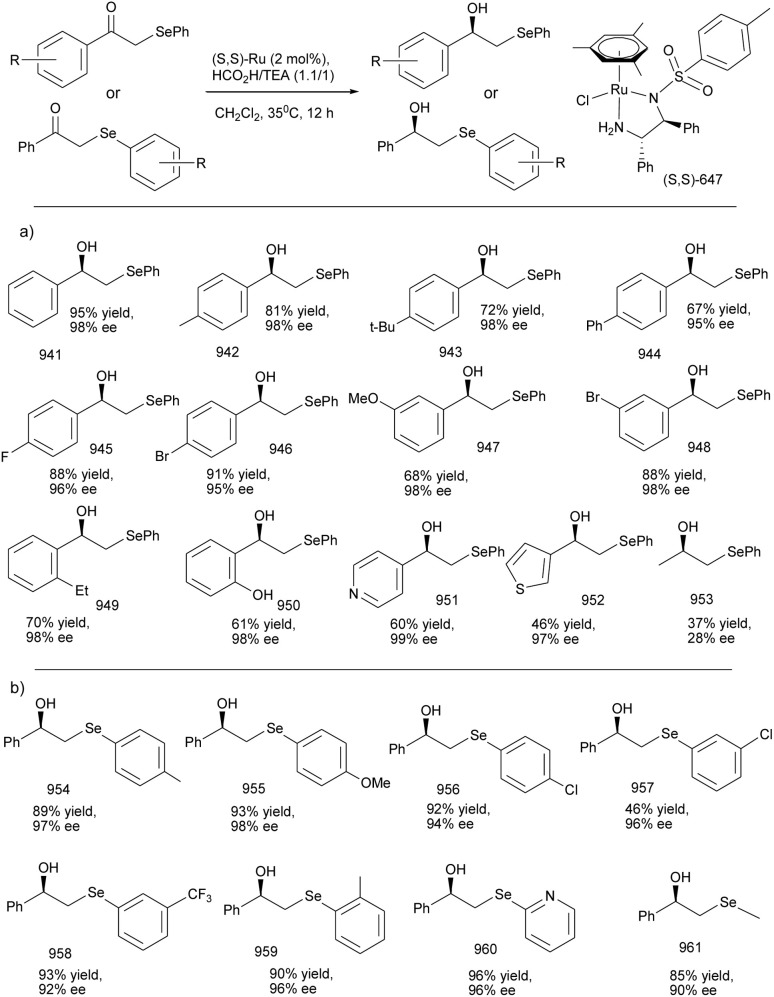
Substrate scope of synthesis of chiral β-hydroxy selenides from α-aryl selenomethyl ketones. Reproduced from ref. 130 with permission from Wiley-VCH. Copyright 2023.

The Zhou group^130^ (year 2023) reported the use of ruthenium catalysis to synthesis of chiral β-hydroxy selenides from α-aryl selenomethyl ketones with good yield and enantioselectivity (90–99% ee, Scheme 54). The substrate 1-phenyl-2-(phenyl selenyl) ethenone was reduced in the presence of chiral diamine ruthenium (2 mol%), formic acid/triethyl amine in a dicloromethane solvent at 35 °C for twelve hours. Substituents have some influence on the streoselectivity outcome; electron-donating substituents, such as –Me and –^*t*^Bu, yielded alcohol with an ee of 98% (942, 943), whereas a simple phenyl substituent at the *para* position led to slightly reduced ee (944, 95%). Additionally, halogen-substituted derivatives, including *para*-fluoro and *para*-bromo groups, also performed well, delivering products 945 and 946 with 95–96% ee. *Meta*-substituted derivatives bearing methoxy and bromine groups afforded products 947 and 948 with 98% ee, while *ortho*-substituted ethyl and hydroxyl groups also maintained high ee (949 and 950, 98%). Heteroaryl substrates such as thienyl and pyridyl ketones were smoothly reduced to the corresponding β-hydroxy selenides (951 and 952) with excellent stereocontrol. However, aliphatic α-phenylselenyl ketones showed significantly reduced reactivity and enantioselectivity, giving only 37% yield and 28% ee (953). This variation in enantioselectivity is attributed to differential edge-to-face π-interactions between the η^6^-arene ligand of the ruthenium catalyst and the aromatic ring of the substrate, which modulate transition-state stability. The methodology was also extended to α-arylselenyl acetophenones (Scheme 54b). Substrates bearing electron-withdrawing groups such as chlorine, as well as electron-donating groups such as methoxy and methyl, were smoothly converted into the corresponding alcohols with excellent ee (954–956, 94–98%). *Meta*-substituted chloro and trifluoromethyl groups, as well as *ortho*-methyl substituents, also provided products 957–959 with good to very good ee (92–96%). Pyridyl-substituted ketones were successfully reduced to chiral β-hydroxy selenides (960), and alkyl-substituted selenyl ketones also underwent efficient reduction to afford the desired alcohol product (961). Importantly, this catalytic system was successfully applied to gram-scale synthesis and further derivatization of chiral β-hydroxy selenides, demonstrating its practicality and synthetic utility in asymmetric catalysis.

**Scheme 55:**
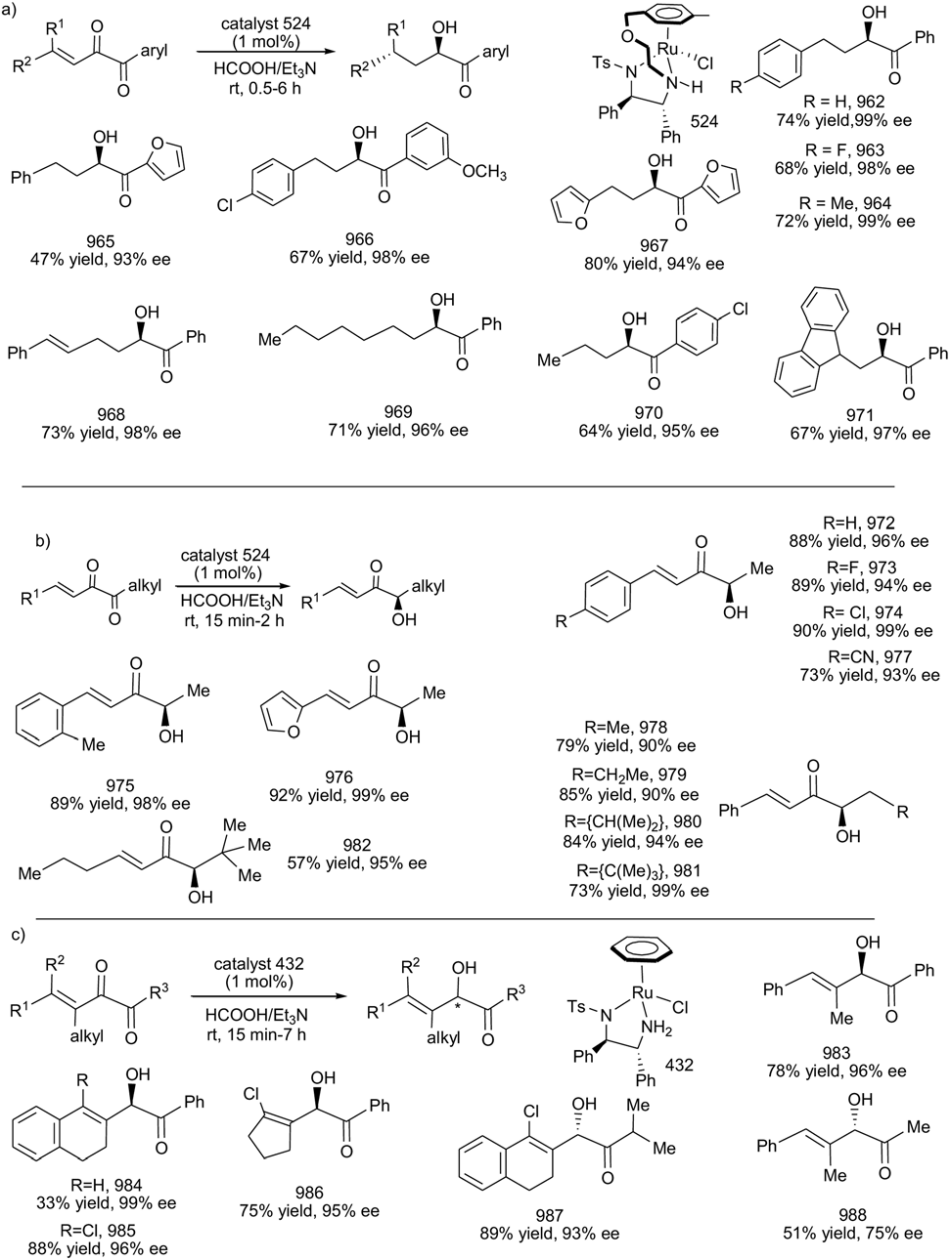
Scope of substrates with (a) Aryl ketone moiety. (b) Conjugated α-diketones with alkyl ketone units and (c) β-Alkyl substituents. Reproduced from ref. 131 with permission from the American Chemical Society. Copyright 2024.

**Scheme 56:**
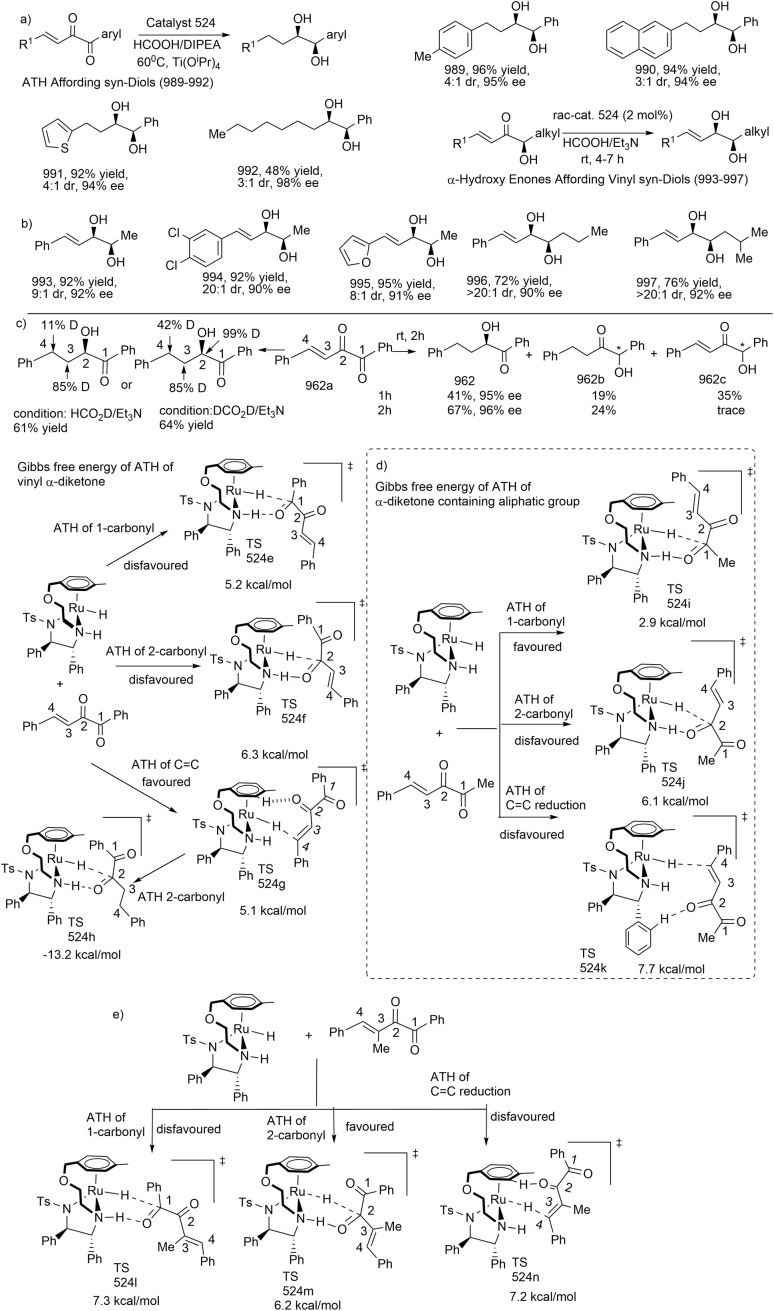
(a) ATH affording syn-diols. (b) α-Hydroxy enones affording vinyl syn-diols. (c, d and e) D-labeling experiment and Gibbs free energy of ATH. Reproduced from ref. 131 with permission from the American Chemical Society. Copyright 2024.

Fang *et al.*^131^ (2024) demonstrated that β, γ-unsaturated α-diketone substrates can be reduced with excellent regioselectivity and enantioselectivity using a ruthenium-based ATH system. In the case of vinyl α-diketones, the reaction proceeded smoothly to afford α-hydroxy enones with high control over both regio- and stereoselectivity. Substrates bearing electron-donating or electron-withdrawing groups on the aryl ring (*R*^2^) delivered the corresponding alcohols in 68–72% yields with excellent enantioselectivities (Scheme 55, 98–99% ee, compounds 963–964). A broad range of functional groups was well tolerated. Aryl- and furan-containing ketones also underwent efficient reduction to give the desired products with excellent enantioselectivity (965). When both *R*^1^ and *R*^2^ substituents were varied simultaneously, the reaction still proceeded efficiently, affording products in very good yields with outstanding enantiocontrol (966–967). In addition, styryl- and alkyl-substituted ketones were successfully reduced, providing products with high ee (95–98%, 968–969). Even sterically demanding γ, γ-disubstituted diketones underwent smooth reduction, delivering product 971 with very good ee. Variation of the *R*^1^ substituent further highlighted the robustness of the system. Substrates bearing methyl groups, substituted phenyl rings (electron-donating or electron-withdrawing), and furyl groups afforded alcohols in 73–92% yields with excellent enantioselectivities (93–99% ee, 972–977). Alkyl-substituted substrates such as ethyl, propyl, isopropyl, and tributyl groups were also well tolerated, giving products with 90–99% ee (978–981). Although substrates containing two aliphatic substituents showed slightly reduced yields (982, 57%), the ee remained high (95%). Importantly, conjugated α-diketones bearing β-alkyl substituents were efficiently converted into α-hydroxy-β, γ-unsaturated ketones with good yields and excellent ee. Substituted phenyl propenyl and benzocyclohexenyl alkenes also participated effectively in the ATH process, affording products 983 and 984 with 96–99% ee. Cyclic alkenes bearing chloro substituents were likewise compatible, giving products 985 and 986 in 75–88% yield with 95–96% ee. A range of alkyl-substituted substrates (987–988) was also successfully reduced, demonstrating the broad applicability of the method. Overall, this study provides an efficient strategy for the selective mono-reduction of β, γ-unsaturated α-diketones, enabling access to valuable chiral building blocks. Notably, under modified conditions with Ti(OiPr)_4_, syn-diol products could be obtained, further expanding the synthetic utility of this system. Good catalytic performance was observed for aryl-, naphthyl-, and thienyl-substituted substrates, providing diols (989–992, Scheme 56a) in good yields with high stereocontrol. Using racemic catalyst 524, α-hydroxy enones were also converted into syn-diols (993–997) with 72–95% yields, 90–92% ee, and high dr. Mechanistic investigations supported a stepwise reduction pathway. Control experiments showed time-dependent formation and consumption of intermediates (962a–c), indicating that 962c acts as a key intermediate route to the final product. In experiments with HCO_2_D, deuterium incorporation at positions 4 and 3 was observed at 11% and 85% of 962, respectively. On the other hand, using DCO_2_D led to deuterium incorporation at positions 4, 3, and 2 of 42%, 85%, and 99%, respectively. All experiments indicated that the 1,4-reduction pathway was more favorable.

Subsequently, DFT calculations were performed to confirm the reaction mechanism. Three pathways were examined: the 1-carbonyl reduction pathway, the 2-carbonyl reduction pathway, and the 3, 4 C=C reduction followed by the 2-carbonyl reduction pathway (Scheme 56c). The activation barriers were found to be 5.2 (TS-524e), 6.3 (TS-524f), and 5.1 (TS-524g) kcal mol^−1^, respectively, indicating that both the 1-carbonyl reduction and the 3, 4 C=C reduction pathways were of similar energy. However, the product from the 3, 4 C=C reduction pathway was thermodynamically more stable. For substrate 962a, the first reduction occurred at the 3, 4 C=C position, followed by the reduction of the 2-carbonyl to yield the final product 962*via* TS-524h. For the substituted α-diketone substrate, activation energies were calculated as 2.9 kcal mol^−1^ for 1-carbonyl reduction (TS-524i), 6.1 kcal mol^−1^ for 2-carbonyl reduction (TS-524j), and 7.7 kcal mol^−1^ for C=C reduction (TS-524k) (Scheme 56d). The transition energy was 2.9 kcal mol^−1^, which was lower than that for substrate 962a; this difference was attributed to hydrogen bonding (H⋯O) between the α–H of the alkyl ketone (972) and the oxygen atom of the Ts group, which was absent for substrate 962a. The β-alkyl substituent was investigated for the hydride transfer process, with activation barriers of 7.3 kcal mol^−1^ for 1-carbonyl reduction (TS-524l), 6.2 kcal mol^−1^ for 2-carbonyl reduction (TS-524m), and 7.2 kcal mol^−1^ for C=C reduction (TS-524n) (Scheme 56e). For the 2-carbonyl reduction process, the methyl group does not affect any, but for C=C and 1-carbonyl reduction processes, the presence of the methyl group affects the distortion of the conjugation structure and introduces steric hindrance, which is *a* factor in the energetically unfavorable nature of these two processes. Substrate effects were rationalized: hydrogen bonding interactions and steric effects from β-substituents significantly influenced the relative activation barriers, thereby controlling selectivity in both carbonyl and olefin reduction steps.

**Scheme 57:**
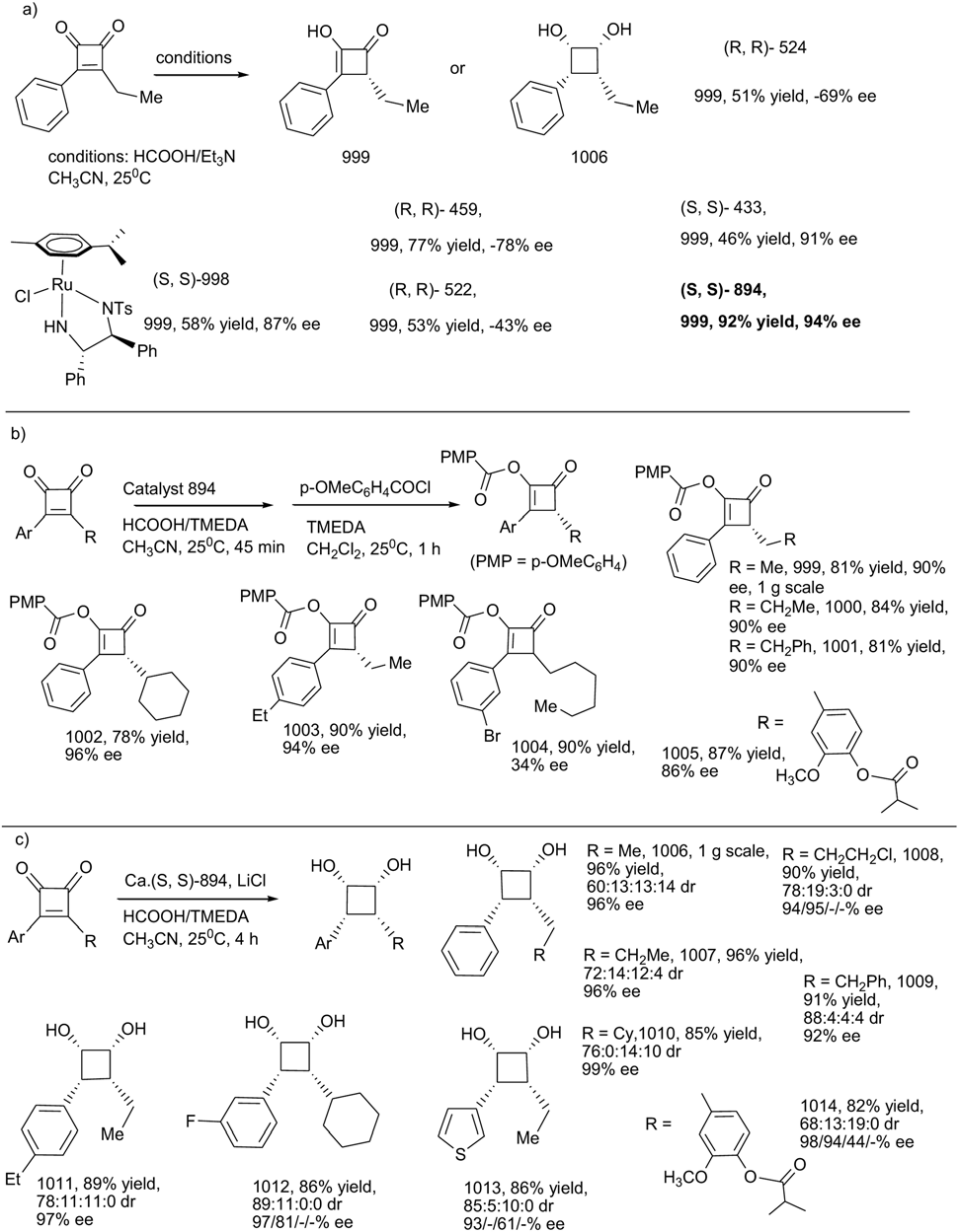
(a) Condition optimizations for the ATH of cyclobutenedione. (b) Substrate scope for cyclobutenone formation. (c) Substrate scope of cyclobutanediol synthesis. Reproduced from ref. 77 with permission from the American Chemical Society. Copyright 2024.

**Scheme 58:**
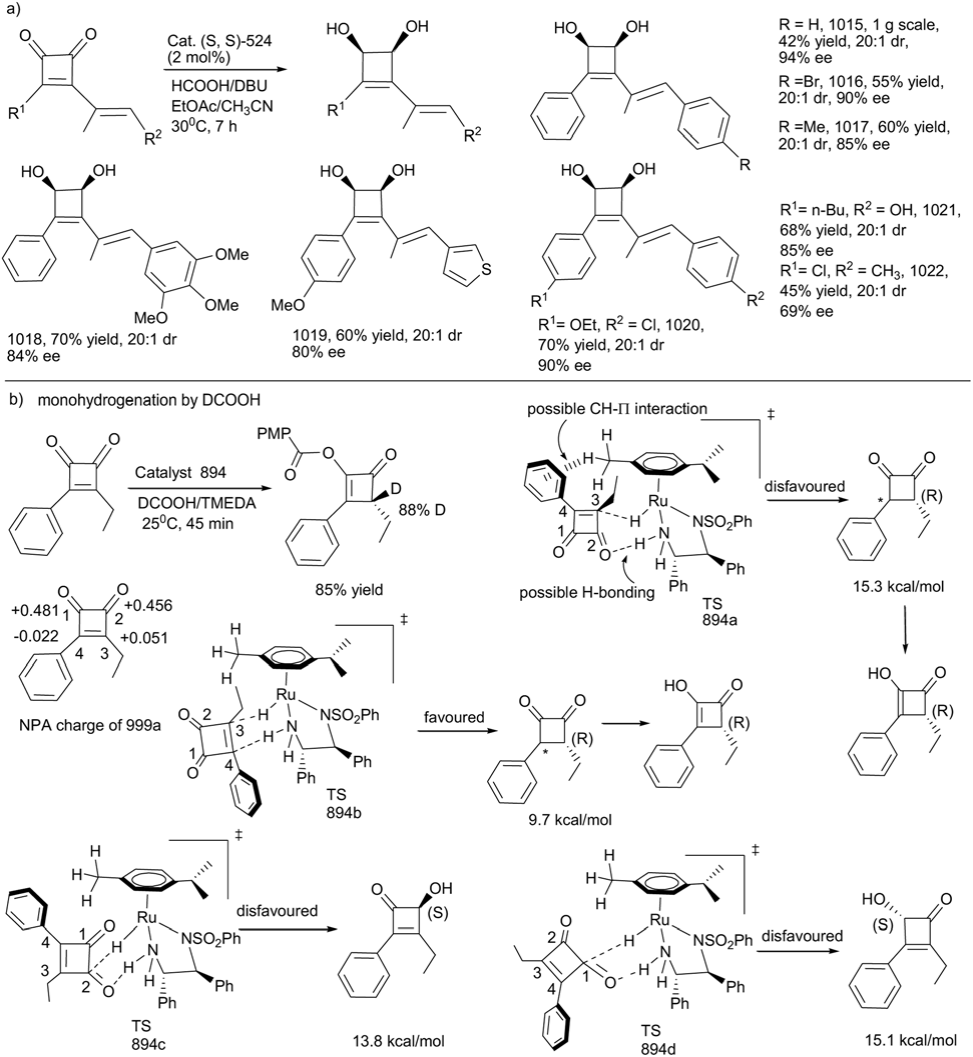
(a) Substrate scope of cyclobutenediol synthesis. (b) Proposed transition structures. Reproduced from ref. 77 with permission from the American Chemical Society. Copyright 2024.

**Scheme 59:**
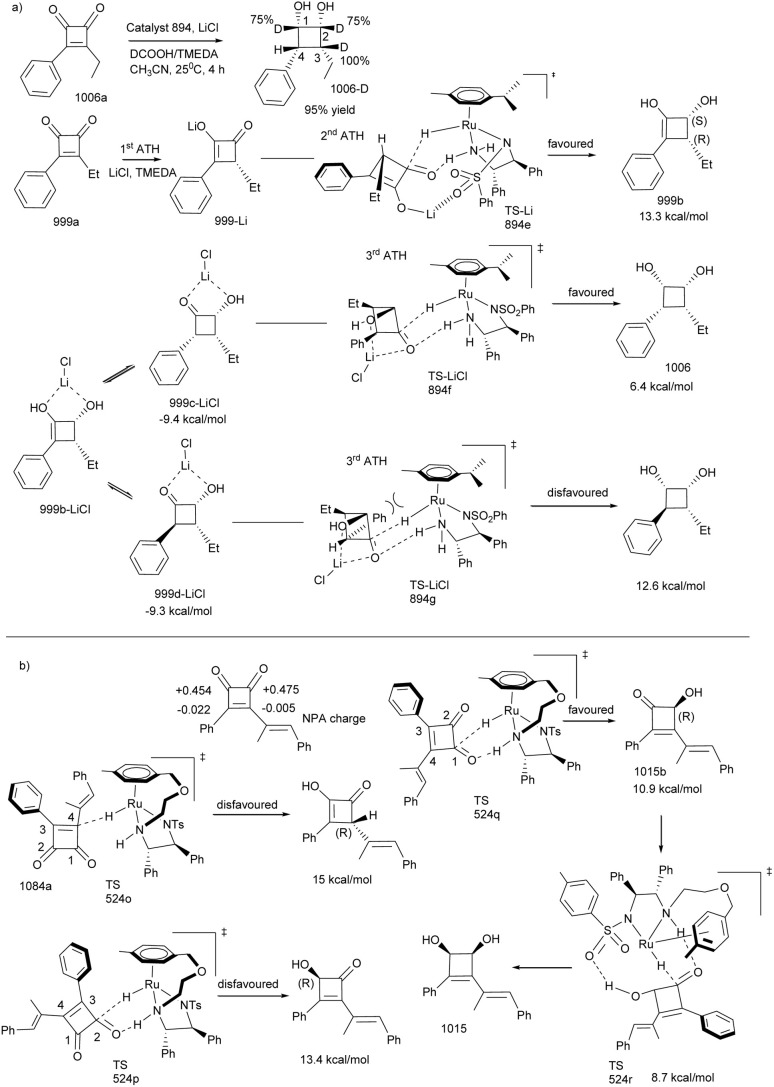
DFT calculations and postulated mechanism for the formation of products (a) cyclobutenediol (b) 3-phenyl-4-propenyl cyclobutenedione. Reproduced from ref. 77 with permission from the American Chemical Society. Copyright 2024.

Fang *et al*.^77^ (2024) investigated the ATH of cyclobutenediones using Noyori–Ikariya-type ruthenium catalysts, enabling the formation of three distinct types of highly enantioenriched four-membered ring products with excellent regio- and stereocontrol. A series of catalysts was screened, among which catalyst 894 exhibited the best overall performance. Under optimized conditions using phenyl-substituted cyclobutenediones, formic acid (HCO_2_H) as a hydrogen source, and TMEDA in acetonitrile, the reaction afforded the monohydrogenated alcohol product in 92% yield with 95% ee (999). In the presence of LiCl, the reaction pathway was altered to favor double reduction, delivering the corresponding dihydrogenated diol product in 99% yield and 97% ee. Substrate scope studies revealed that cyclobutenediones bearing alkyl substituents such as methyl, ethyl, and benzyl were smoothly converted to the corresponding monohydrogenated alcohols in good yields (81–84%) with high ee (90%, 999–1001). Due to the instability of some products, *p*-OMeC_6_H_4_CO protection was employed to stabilize the alcohol functionality (Scheme 57b). Cyclohexyl-substituted substrates also performed well, giving 78% yield with excellent ee (96%; 1002). The influence of electronic effects was evident: substrates bearing electron-donating and electron-withdrawing substituents on the phenyl ring generally afforded good yields and high ee (1003, 1004–1005), whereas the bromo-substituted substrate showed significantly reduced ee (34%; 1004), highlighting the sensitivity of the system to steric and electronic perturbations. Natural product-derived systems such as vanillin isobutyrate were also successfully reduced, giving 87% yield with 86% ee (1005). In the presence of LiCl, alkyl-substituted cyclobutenediones underwent sequential ATH to furnish diol products with excellent efficiency and stereocontrol (1006–1009). A cyclohexyl-substituted example delivered 85% yield with 99% ee (1010). Additional functionalized substrates bearing electron-withdrawing (Et) and electron-donating (F) groups on the phenyl ring also gave high yields and enantioselectivities (1011–1012). Heteroaryl systems such as thienyl derivatives were well tolerated, affording products in 86% yield with 93% ee (1013), while more complex substrates, including vanillin isobutyrate and L-menthol-derived systems, were also compatible (1014). However, a 3-phenyl-4-propenyl-substituted substrate showed poor reactivity with catalyst 894, and only upon switching to catalyst 524 was the expected product obtained in 42% yield with excellent regioselectivity (dr 20 : 1) and 94% ee (1015). Mechanistic investigations supported a stepwise, metal–ligand cooperative pathway (Scheme 58b). DFT calculations and NPA analysis revealed polarization of the C=C bond in substrate 999a, with C4 bearing a partial negative charge and C3 bearing a partial positive charge. Deuterated experiments indicated that deuterium (D) from DCO_2_H was incorporated into the C3 position in 999a diketone. This suggests that a proton from the amine group (NH) transfers to the double bond, while a hydride from the metal migrates to the ethyl carbon (C3). The transition state (TS-894a) for the conjugated reduction was calculated, showing that it had a slightly weak C–H⋯π interaction between the phenyl ring of the substrate and the methyl hydrogen of the catalyst, with an activation barrier of 15.3 kcal mol^−1^. When the ethyl group of the substrate and the phenyl ring of the catalyst were on the same side, giving a more favorable orientation; the activation barrier (TS-894b) decreased to 9.7 kcal mol^−1^. For the 2-carbonyl reduction and 1-carbonyl reduction, the activation energies were calculated as 13.8 kcal mol^−1^ (TS-894c) and 15.1 kcal mol^−1^ (TS-894d), respectively, which are unfavorable pathway. To produce the cyclobutenediol product, mechanistic details were studied, and a deuterated experiment was conducted using 1006a and DCOOH (Scheme 59). The product 1006-D was obtained, with 75% D incorporation at the 1 and 2 positions and 100% D incorporation at the 3-position, while no D was incorporated at the 4-position. After the first alkyl ATH of cyclobutenedione, LiCl coordinates with the oxygen of the substrate to form the intermediate 999-Li. Subsequently, the second ATH occurs *via* TS-Li-894e, in which the Li^+^ ion was bonded to the enolate oxygen and the SO_2_ group of the catalyst, which had an activation barrier of 13.3 kcal mol^−1^, resulting in the final product 999b. Next, the intermediate 999b-LiCl was in equilibrium with keto-forms 999c-LiCl and 999d-LiCl, which had energy changes of −9.4 and −9.3 kcal mol^−1^, respectively. The final product (1006) was obtained through a more favourable transition state, TS-LiCl-894f, with an activation energy of 6.4 kcal mol^−1^, while the disfavored activation barrier was calculated (TS-LiCl-894g), which had an energy of 12.6 kcal mol^−1^. This disfavors due to steric hindrance between the lone pair of the SO_2_ group and the π-bond of benzene. To produce the diol compound from 3-phenyl-4-propenyl cyclobutenedione (1015), the first ATH occurred preferentially *via* 1-carbonyl reduction, which had an activation barrier of 10.9 kcal mol^−1^ (TS-524q), leading to the product *R*-1015b, followed by the second ATH through TS-524r, which had an activation energy of 8.7 kcal mol^−1^, leading to the final product 1015. This transition structure was stabilized by the hydrogen bonding between the hydroxyl group (OH) of the products and the –SO_2_ group of the catalyst, thereby reducing the activation barrier. Overall, this study highlights a mechanistic understanding of catalyst-controlled regioselectivity, DKR, and stepwise ATH of highly strained cyclobutenedione systems.

**Scheme 60:**
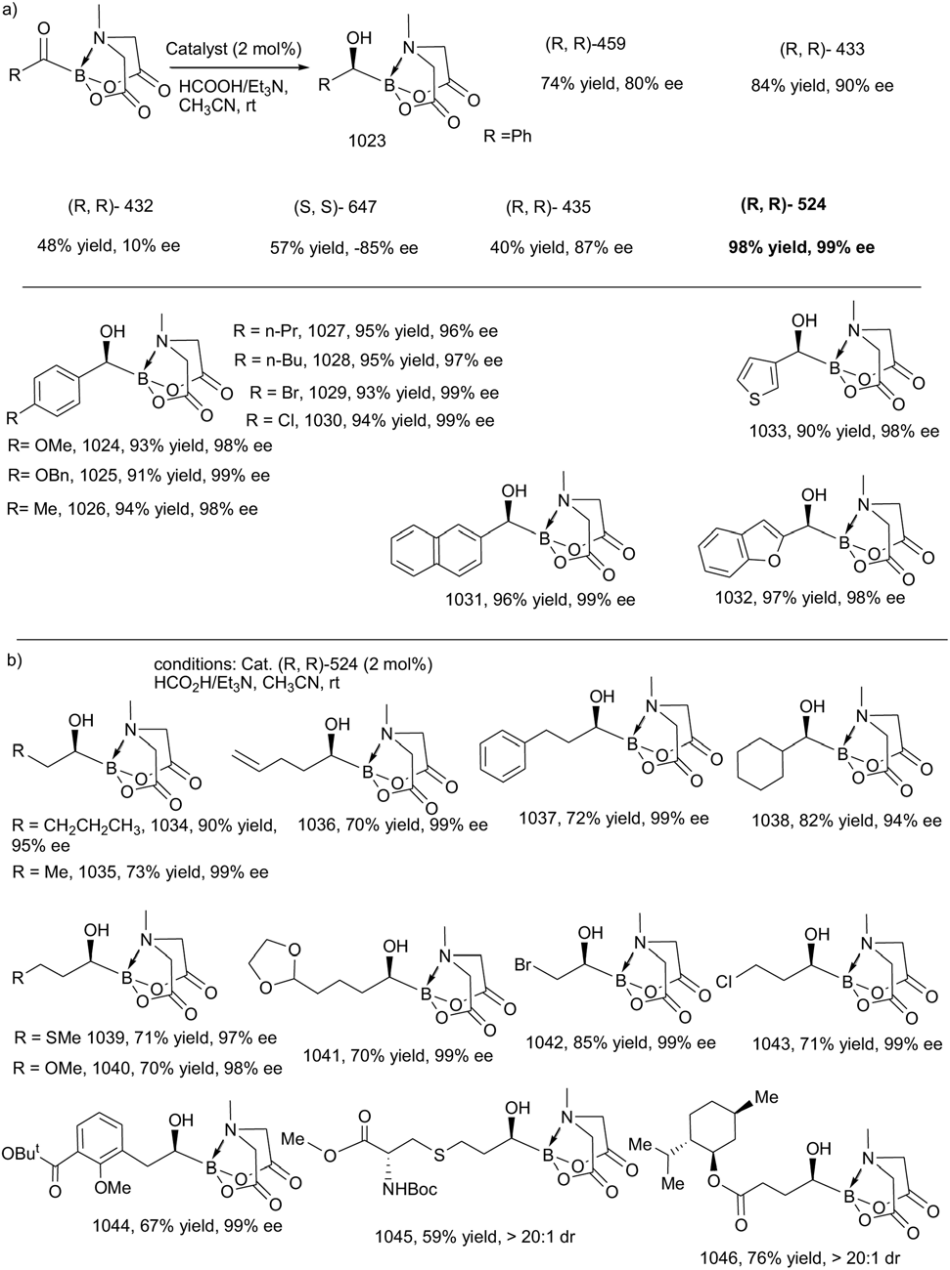
Substrate scope of (a) Aromatic acylboronates. (b) Aliphatic acylboronates. Reproduced from ref. 132 with permission from the American Chemical Society. Copyright 2024.

**Scheme 61:**
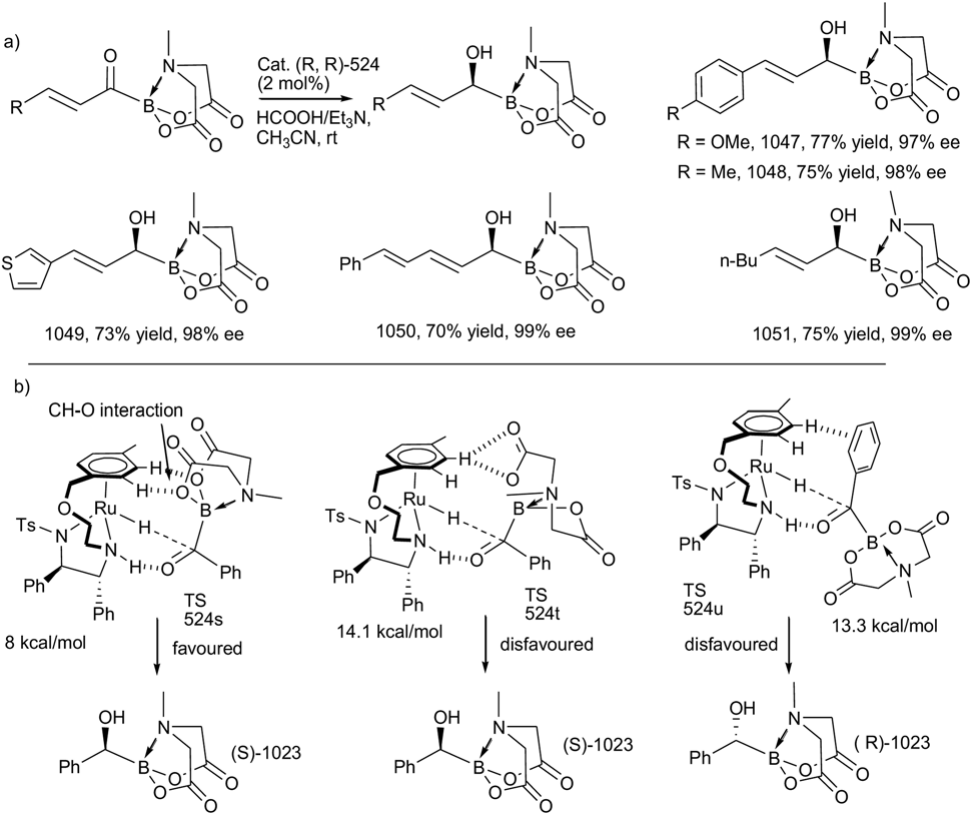
(a) Substrate scope of boryl enones. (b) Proposed transition state calculation. Reproduced from ref. 132 with permission from the American Chemical Society. Copyright 2024.

Fang *et al.*^132^ (year 2024) conducted a study on the ATH of *N*-methyliminodiacetyl (MIDA) acylboronates, the ability to produce highly enantioselective α-boryl alcohol products from various substituents, including (hetero)aryl, alkyl, alkynyl, alkenyl, and carbonyl groups (Scheme 60a). A series of catalysts was evaluated to optimize reaction conditions. Among them, catalyst (*R*,*R*)-524 demonstrating particularly promising results and was selected for further study. This catalyst reduced a series of acylboronates, resulting in the formation of boryl alcohol products with the (*S*) configuration, as confirmed by single-crystal X-ray crystallography. This *S*-configuration product indicated that servocontrol is governed by the inherent chiral environment of the catalyst rather than π–π interactions between the arene ring of the catalyst and the aryl ring of the substrate. This study is particularly useful for the ATH process because the acylboronates, a new functional group, are the primary factor responsible for the enantioselectivity of the product. The presence of electron-donating substituents on the phenyl ring, such as OMe, OBn, Me, *n*-Pr, and *n*-Bu, resulted in the formation of alcohol products with very good yields (1024–1028, 91–95%) and excellent ee (96–99%). Substrates with electron-withdrawing groups like Br and Cl provided corresponding alcohols with 99% ee (1029–1030). Functional groups such as naphthyl, furyl, and thiophene were tolerated, delivering the products with very good yields and outstanding ee (1031–1033, 98–99%). Various alkyl functional groups were employed in this reaction, leading to excellent ee (up to 99%). Substituents such as Bu, Et, butenyl, phenylethyl, and cyclohexyl smoothly reduced with good to very good yields (70–90%) and outstanding ee (1034–1038, 94–99%, Scheme 60b). Alkyl substrates containing SMe, OMe, acetal, Br, and Cl were compatible under the reaction conditions, providing good to very good yields and excellent ee (1039–1043). Amino esters and menthol esters were tolerated in this reaction, yielding products with an outstanding >20 : 1 dr (1044–1046). Interestingly, vinyl substrates with 4-OMeC_6_H_4_, 4-MeC_6_H_4_, thienyl, styryl, and *n*-Bu functional groups (Scheme 61a) were likewise reduced efficiently, giving the products with good yields and outstanding ee (1047–1049). To elucidate the mechanism of the ATH reaction, density functional theory (DFT) calculations were performed, leading to the identification of three possible transition states (Scheme 61b). In the most favourable pathway, the BMIDA group was oriented on the same side as the η^6^-arene of the catalyst, with interactions occurring between the two C–H bonds of the arene and the oxygen of MIDA (TS-524s); the activation barrier was only 8.0 kcal mol^−1^. In the second transition state (TS-524t), there was an interaction between one C–H bond of the arene and the oxygen of MIDA, with an activation barrier of 14.1 kcal mol^−1^. When the phenyl ring of the substrate and the arene ring of the catalyst were on the same side, there was only a C–H/π interaction with an activation barrier of 13.3 kcal mol^−1^ (TS-524u), which indicated that TS-524s was a more favorable path.

**Scheme 62:**
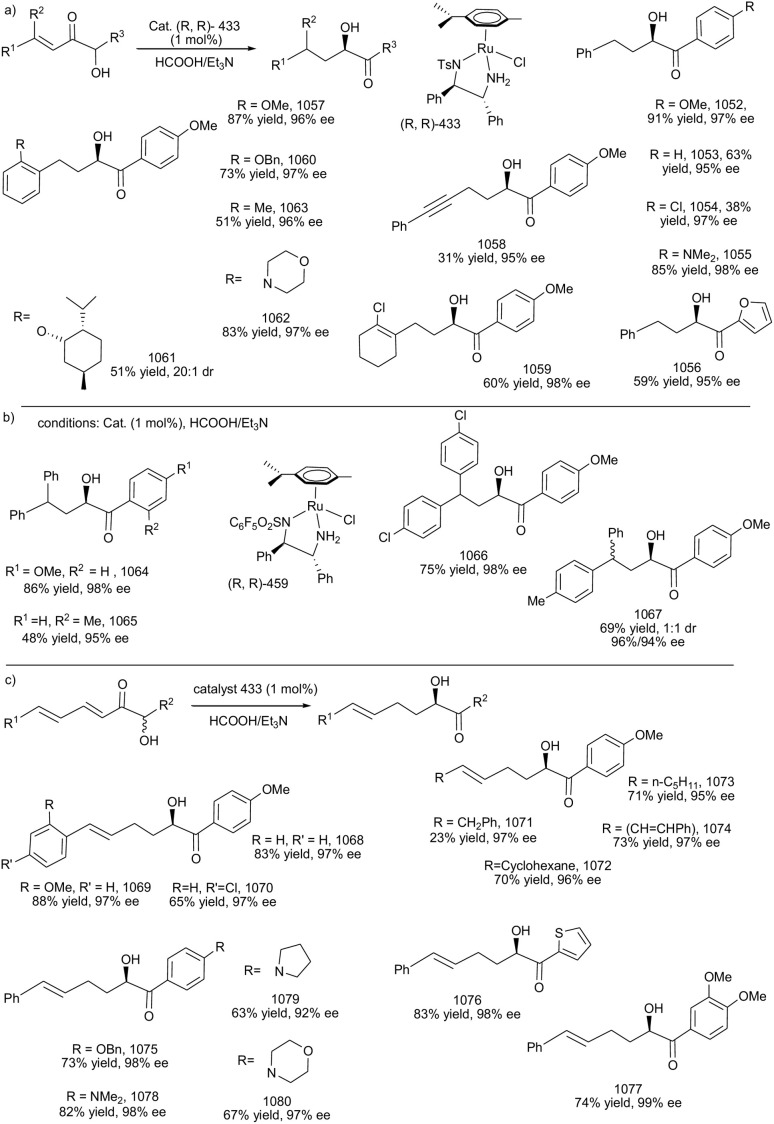
Scope of reductive isomerization of α-hydroxyenones and α-hydroxy vinyl enones. Reproduced from ref. 133 with permission from the American Chemical Society. Copyright 2024.

Fang *et al.*^133^ (2024) reported the asymmetric reductive isomerization of α-hydroxyenones *via* an ATH process using a ruthenium-based catalyst (Scheme 62). The study highlights a strong dependence of reactivity and enantioselectivity on the electronic nature of the substituents on the phenyl ring (*R*^3^). When *R*^3^ bears an electron-donating methoxy group (–OMe), the reaction proceeds efficiently, affording the product in 91% yield with excellent ee (97%; 1052). In contrast, an unsubstituted phenyl group results in a reduced yield of 63%, although the ee remains high (95%; 1053). The presence of an electron-withdrawing chloro substituent (-Cl) further decreases the yield to 38%, while still maintaining excellent ee (97%; 1054). Additionally, substrates bearing strongly electron-donating groups, such as 4-NMe_2_C_6_H_4_, also perform well, delivering products with excellent ee (98%; 1055). Heteroaryl systems, including furyl-substituted substrates, are well tolerated, providing products with high ee (95%; 1056). Substituents at the *R*^1^ position also influence reaction outcomes; for example, aryl rings bearing methoxy groups give good yields and excellent stereocontrol (1057). However, phenylethynyl-containing substrates show significantly reduced reactivity, affording only 31% yield, albeit with high ee (95%; 1058). These results clearly emphasize the crucial role of electronic effects in governing both efficiency and stereochemical outcome in ATH-based reductive isomerization. Further substrate scope evaluation revealed that chlorocyclohexenyl-containing systems provide moderate yields while maintaining excellent enantioselectivity (1059). Variations in both *R*^1^ and *R*^2^ substituents generally afforded products in moderate to very good yields with consistently high enantioselectivity (1060–1063). Using catalyst 459, a range of trisubstituted enones were efficiently reduced to the corresponding products with excellent ee (94–98%; 1064–1067) (Scheme 62b).

**Scheme 63:**
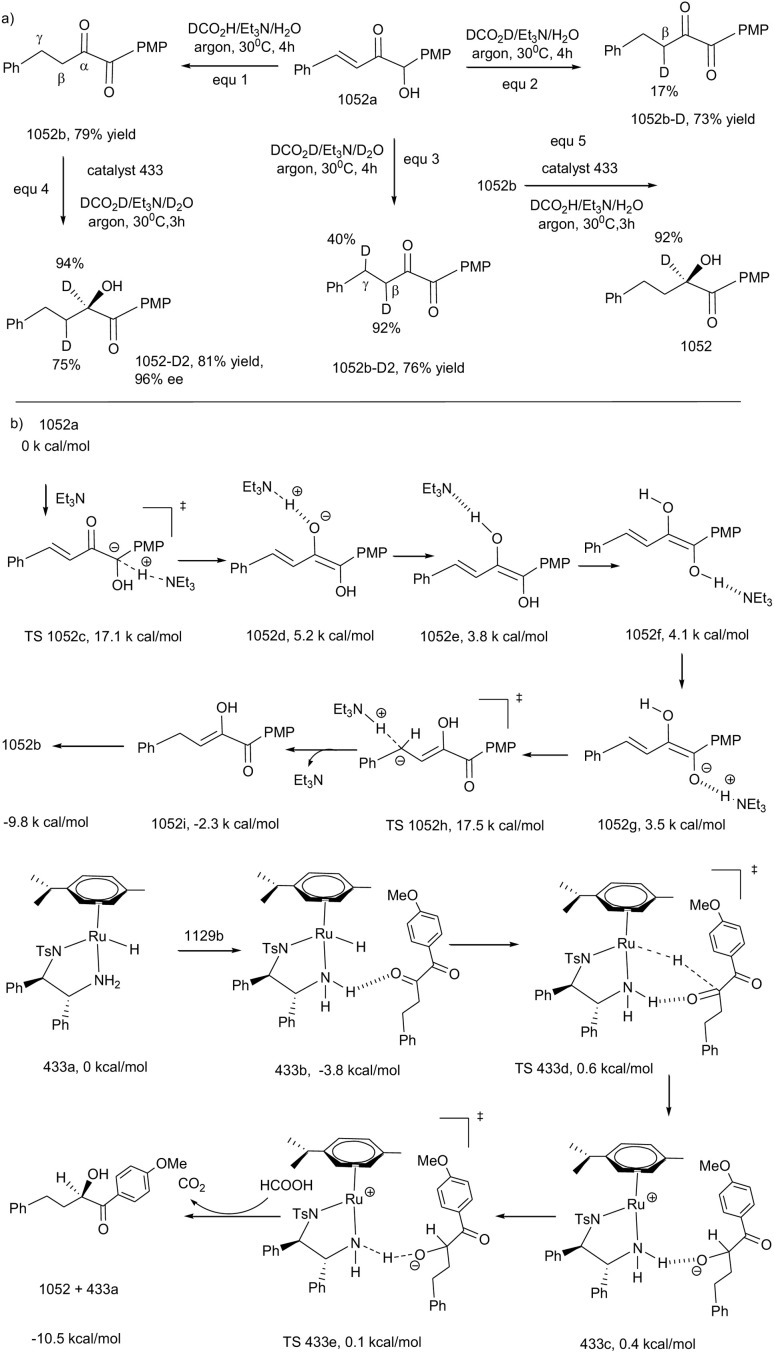
(a) D-labeling experiments and (b) DFT calculations on reductive isomerization of α-hydroxyenones. Reproduced from ref. 133 with permission from the American Chemical Society. Copyright 2024.

Conjugated dienes were also compatible under these conditions, delivering ATH products in good yield (83%) and excellent ee (97%), while selectively retaining one double bond (1068–1080) (Scheme 62c). Substrates bearing *ortho*-methoxy and *para*-chloro substituents also afforded high ee (1069, 1070). Benzyl-substituted systems showed lower yields but maintained excellent ee (1071, 97%), whereas cyclohexyl and pentyl substituents gave products in good yields with high stereocontrol (1072, 96% ee; 1073, 95% ee). Other functionalized systems, including phenyl-diene substrates and *R*^3^ groups such as 4-OBnC_6_H_4_ and thienyl moieties, were also successfully converted with good yields and excellent ee (1074–1076). Highly substituted aryl systems, including 3-OBn-4-OMeC_6_H_3_, 3,4-(OMe)_2_C_6_H_3_, and tertiary amine-containing substrates, were also well tolerated, consistently delivering products with outstanding ee (1077–1080). Mechanistic investigations were performed through a series of deuterium-labelling experiments (Scheme 63a). Treatment of 1052a with DCO_2_H/Et_3_N/H_2_O afforded diketone 1052b in 79% yield without deuterium incorporation (Scheme 63a, eqn (1)). In contrast, reaction with DCO_2_D led to 17% deuterium incorporation at the *β*-position (Scheme 63a, eqn (2)), while the combination of DCO_2_D and D_2_O resulted in incorporation at both *β*- and *γ*-positions (Scheme 63a, eqn (3)). Further experiments showed that 1052b treated with DCO_2_D/D_2_O gave 1052-D_2_ with 94% and 75% deuterium incorporation at the *α*- and *β*-positions, respectively (Scheme 63a, eqn (4)), whereas DCO_2_H/H_2_O led to selective α-deuteration (92%; Scheme 63a, eqn (5)). These results confirm a dynamic proton/deuteron exchange process during the reaction pathway. Density functional theory calculations were used to further insight into the mechanism of the reaction (Scheme 63b). The reaction starts with base-promoted deprotonation of 1052a by Et_3_N base, going through a transition state with an activation barrier of 17.1 kcal mol^−1^ (TS-1052c). Subsequent protonation steps mediated by Et_3_NH^+^ and intramolecular hydrogen bonding produce the intermediate 1052e, followed by deprotonation and re-protonation sequences that lead to form 1052i through TS-1052h (activation barrier 17.5 kcal mol^−1^). Then isomerization of this intermediate produces 1052b intermediate (Δ*G* = −9.8 kcal mol^−1^). The ruthenium catalyst helps hydrogen bonding between the ligand N–H and carbonyl oxygen, facilitating rapid proton transfer steps through low-energy transition states (TS-433d and TS-433e, barriers of 0.6 and 0.1 kcal mol^−1^, respectively), ultimately generating the final product 1052. In conclusion, this study showed a detailed mechanistic and substrate-dependent understanding of ruthenium-catalyzed ATH-driven reductive isomerization, illustrating how subtle electronic and hydrogen-bonding interactions control both reactivity and enantioselectivity.

**Scheme 64:**
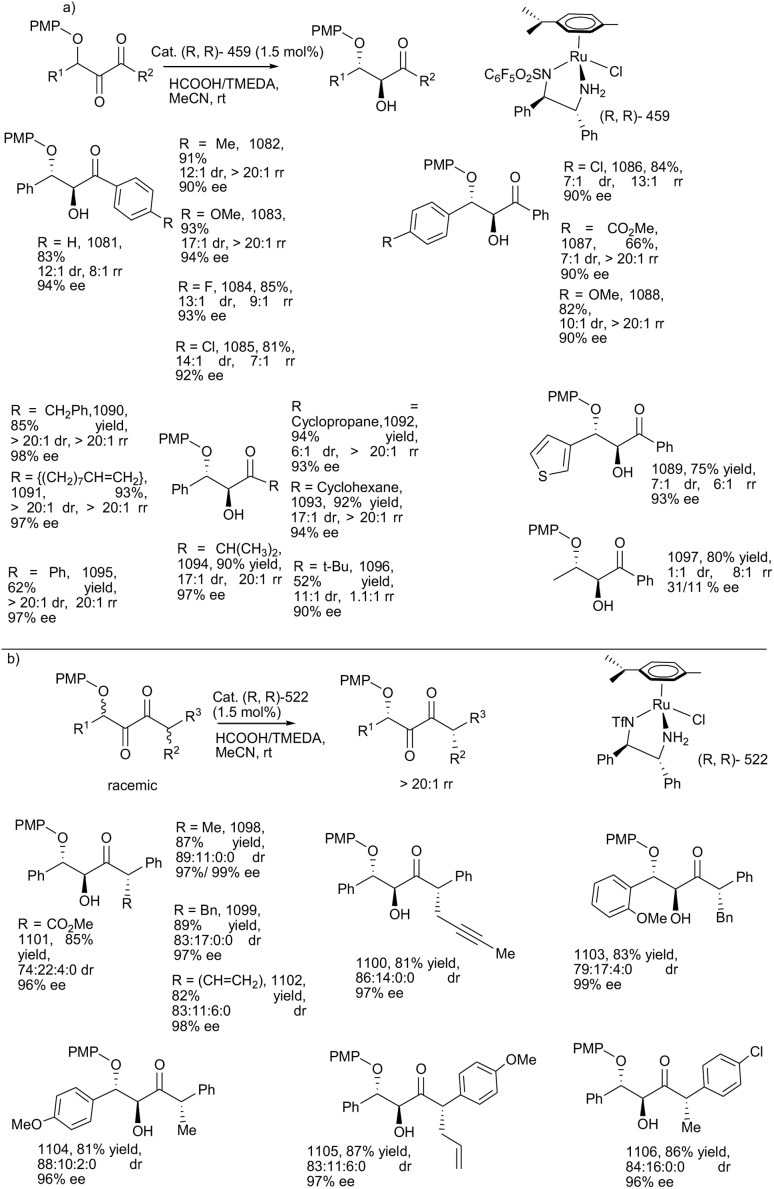
Substrate scope of diketones. Reproduced from ref. 134 with permission from the American Chemical Society. Copyright 2024.

Fang *et al.*^134^ (2024) reported the ATH of unsymmetrical racemic diketones using a ruthenium catalyst, demonstrating that aryloxy-substituted 1,2-diketones can be efficiently converted into anti-dihydroxyketone derivatives (Scheme 64a). Substrates bearing electron-donating groups such as methyl (Me) and methoxy (OMe) on the phenyl ring (*R*^2^ position) afforded the corresponding anti-dihydroxyketones in excellent yields (91–93%) with high enantioselectivities (90–94% ee) using the (*R*,*R*)-459 catalyst (1081–1083). In contrast, electron-withdrawing substituents such as fluorine (F) and chlorine (Cl) delivered slightly reduced yields (81–85%) while maintaining high ee (92–93%; 1084, 1085). Variation at the *R*^1^ position, including phenyl rings bearing –Cl, –CO_2_Me, and –OMe substituents, also gave the corresponding products in good to excellent yields (66–84%) with around 90% ee (1086–1088). The reaction tolerated heteroaryl systems as well, with thiophene-containing substrates delivering the desired products (1089). Alkyl-substituted *R*^2^ groups, including primary, secondary, and tertiary alkyl chains, were also well accommodated, giving products with excellent ee (1090–1096). Notably, sterically demanding *tert*-butyl substituents influenced the reaction outcome, affecting diastereo- and regioselectivity to some extent, highlighting the role of steric effects in substrate control. When *R*^1^ was a methyl group, the corresponding alcohol product was obtained in very good yield and ee (1097). The absolute configuration of compound 1081 was confirmed by single-crystal X-ray crystallography. Interestingly, racemic diketones containing two stereogenic centers underwent a double DKR under catalyst (*R*,*R*)-522, leading to highly functionalized products bearing three stereocenters (Scheme 64b). Alkyl substituents at *R*^2^, such as methyl, benzyl, alkyne, ester, and allyl groups, were well tolerated, providing products in excellent yields and outstanding enantioselectivities (96–98% ee; 1098–1102). Variation at *R*^1^ with methoxy-substituted phenyl rings also afforded good yields (81–83%) and high ee (96–99%; 1103–1104).

**Scheme 65:**
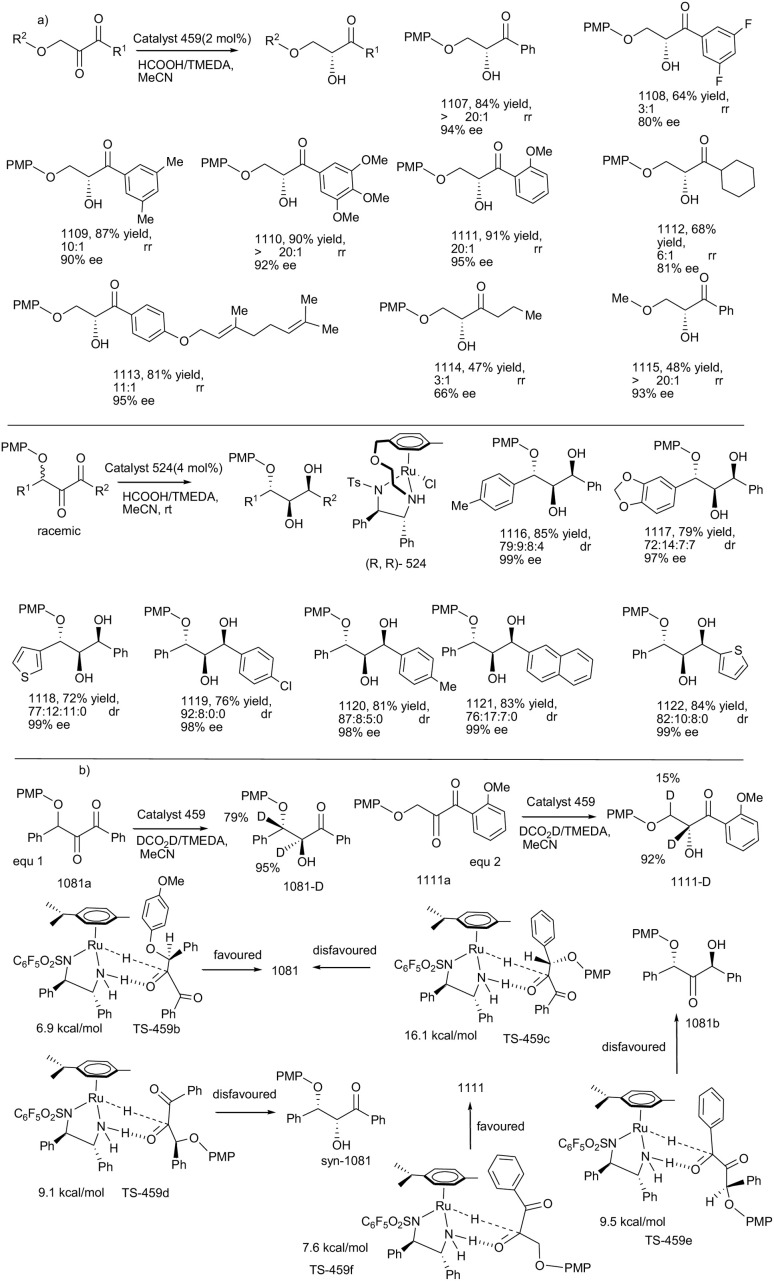
(a) Substrate scope of diketones. (b) DFT calculations on the ATH. Reproduced from ref. 134 with permission from the American Chemical Society. Copyright 2024.

*Para*-substituted aryl groups, including –OMe and –Cl, gave products in 86–87% yield with good diastereocontrol and excellent ee (96–97%; 1105, 1106). A further extension to α-diketones bearing OPMP (O-*para*-methoxyphenyl) protecting groups revealed a broad substrate scope (Scheme 65a). These systems delivered anti-diol products in good yields (64–87%) with high enantioselectivities (80–95% ee; 1107–1111). Cyclohexyl-substituted substrates showed moderate reactivity (68% yield; 81% ee; 1112), while replacement of OPMP with OMe led to reduced yield but maintained good ee (48% yield; 93%; 1115). Additionally, enantioenriched triols were synthesized using catalyst 524, affording products with excellent yields (72–85%) and outstanding enantioselectivities (97–99% ee; 1116–1118). Substrates bearing phenyl, naphthyl, and thienyl groups at *R*^2^ also performed well, giving products in high yield and excellent ee (98–99%; 1119–1122). Mechanistic investigations supported the involvement of a DKR pathway. Deuterium-labeling experiments using DCOOD revealed significant incorporation at both *α*- and *β*-positions (95% and 79%, respectively) for 1081a, confirming dual proton/hydride transfer processes (Scheme 65b). In contrast, a related substrate (1111a) showed preferential deuterium incorporation at the *α*-position (92%), indicating higher acidity and reactivity at this site compared to the *β*-position. Theoretical calculations (DFT) provided further insight into stereocontrol. For substrate 1081a, four competing transition states were analyzed. The most favorable pathway (TS-459b, Δ*G* = 6.9 kcal mol^−1^) is stabilized by the optimal orientation of the OPMP group toward the catalyst arene, minimizing steric repulsion and enabling a key hydrogen bond between the ligand N–H and OPMP oxygen. In contrast, sterically congested TS-459c exhibits a significantly higher activation barrier (16.1 kcal mol^−1^). Alternative transition states (TS-459d and TS-459e) show intermediate barriers (9.1 and 9.5 kcal mol^−1^) due to less favorable alignment and weaker stabilizing interactions. For OPMP-containing α-diketones, additional hydrogen bonding interactions involving the OPMP –CH_2_ and the SO_2_ oxygen of the catalyst further stabilize the preferred transition state (TS-459f, Δ*G* = 7.6 kcal mol^−1^). However, alternative orientations reduce interaction strength and increase activation barriers (8.6–10.2 kcal mol^−1^), leading to less favorable reaction pathways. Overall, this study demonstrates how subtle steric and hydrogen-bonding interactions in the transition state govern both reactivity and stereochemical outcome in ruthenium-catalyzed ATH of complex diketone systems.

**Scheme 66:**
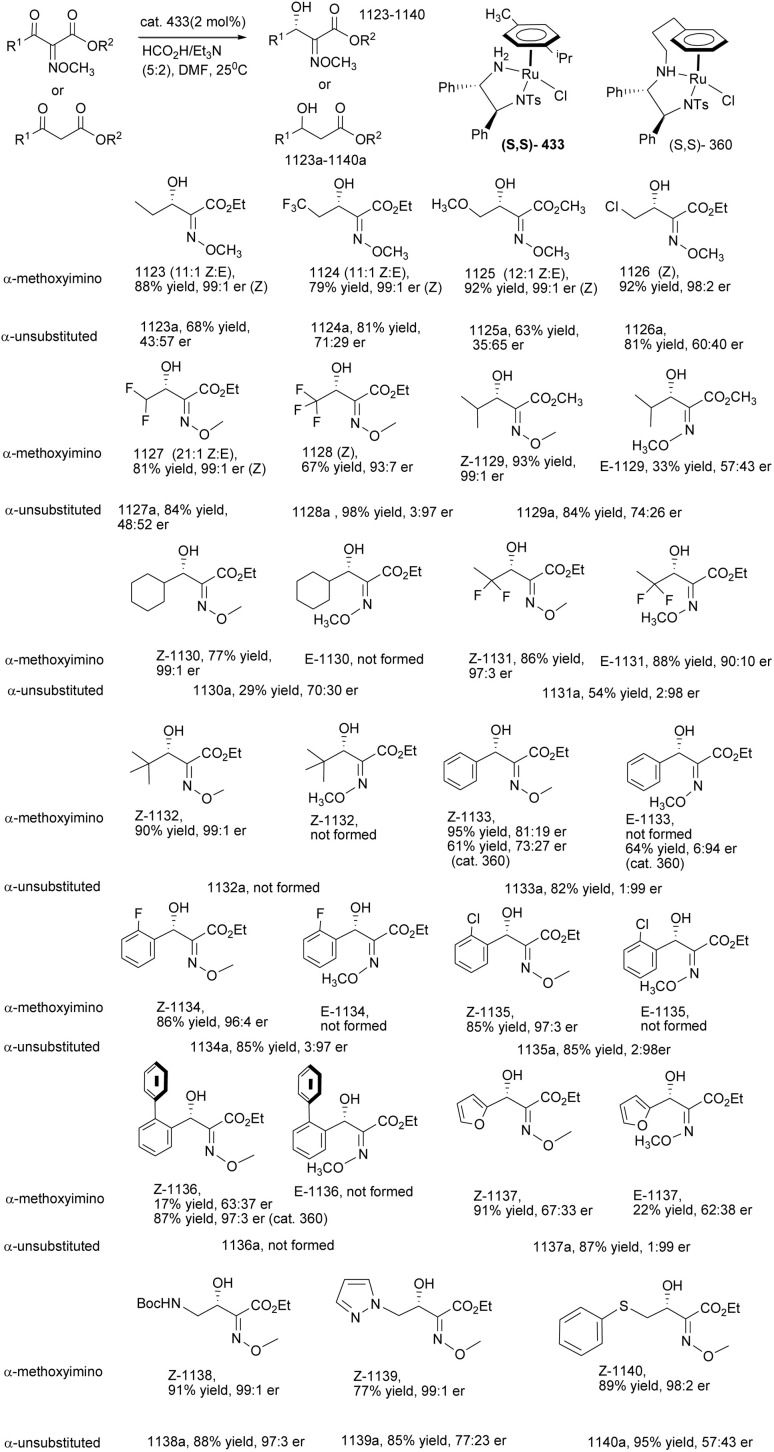
Scope of α-methoxyimino-β-keto esters examined using the complex (*S*,*S*)-433, shown alongside results with α-unsubstituted-β-keto esters. Reproduced from ref. 135 with permission from the American Chemical Society. Copyright 2024.

Jakub Švenda^135^ (2024) reported the ATH of α-methoxyimino-β-keto esters employing the Noyori–Ikariya catalyst [RuCl(η^6^-*p*-cymene) ((*S*,*S*)-TsDPEN)] (433) using formic acid, triethylamine, and dimethylformamide at 25 °C. Under these mild conditions, the reaction carried out with excellent stereoselectivity produced the *Z*-isomer products (1123–1140) with high er (93 : 7 to 99 : 1) (Scheme 66). In contrast, α-unsubstituted β-keto esters exhibited significantly lower reactivity and poorer selectivity (1123a–1140a), highlighting the crucial role of the methoxyimino group. Substrates bearing strongly electron-withdrawing groups such as CF_3_ and 1,1-difluoroethyl exhibited interesting divergent selectivity, giving both isomers with high but distinct enantioselectivities (*Z*-1128: 93 : 7 er *vs.*1128a: 3 : 97 er; *Z*-1131: 97 : 3 er *vs.*1131a: 2 : 98 er). Alkyl-substituted substrates also performed well: the isopropyl-substituted ketone delivered the *Z*-isomer in 93% yield with excellent er (99 : 1), whereas the corresponding *E*-isomer was formed in lower yield and selectivity (57 : 43 er) even after extended reaction time. Similarly, cyclohexyl- and *tert*-butyl-substituted derivatives predominantly afforded the *Z*-products (*Z*-1130, *Z*-1132) with high efficiency, while formation of the *E*-isomers was minimal (<5%). Aryl-substituted α-methoxyimino-β-keto esters were also smoothly reduced to the corresponding *Z*-alcohols (*Z*-1133–1135) with good to excellent er, while the *E*-isomers were obtained only in trace amounts. In contrast, substrates containing a 2-furyl group showed reduced stereoselectivity (*Z*-1137: 67 : 33 er; *E*-1137: 62 : 38 er), indicating the influence of heteroaryl electronics on transition-state control. For α-unsubstituted β-aryl β-keto esters, excellent er was observed (1133a–1135a, 1137a: 97 : 3 to 99 : 1 er), despite reduced overall reactivity. A 2-biphenylyl substrate exhibited poor performance, giving low yield (17% after 6 days) and moderate er (63 : 37). Notably, substrates bearing *N*-boc-aminomethyl, pyrazolylmethyl, and phenylthiomethyl substituents were efficiently reduced to the corresponding *Z*-products (1138–1140) with excellent er (98 : 2 to 99 : 1). The α-unsubstituted *N*-boc-aminomethyl ketone also showed good reactivity and high stereoselectivity (1138a: 97 : 3 er). DFT studies suggest that the *Z*-configuration of the methoxyimino group plays a decisive role in directing both reactivity and stereoselectivity. This group enhances substrate–catalyst interactions and stabilizes the preferred transition state, thereby governing the observed high enantioselectivity in the ATH process.

**Scheme 67:**
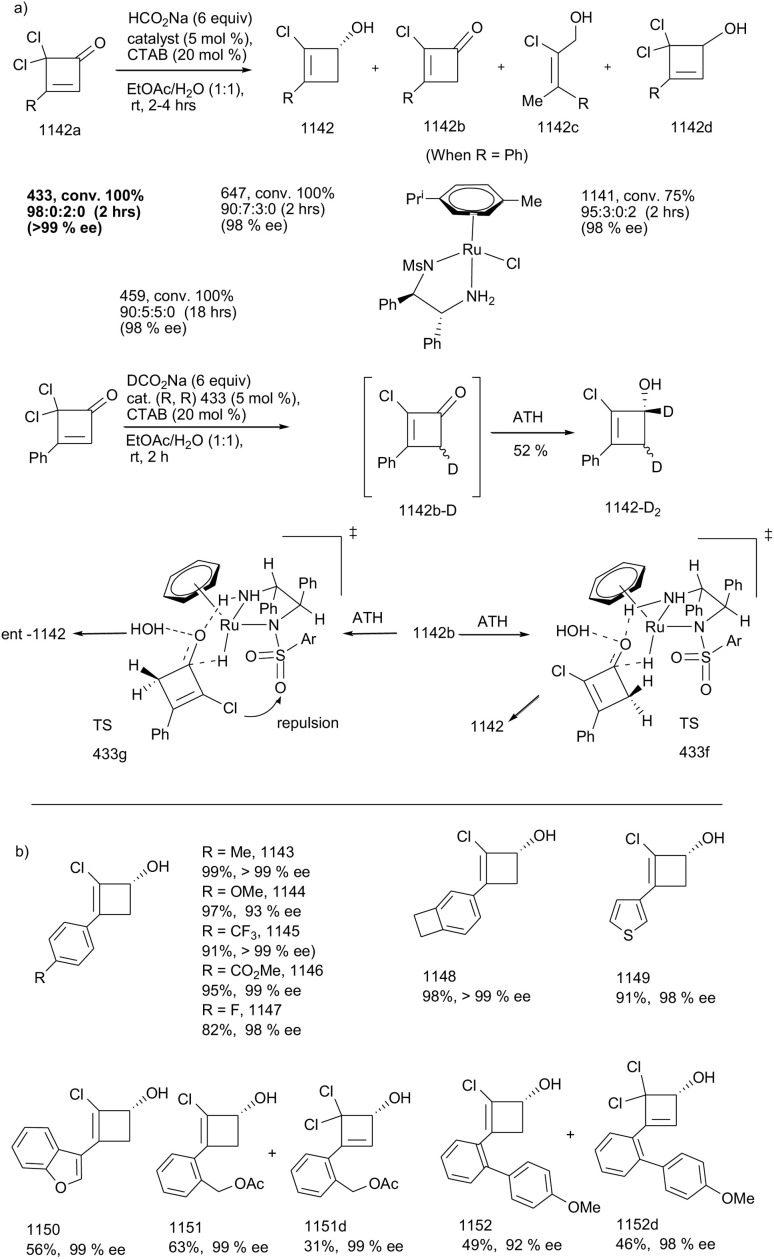
(a) Optimized reaction condition using different catalyst and mechanism. (b) Scope of the allylic reduction-ATH cascade. Reproduced from ref. 136 with permission from Wiley-VCH. Copyright 2024.

Christophe Meyer *et al.*^136^ (2024) reported an ATH of *gem*-dichloro-cyclobutenones using a Noyori–Ikariya-type ruthenium catalyst. In this work, a reductive allylic substitution followed by ATH enabled the efficient synthesis of 2-chlorocyclobuten-1-ols with excellent enantioselectivity (Scheme 67a). Using 5 mol% of the (*R*,*R*)-433 catalyst, dichlorocyclobutenone (1142a) was converted to 2-chlorocyclobuten-1-ol (1142) in the presence of a HCO_2_H/Et_3_N hydrogen donor system in ethyl acetate at room temperature within 3 hours. The reaction proceeded with high selectivity, giving a product distribution of 98 : 0 : 2 : 0 for 1142/1142b/1142c/1142d. Mechanistically, *in situ* generation of a Ru–H species from [Ru]–Cl and HCO_2_H/Et_3_N promotes chloride elimination to form 2-chlorocyclobutenone (1142b), which subsequently undergoes ATH *via* transition state TS-433f to afford the major chiral alcohol product. Several reaction parameters were optimized to improve yield and enantioselectivity. A mixed aqueous–organic solvent system containing sodium formate and cetyltrimethylammonium bromide (CTAB) provided ee values >99%. Alternative solvents such as THF and dichloromethane also gave satisfactory results. Among different catalysts tested, 647 (mesitylene-based) and 1141 showed good activity, while catalyst 459 selectively produced intermediate 1142b; however, extending the reaction time to 18 hours led to the formation of the desired product 1142 as the major product. A gram-scale reaction confirmed the robustness of the methodology without loss of enantioselectivity. Deuterium-labelling experiments using DCO_2_Na resulted in the formation of dideuterated cyclobutanol (1142-D_2_), supporting the proposed mechanism and the preference for transition state TS-433f over the less favorable TS-433g (Scheme 67a). The scope of the reaction was further extended to various β-substituted dichlorocyclobutenones (Scheme 67b). Substrates bearing electron-donating groups such as methoxy and methyl at the *para* position afforded products 1143 and 1144 in 99% and 97% yields with >99% and 93% ee, respectively. Electron-withdrawing substituents, including CF_3_, CO_2_Me, and F, also delivered excellent enantioselectivities (>98–99% ee) with good yields (82–95%) for products 1145–1147. Disubstituted phenyl systems gave a high yield (98%) and excellent ee (>99%) for product 1148. Heteroaryl-substituted substrates such as thiophene and benzofuran were also well tolerated, affording products 1149 and 1150 in 91% and 56% yield with 98–99% ee. In contrast, *ortho*-substituted substrates led to mixtures of chlorocyclobutenol and dichlorocyclobutenol products (1151–1152 and 1151d–1152d), though still with high ee (92–99%). In summary, this study expands the scope of highly strained cyclobutenone substrates in ATH chemistry and provides an efficient approach for the synthesis of valuable chiral cyclobutene derivatives with excellent stereocontrol.

**Scheme 68:**
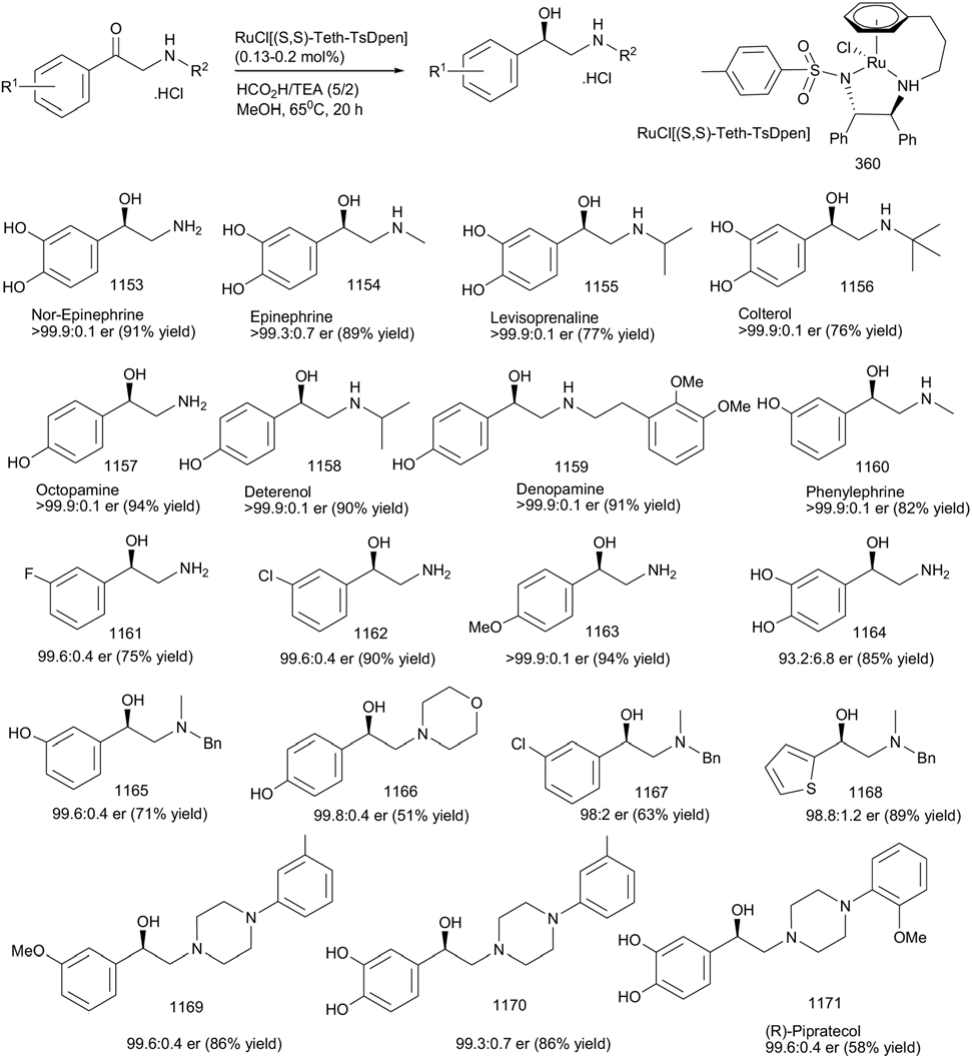
Reduction of unprotected amino ketones using a ruthenium chiral catalyst. Reproduced from ref. 137 with permission from the American Chemical Society. Copyright 2024.

The reduction of unprotected amino ketones using a chiral ruthenium catalyst was first reported by Mangunuru and Senanayake^137^ in 2024. In this study, amino alcohols featuring phenolic and catecholic motifs, such as norepinephrine and adrenaline, were targeted due to their importance in medicinal chemistry. A range of diamine-based ruthenium catalysts was evaluated for the asymmetric reduction of α-amino ketone hydrochloride salts, leading to the formation of chiral 1,2-amino alcohol derivatives. Among the catalysts investigated, a tethered ruthenium complex showed the best performance, delivering quantitative conversion (100%) with excellent er (99.3 : 0.7). A wide range of aromatic and heteroaryl-substituted α-amino ketones were effectively reduced (Scheme 68). Using this method, norepinephrine was synthesized in 91% yield with outstanding er (>99.9 : 0.1). The same catalytic system was successfully applied to the synthesis of levisoprenaline, affording the product in 77% yield with excellent enantiomeric purity (>99.9 : 0.1 er). Additionally, *para*-hydroxyphenyl amino alcohol (octopamine) was obtained in high yield (94%) with exceptional er (>99.9 : 0.1), representing a significant improvement over previously reported enzymatic approaches. The presence of hydroxyl substituents at the 3- and 4-positions of the aromatic ring was found to play a key role in enhancing both reactivity and stereocontrol. Furthermore, fluoro-, chloro-, and methoxy-substituted derivatives were also successfully synthesized, compound 1163 showing improved yields and enantioselectivity compared to earlier literature reports. The methodology was further extended to heterocyclic systems, including amino alcohol-containing piperazine derivatives, which were obtained with excellent yields and enantioselectivities. The same conditions were successfully applied to compounds 1170 and the vasodilator (*R*)-pipratecol (1171), demonstrating the broad applicability of the protocol. Importantly, the synthesis of norepinephrine and epinephrine were also demonstrated on a 10 gram scale without loss of efficiency or stereocontrol. Overall, this study represents a green and highly practical catalytic strategy for the synthesis of pharmaceutically important chiral amino alcohols, offering advantages in terms of operational simplicity, safety, scalability, and cost-effectiveness.

**Scheme 69:**
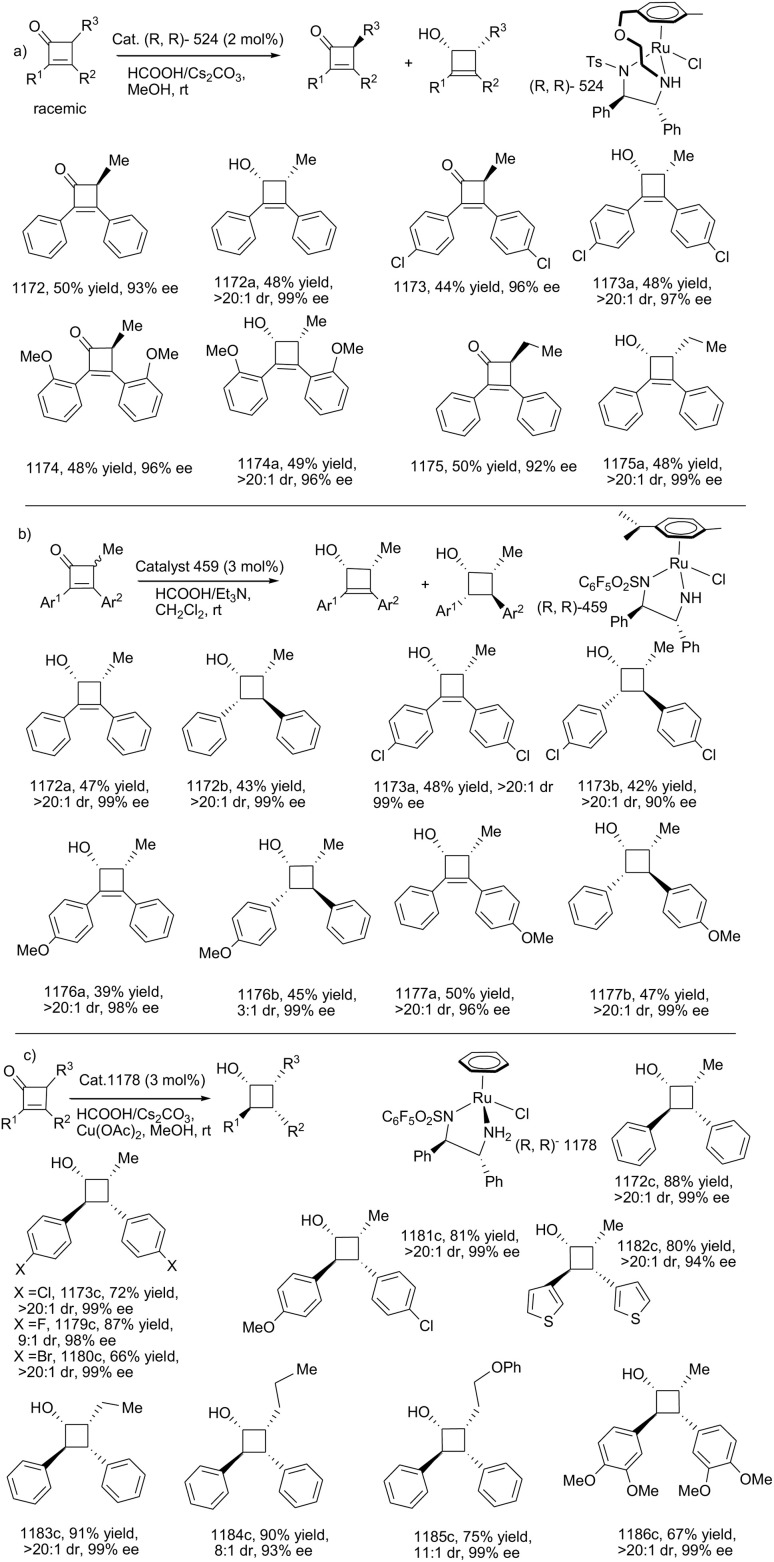
(a) Substrate scope of kinetic resolution of cyclobutenone. (b) Substrate scope of parallel kinetic resolution of cyclobutenones. (c) Substrate scope of DKR of cyclobutenones. Reproduced from ref. 78 with permission from Wiley-VCH. Copyright 2025.

**Scheme 70:**
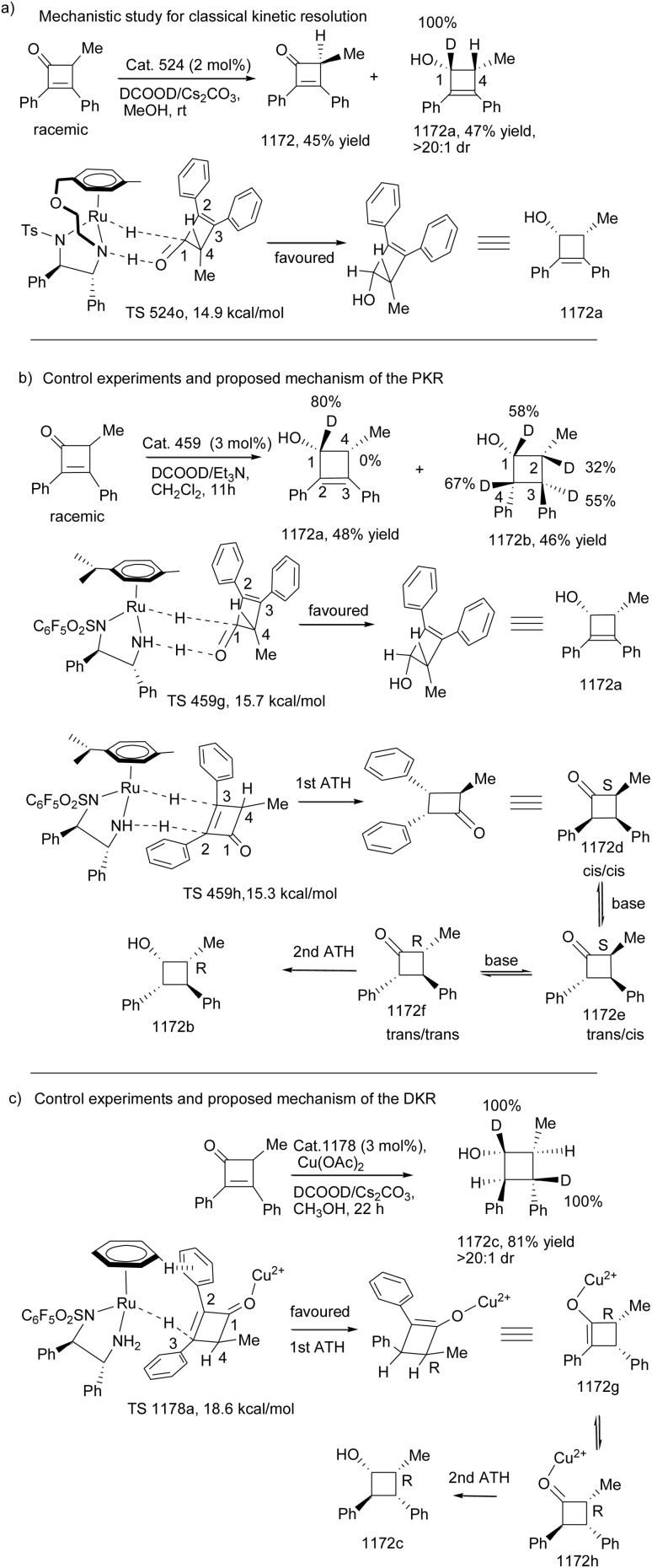
(a–c) Control experiments and proposed mechanism. Reproduced from ref. 78 with permission from Wiley-VCH. Copyright 2025.

Fang *et al.*^78^ (2025) demonstrated that ATH-based diversified kinetic resolution of 2,3-diarylcyclobutenones, catalyzed by ruthenium complexes, affords highly enantioenriched products with excellent efficiency and selectivity. Using catalyst 524 in the presence of a racemic substrate and a combination of formic acid (HCO_2_H), cesium carbonate (Cs_2_CO_3_), and methanol, the reaction furnished the monohydrogenated product 1172 with 50% yield and 93% ee, along with the corresponding alcohol product 1172a in 48% yield and 99% ee *via* classical kinetic resolution (Scheme 69a). Substrates bearing chloro and methoxy substituents on the phenyl ring also delivered products 1173/1173a and 1174/1174a with excellent ee (96–97%). Replacement of a methyl group with an ethyl substituent maintained similarly high efficiency and stereocontrol (1175/1175a). When catalyst 459 was employed in the presence of formic acid and triethylamine (Et_3_N) in dichloromethane, parallel kinetic resolution (PKR) was observed, furnishing products 1172a (47% yield, 99% ee) and 1172b (43% yield, 99% ee) (Scheme 69b). Dichloro-substituted substrates also exhibited high stereoselectivity, giving 1173a with 99% ee and 1173b with 90% ee. Methoxy-substituted derivatives provided products 1176a/1176b and 1177a/1177b with excellent ee (96–99%). Further, using catalyst 1178 with HCO_2_H/Cs_2_CO_3_ and Cu(OAc)_2_ in methanol enabled DKR, affording alcohol 1172c in 88% yield with 99% ee (Scheme 69c). Electron-withdrawing substituents such as chloro, fluoro, and bromo groups afforded products 1173c, 1179c, and 1180c in good to very good yields (66–87%) with excellent ee (98–99%). Mixed substituent systems (Cl and OMe) also performed well, giving 1181c in 81% yield with 99% ee. Thiophene-containing cyclobutenones and various alkyl-substituted derivatives (Me, Et, Pr, and OPhEt) were also well tolerated, consistently delivering high enantioselectivity (1182c–1185c). Mechanistic investigations using deuterium-labelling experiments (DCO_2_D) revealed distinct pathways for classical kinetic resolution, PKR, and DKR. In classical kinetic resolution, no deuterium incorporation was observed in product 1172, whereas selective incorporation at the C1 position occurred in 1172a (Scheme 70a). DFT calculations supported a bifunctional mechanism involving simultaneous hydride transfer from the Ru center and proton transfer from the ligand *via* transition state TS-524o (activation barrier 14.9 kcal mol^−1^). This transition state is stabilized by CH…O hydrogen bonding between the substrate and the SO_2_ group of the catalyst, along with additional CH–π interactions involving the arene ligand. In the case of PKR, deuterium incorporation at the C1 position of 1172a and multiple positions in 1172b confirmed competing pathways (Scheme 70b). The reaction proceeds through closely related transition states TS-459g and TS-459h, with activation barriers of 15.7 and 15.3 kcal mol^−1^, respectively, leading to both 1,2- and 1,4-reduction pathways followed by isomerization. For DKR, deuterium labelling indicated selective incorporation at the C1 and C3 positions of 1172c (Scheme 70c). The mechanism involves Cu(OAc)_2_-assisted activation and proceeds through transition state TS-1178a (18.6 kcal mol^−1^), followed by sequential reduction and protonation steps. Additional stabilization arises from CH…O hydrogen bonding with the SO_2_ group and edge-to-face π–π interactions between the catalyst and substrate aromatic rings. Overall, this study highlights the broad scope of ruthenium-catalyzed ATH systems in promoting classical, parallel, and DKR with high efficiency, while DFT calculations indicate the crucial role of non-covalent interactions in governing selectivity.

**Scheme 71:**
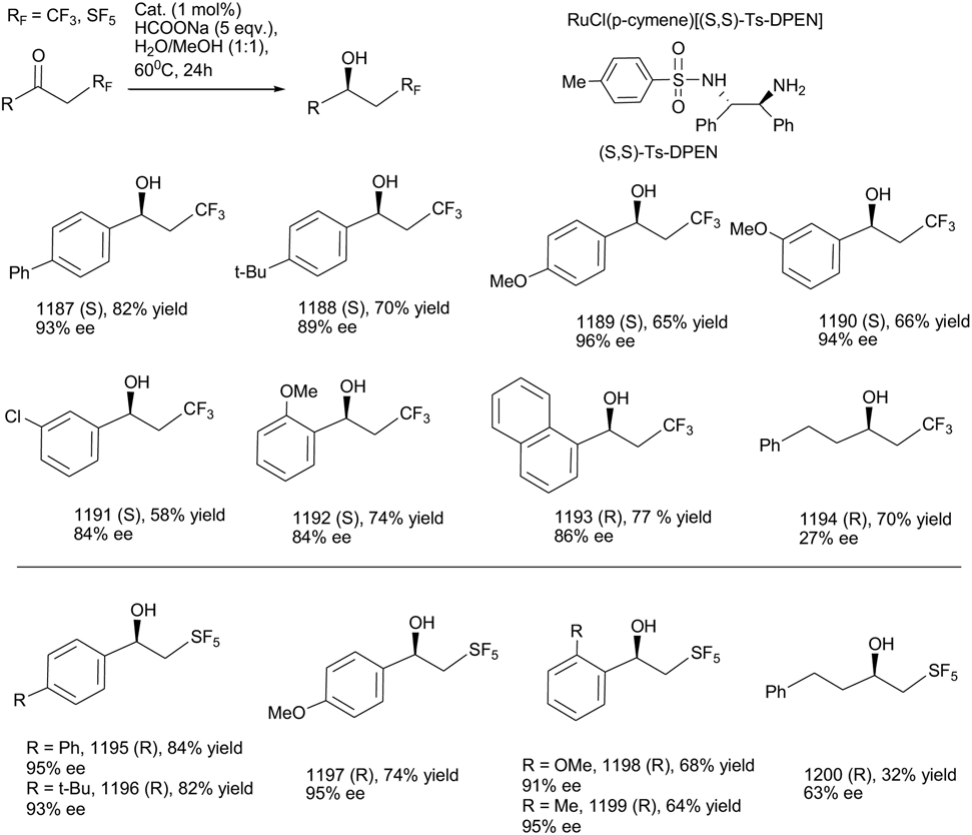
Reaction scope of the Noyori–Ikariya ATH of α-CF_3_ and α-SF_5_ ketones. Reproduced from ref. 138 with permission from the American Chemical Society. Copyright 2025.

The study by Paquin *et al.*^138^ (2025) investigated the ATH of α-CF_3_- and α-SF_5_-substituted ketones using the Noyori–Ikariya catalyst system. This transformation afforded the corresponding β-CF_3_ and β-SF_5_ alcohols in good to excellent yields (up to 84%) with high enantioselectivities, reaching up to 96% ee (Scheme 71). The absolute configuration of the products was unambiguously established by single-crystal X-ray diffraction (SCXRD) analysis. The reactions were performed using the catalyst [RuCl(η^6^-*p*-cymene)((*S*,*S*)-TsDPEN)] in the presence of sodium formate (HCOONa) as the hydrogen source in a water/methanol solvent system at 60 °C for 24 hours. Under these optimized conditions, α-CF_3_ ketones bearing a *para*-substituted phenyl ring afforded the corresponding alcohol with 82% yield and 93% ee (1187). A systematic evaluation of substituent effects on the aromatic ring revealed that both electron-donating and electron-withdrawing groups significantly influence catalytic performance. The phenyl ring containing the electron-donating *tert*-butyl (*t*-Bu) group and the electron-withdrawing chloro (–Cl) group, the corresponding alcohol products had yields of 70% and 58%, with enantioselectivities of 89% and 84%, respectively (1188, 1191). Methoxy-containing functional substrates also produced alcohol products with good yields (65–74%) and very good enantioselectivities (1189, 1190, 1192, 84–96% ee). Additionally, ketones containing naphthyl groups tolerated this reaction, yielding products with a 77% yield and 86% ee (1193). In contrast, aliphatic ketones exhibited significantly reduced performance, giving only 70% yield and low ee (27%, 1194), highlighting the importance of aromatic stabilization in controlling stereoselectivity. A similar trend was observed for α-SF_5_-functionalized ketones. Substrates bearing phenyl and *tert*-butyl substituents afforded the desired alcohols in 84% and 82% yield with 95% and 93% ee, respectively (1195, 1196). Methoxy-substituted SF_5_-ketones also showed excellent performance, delivering products with high enantioselectivities of 91–95% ee (1197, 1198). Methyl-substituted derivatives afforded 95% ee (1199), whereas alkyl-substituted ketones exhibited reduced reactivity, giving only 32% yield with moderate ee (63%, 1200). Overall, this study demonstrates that both electronic and steric properties of substituents play a decisive role in controlling reactivity and enantioselectivity in ATH of highly functionalized α-CF_3_ and α-SF_5_ ketones under Noyori–Ikariya catalysis.

## Understanding the mechanism and origin of enantioselectivity in ATH utilizing the Noyori–Ikariya catalyst

**Scheme 72:**
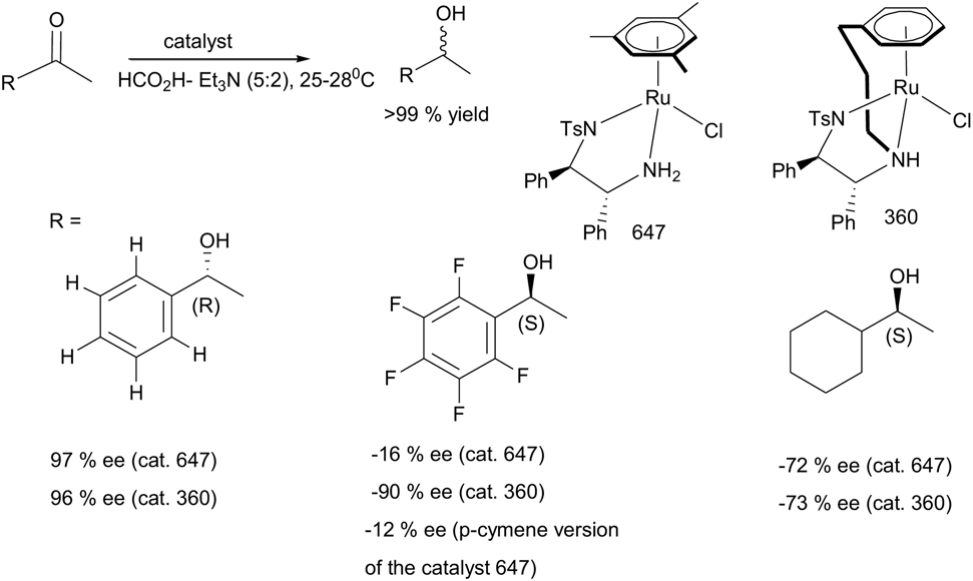
Reported asymmetric transfer hydrogenation of ketone. Reproduced from ref. 85 with permission from the American Chemical Society. Copyright 2021.

**Fig. 2:**
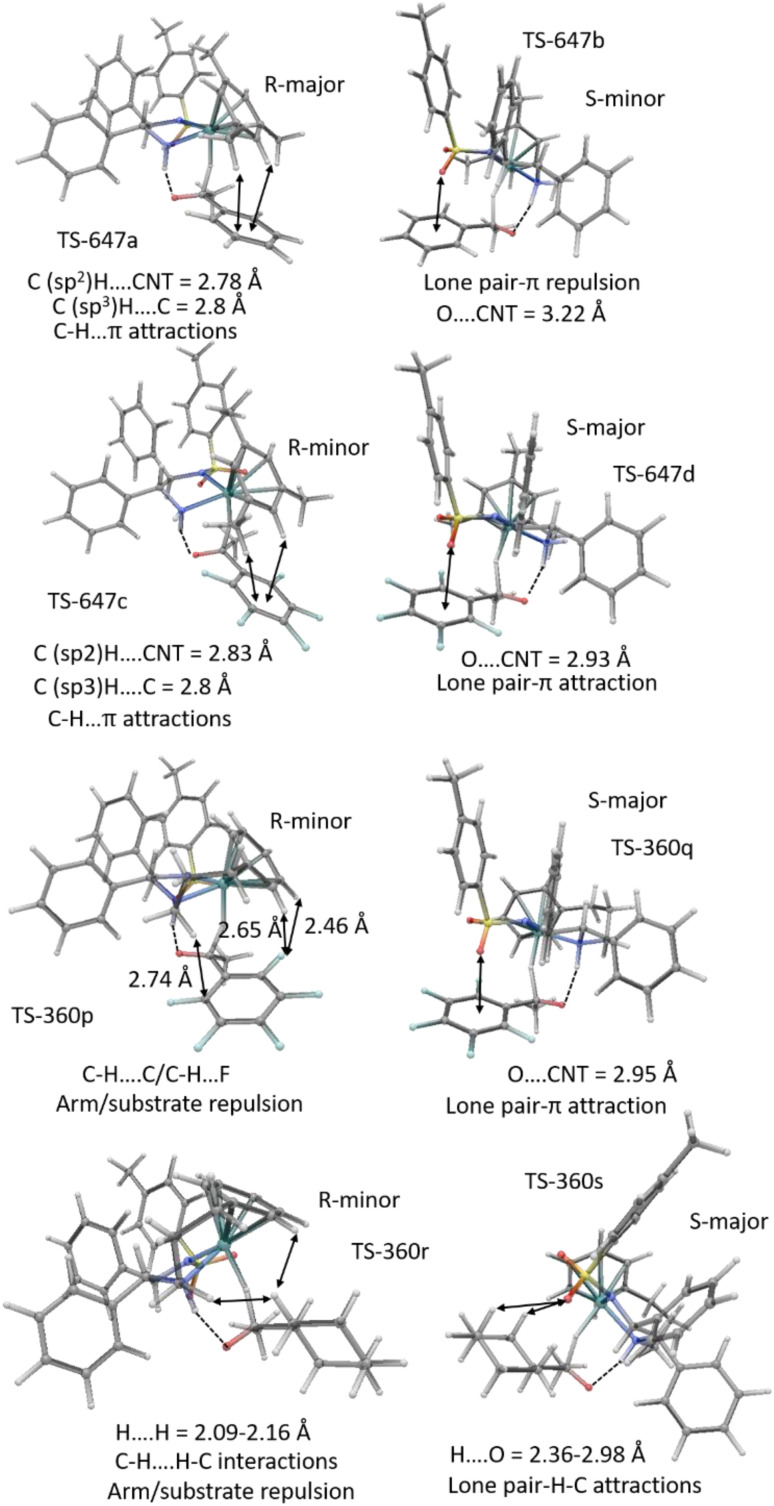
Optimized transition states leading to enantiomers of 1-phenylethanol (cat. (*R*,*R*)-647, top), 1-(2,3,4,5,6-pentafluorophenyl)ethanol (cat. (*R*,*R*)-647 and (*R*,*R*)-360, middle), and 1-cyclohexane-1-ethanol (cat. (*R*,*R*)-360, bottom). Reproduced from ref. 85 with permission from the American Chemical Society. Copyright 2021.

P. A. Dub *et al.*^85^ (2021) demonstrated that enantioselectivity in Noyori–Ikariya-type TH is predominantly governed by interactions between the tethered η^6^-arene ligand and the SO_2_ group of the catalyst with the substrate. These structural features play a decisive role in controlling the absolute configuration of the product. In particular, a range of non-covalent interactions—including CH–π, C–H⋯H–C, lone pair–π, and lone pair⋯H–C interactions—were shown to either stabilize or destabilize competing diastereomeric transition states, thereby directly influencing enantioselectivity. The authors investigated the ATH reduction of acetophenone, pentafluoroacetophenone, and 1-cyclohexylethanone using Noyori–Ikariya catalysts (*R*,*R*)-647 and (*R*,*R*)-360 in isopropanol (Scheme 72). In the case of acetophenone, both catalysts furnished (*R*)-1- phenylethanol with excellent ee (96–97%), indicating a strong preference for the formation of the *R*-enantiomer. In contrast, when pentafluoro acetophenone was employed as the substrate, a complete reversal in stereoselectivity was observed, affording alcohols with −16% ee for 647 and −90% ee for 360. Similarly, reduction of 1-cyclohexylethanone yielded (*S*)-1-cyclohexylethanol with ee values of −72% and −73% for 647 and 360, respectively. To rationalize these observations, density functional theory (DFT) calculations combined with non-covalent interaction (NCI) analyses were performed. For acetophenone, the transition states leading to the *R*-product (TS-647a and TS-647b) were found to be comparably stabilized through CH–π interactions, as reflected by similar interatomic distances (Fig. 2). By contrast, in the *S*-selective pathway involving pentafluoro acetophenone, pronounced stabilization was observed due to lone pair–π interactions between the oxygen atom of the catalyst's SO_2_ group and the electron-deficient aromatic ring of the substrate (TS-647d). In comparison, acetophenone exhibited weaker or slightly repulsive lone pair–π interactions (TS-647c), resulting in reduced stabilization of the corresponding transition state and thereby favoring the opposite enantiomer. This difference accounts for the observed inversion of configuration upon substrate modification. For pentafluoro acetophenone, both catalysts preferentially delivered the *S*-product owing to enhanced stabilization of the *S*-diastereomeric transition state. Notably, (*R*,*R*)-360 exhibited significantly higher enantioselectivity than (*R*,*R*)-647, which was attributed to increased steric destabilization in the competing transition state arising from the bulky tethered η^6^-arene ligand. This steric congestion leads to greater energetic discrimination between competing pathways. Additionally, restricted rotational freedom of the aromatic moiety further amplifies transition-state destabilization in the disfavored pathway, thereby enhancing enantioselectivity. Overall, this study highlights how subtle variations in non-covalent interactions, together with catalyst steric environment, play a crucial role in dictating stereochemical outcomes in Noyori–Ikariya-type ATH reactions.

**Scheme 73:**
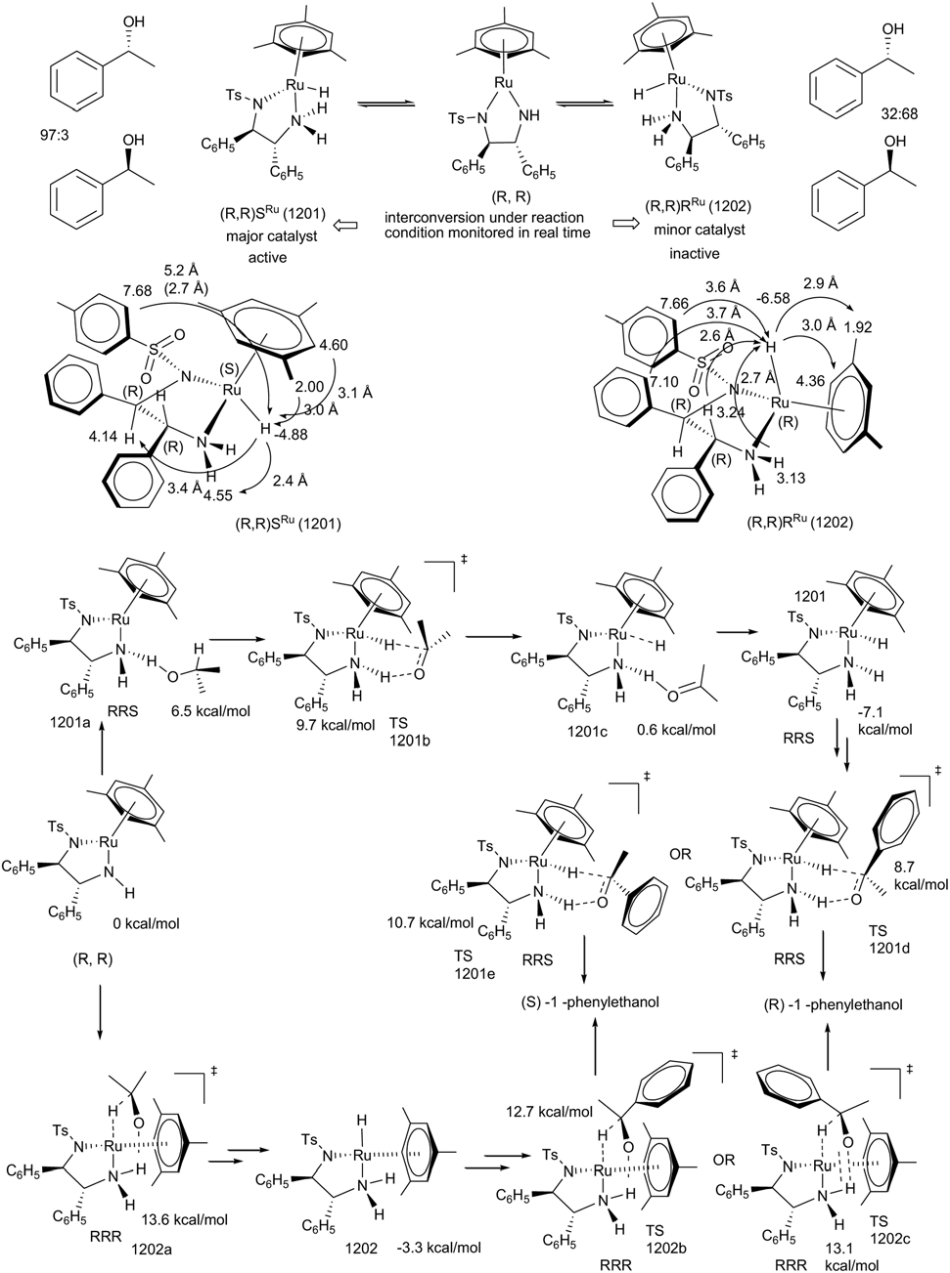
(a) ATH reaction of Noyori's TsDPEN catalyst and DFT optimized solution structures and experimental ^1^H NMR chemical shifts (C_6_D_6_) of hydride complexes (*R*,*R*)SRu (1201) and (*R*,*R*)RRu (1202) showing observed NOE interactions and contact distances. (b) Proposed mechanism and calculated ground and transition state energies (kcal mol^−1^). Reproduced from ref. 86 with permission from the American Chemical Society. Copyright 2021.

U. Hintermair *et al.*^86^ (2021) reported the detection of low-concentration ruthenium hydride species in the ATH reaction using FlowNMR spectroscopy in combination with Noyori's TsDPEN catalyst. Both major and minor hydride species were successfully identified, and kinetic measurements were performed to quantify their relative populations during the catalytic cycle. In the catalytic reaction, [(mesitylene)Ru((*R*,*R*)-TsDPEN)], in the presence of ketone and isopropanol, produced major species assigned as *S*-Ru (1201) and a minor isomeric species, *R*-Ru (1202), was identified through NOE-NMR experiments (Scheme 73). During the reaction, interconversion between these species took place, leading to a prominent hydride peak observed at −4.88 ppm (*S*-Ru) in the NMR spectra. Density functional theory (DFT) calculations supported the NMR findings, demonstrating that (*R*,*R*)*S*-Ru (1201) represents the most stable structure, characterized by a syn-coplanar configuration of NH–Ru–H. Mechanistically, the precursor complex (*R*,*R*)-Ru reacts with isopropanol to generate (*R*,*R*)-*S*-Ru (1201) *via* transition state TS-1201b, which is energetically more favorable by 3.9 kcal mol^−1^ compared to the alternative pathway leading to (*R*,*R*)-*R*-Ru (1202) through transition state TS-1202a. Furthermore, (*R*,*R*)-*S*-Ru exhibits a calculated stability of −7.1 kcal mol^−1^, making it more stable by 3.8 kcal mol^−1^ relative to (*R*,*R*)-*R*-Ru, which has a stability of −3.3 kcal mol^−1^. Overall, these results demonstrate that (*R*,*R*)-*S*-Ru is both kinetically and thermodynamically favored, consistent with a lock-and-key model governing catalyst–substrate interactions in the ATH process.

**Scheme 74:**
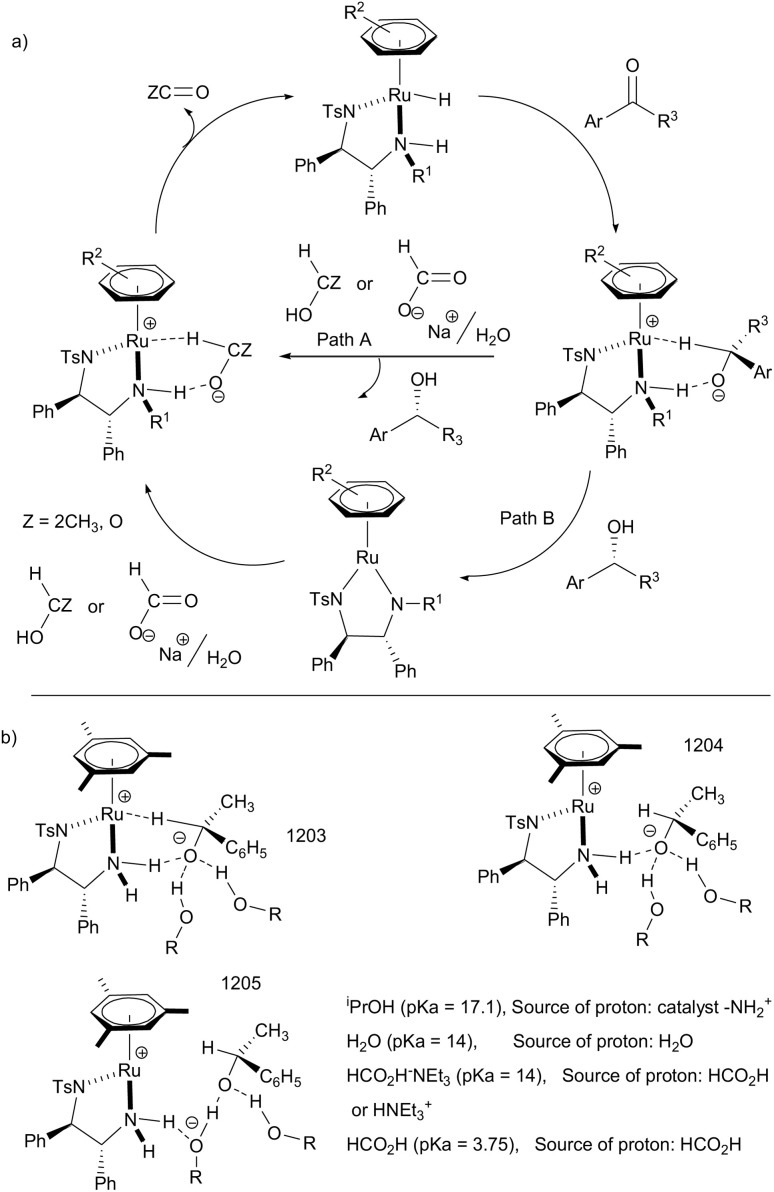
(a) Mechanism of TH of the Noyori–Ikariya catalyst. (b) Reaction core starting structures for MD runs in this work (R = ^*i*^Pr, HCO_2_, H) and source of protons in the Noyori–Ikariya reaction. Reproduced from ref. 87 with permission from the American Chemical Society. Copyright 2022.

The mechanism of ATH using the Noyori–Ikariya catalyst has been extensively investigated over the past three decades.^139–144^ Despite significant progress, certain mechanistic aspects remain unresolved. The initial step involving hydride transfer is now well established (Scheme 74, Path A). However, the subsequent proton transfer step is still a subject of ongoing debate (Scheme 74, Path B). Pavel Dub *et al.*^87^ (2022) proposed two possible pathways for proton delivery to the substrate: one involving transfer from the N–H bond of the catalyst and the other from the O–H bond of the reagent or solvent. Common proton sources in ATH reactions include propan-2-ol, a formic acid/triethylamine mixture (HCO_2_H–NEt_3_, 5 : 2), and water. To clarify the source of the proton, molecular dynamics simulations were carried out on solvated ruthenium–substrate complexes (Scheme 74, 1203–1205) using propan-2-ol, formic acid-based, and water systems (Scheme 74b). The theoretical results, both at small and large scales, explained that the origin of the proton strongly depends on the nature of the solvent or hydrogen donor. In less acidic media such as isopropanol, the proton is predominantly generated by the N–H bond of the catalyst, indicating a metal–ligand cooperative mechanism. In contrast, in more acidic systems such as formic acid, water, and HCO_2_H–NEt_3_ mixtures, the proton is mainly generated from the solvent or reagent itself. These findings indicate a direct correlation between solvent acidity and the feasible reaction pathway. More acidic environments favor a chemically innocent pathway, where the solvent acts as the proton donor, whereas less acidic conditions promote a stepwise metal–ligand bifunctional mechanism involving the catalyst N–H bond.

## Outlook and future perspectives

Significant advances have been made in the fields of transition ruthenium-catalyzed TH and ATH, which have developed rapidly in terms of efficient, selective, and sustainable synthetic methodologies. However, we should anticipate several challenging developments in the near future. One of the greatest challenges is the design of new-generation catalysts that demonstrate greater robustness, recyclability, and universal applicability. Although Noyori–Ikariya-type catalysts are already efficient, the target is to create air- and moisture-stable catalysts that exhibit increased activity and selectivity for industrial applications. This necessitates advancements in ligand design to control steric and electronic factors, which will enhance metal–ligand cooperativity and improve catalytic activity, particularly for challenging and unreactive substrates. Future studies will likely focus on expanding the substrate scope to include more complex, functionalized molecules that are sterically hindered. This includes pure aliphatic substrates and strongly coordinating substrates such as nitriles, amines, and heterocycles, which currently have limited catalytic activity. The reduction of these multifunctional molecules is challenging, especially in cascade, tandem, and multicomponent processes combined with ATH, making this promising field with significant potential for growth. There will also be more development in the fields of DKR, parallel kinetic resolution (PKR), and cross-ATH, aiming to create more precise selectivity across multiple active centers. Sustainable development efforts will promote greener and more energy-efficient conditions, utilizing renewable hydrogen donors in aqueous or solvent-free systems with low catalyst loading. Other technological pathways, such as mechanochemical processes, electrocatalysis, hydrogen transfer, and photocatalytic reduction, may prove beneficial for environmental sustainability and atom economy. Mechanistic pathways present another vital area for development, with increased use of *in situ* spectroscopy (flow NMR), computational modelling, and machine learning methods. These will help design more accurate catalytic cycles, transition states, and non-covalent interactions, leading to the rational design of more efficient and selective catalysts. An important aspect of this research will be the application in industrial and pharmaceutical fields, which is essential for growth in this context. Key factors include cost-effectiveness, scalability, and regulatory considerations in the synthesis of drugs and fine chemical materials. The durability of catalytic cycles, continuous-flow processes, and turnover number (TON) are critical to advancing this field. In the future, optimization of ruthenium catalysts for TH and ATH, along with improved mechanistic insights and sustainable development, will be essential for continued innovation.

## Conclusions

This review discusses the TH and ATH processes, which are robust, powerful, selective, and highly sustainable strategies for the synthesis of valuable organic molecules. This study covers recent improvements in ruthenium-catalyzed hydrogen transfer processes for carbonyl compound reduction, with a focus on their efficiency, operational simplicity, and broad applicability in asymmetric synthesis. Ruthenium complexes serve as robust catalysts that exhibit excellent chemo-, regio-and stereocontrol. They demonstrate compatibility with a wide variety of substrates, including α-substituted, heteroatom-containing, cyclic, heteroaromatic and highly functionalized carbonyl compounds, facilitating access to complex organic frameworks. The catalytic activity, regioselectivity, and enantioselectivity are influenced by the qualities of the ruthenium catalyst and ligand framework, substrate coordination behavior, hydrogen donor, and reaction conditions. The catalytic activity of ruthenium systems—including Noyori–Ikariya, NHC, triazole, pincer, and tethered Ru(ii) catalysts—offers high efficiency and selectivity in terms of chemoselectivity, regioselectivity, diastereoselectivity, and enantioselectivity. A significant advancement has been made in the substrate scope, enabling the use of structurally complex and challenging systems such as cyclobutenediones, unsymmetrical diketones, acylboronates, fluorinated ketones, α-hydroxyenones, sterically hindered compounds, and various heteroaromatic compounds. These substrates can achieve high yields and outstanding stereoselectivities. In terms of sustainability, greener hydrogen sources, such as alcohols, formic acid, paraformaldehyde, and ammonia borane, are suitable for this transformation. Additionally, the use of environmentally friendly solvents, base-free conditions, low catalyst loadings, and the mechanochemical process has gained considerable attention for industrial applications. Mechanistic studies, including controlled experiments, deuterium-labelling experiments, kinetic studies, and density functional theory (DFT) simulations, provided deep insight into hydrogen-transfer pathways, catalyst-substrate interactions, and the origins of stereoselectivity. These studies explain the metal–ligand bifunctional mechanism, in which hydride transfer from the metal occurs alongside proton transfer either from the ligand (N–H) or the reaction medium. Non-covalent interactions, such as CH–π interactions, hydrogen bonding, and lone pair–π effects, play a crucial role in stabilizing the transition state and determining the configuration and enantioselectivity. The review highlights the usefulness of these catalytic methods for synthesizing complex molecules by discussing sample synthetic processes for producing physiologically active compounds and structurally complicated organic molecules. The balance between asymmetric reduction and *in situ* racemization is particularly important for reactions involving configurationally labile substrates, as it helps achieve significant ee and product yield. Factors such as temperature, catalyst loading, and reaction duration significantly affect catalytic performance and product distribution in chemical reactions. Insights provided in this review aim to guide the rational design of new ruthenium-based catalytic systems, focusing on mechanistically informed development of reactions to broaden their applications in synthesizing biologically relevant and structurally complex molecules. Despite recent advances, challenges remain in increasing catalyst efficiency at low loadings, improving applications for less activated and highly functionalized substrates, addressing catalyst deactivation by strongly coordinating functional groups, enhancing reactivity of sterically hindered or purely aliphatic substrates, and achieving precise selectivity in molecules with multiple reactive sites. Developing a universal catalyst design remains a challenge, along with improving catalyst recyclability, scalability, and energy efficiency for future research. Future research focused on mechanistically driven catalyst design, sustainability, and scalable methodologies is anticipated to enhance the synthesis of enantioenriched and structurally complex molecules.

## Conflicts of interest

The authors declare that there are no conflicts of interest.

## Data Availability

No new data were created or analyzed in this study. Data sharing is not applicable to this article.
